# Supplementary material for: The Palette of Science and Emotions: Art-Based Learning With Structured Peer Role-Plays for Early Clinical Exposure in Biochemistry
Source: MedEdPORTAL. 2026 May 19;22:11601. doi: 10.15766/mep_2374-8265.11601 (PMC13183865; doi:10.15766/mep_2374-8265.11601)
Supplement: Supplementary file 1 — Faculty Orientation.pptxCurated Artworks.docxActivity Instructions.docxRole-Play Resources.docxFacilitator Guide.docxPersonal Reflection Questionnaire.docxEvaluation Questionnaire.docxSemistructured Interview Guide.docxPostsession Assessment.docxConfidence Questionnaire.docx [file mep_2374-8265.11601-s001.zip › C. Activity Instructions.docx]

**Instructions**

**Early Clinical Exposure: Art Based Learning in Biochemistry**

1. Students will be divided into small groups of **five** in each. There will be a total of **30 groups.**
2. Each group will be allotted a faculty facilitator to guide the process.
3. This activity consists of four parts: Visual Thinking Strategies (VTS) session, Structured Peer Roleplay, Group Reflection and Plenary Session.
4. The total time for the activity is **180 minutes.** The time will be divided as follows:

- Visual Thinking Strategies (VTS) session: 30 minutes
- Structured Peer Roleplay: 45 minutes.
- Group Reflection: 25 minutes

Plenary Session & post session assessment: 60-80 minutes

1. The faculty will facilitate the VTS session.
2. Each group will be given a file consisting of the following: Artwork, Structured Peer Roleplay clinical case scenario, roles in roleplay, key clinical and biochemical points to discuss, dilemma to resolve and reflection questions. This will be followed by description of five roles in the roleplay.
3. The session starts with VTS. Facilitators will instruct the students about VTS and its process, followed by an actual VTS session. This activity will be conducted for 30 minutes.
4. The aim of this session is to enhance the observational and reflective ability of the students. Students are encouraged to observe deeply the artwork given to them. They are asked not just to look at the obvious features, but to notice emotions, subtle details, posture, expressions, and hidden elements in the scene. They are guided to interpret what they see, to imagine the story behind the image, and to reflect on how emotions, environment, and illness interact.
5. After the VTS session, the groups will engage in the structured peer roleplay.
6. Every group will have 5 students and will work with one clinical scenario given in the file that is connected to the artwork they observed.
7. One textbook per group is permitted
8. Every student in the group will be given a specific role such as doctor, patient, family member, nurse, or counsellor. This can be done by volunteering or random allocation by the faculty.
9. Students are expected to act and interact based on their assigned roles, thinking as their character would in a real clinical situation and resolve the dilemmas.
10. In the unlikely situation of a group falling short of a member for the 5 roles, the facilitator can play a role to accommodate. If more than 1 member are lacking, the groups can accommodate within themselves to have a student play dual role.
11. The aim of the this activity is to promote communication, empathy, teamwork, and clinical reasoning.
12. This activity will be conducted for 45 minutes. Take adequate time to understand your role and keep the conversation rich and engaging to make the roleplay realistic and meaningful.
13. It is the responsibility of the faculty to facilitate this and ensure students are interacting as per their roles and cover all parts of the activity.
14. Following this, each group will reflect on the questions given in the file (25 minutes)
15. This debriefing will be facilitated by the faculty. At the end of reflection, each student will fill in the online reflection form.
16. The online form is the “I used to think, now I think and now I will….” type of reflection.
17. The aim of this is to encourage personal reflection on emotions, the concept of suffering, and communication. This reflection helps students recognize how their initial understanding of illness and patient experiences may have been limited, and how observation, role play, and interaction have shaped a deeper and more empathetic view and how they can bring greater sensitivity and awareness into their future clinical practice.
18. Finally, the students will complete the evaluation and confidence questionnaire online.
19. In the final 60-80 minutes, groups will pair together (groups with same artwork & case scenario)
20. Present their case summary and the biochemical reasoning for the conditions given in the case
21. After all groups present the cases, a post-session assessment with 15 reasoning type of question. (Short answer type)
