# Supplementary material for: The Palette of Science and Emotions: Art-Based Learning With Structured Peer Role-Plays for Early Clinical Exposure in Biochemistry
Source: MedEdPORTAL. 2026 May 19;22:11601. doi: 10.15766/mep_2374-8265.11601 (PMC13183865; doi:10.15766/mep_2374-8265.11601)
Supplement: Supplementary file 1 — Faculty Orientation.pptxCurated Artworks.docxActivity Instructions.docxRole-Play Resources.docxFacilitator Guide.docxPersonal Reflection Questionnaire.docxEvaluation Questionnaire.docxSemistructured Interview Guide.docxPostsession Assessment.docxConfidence Questionnaire.docx [file mep_2374-8265.11601-s001.zip › E. Facilitator Guide.docx]

**Facilitator Guide**

**Name of Facilitator:**

**Group No:**

**No of Students:**

**Structured Peer Roleplay & Reflection Activity Checklist**

| **Phase** | **Task** | **Tick after completion** |
| --- | --- | --- |
| **Structured Peer Roleplay** | Distribute roles to each group (doctor, patient, family member, nurse, counsellor etc.) |  |
|  | Read out the scenario and also allow 5–10 minutes for the group to read rest of the structured peer roleplay materials silently |  |
|  | Ask each student to describe about their role to the other members for everyone to have clear understanding |  |
|  | Encourage students to view the textbook for relevant content according to their case scenario. |  |
|  | Allow students to interact as a group for about 30 minutes and resolve the dilemmas |  |
|  | Encourage natural, respectful dialogue and teamwork |  |
|  | Ensure all learning objectives are discussed |  |
|  | Observe group dynamics, empathy, and role engagement |  |
|  | Take brief notes to guide the debriefing discussion |  |
| **Reflection** | Facilitate the reflection through the questions given in the file. Ensure all members reflect on all questions. |  |
|  | Invite each student to write their reflection: “I used to think... now I think... now I will...” |  |
|  | Guide students to reflect on emotions, communication, and patient perspective |  |
|  | Facilitate a short debrief using: What happened, So what, What next |  |
|  | Ensure all students complete the online reflection, confidence and evaluation forms |  |

**Facilitator Role Reminders**

- Be a gentle facilitator, not a judge
- Guide discussions with empathy, curiosity, and open-mindedness
- Support students who are shy or unsure during reflection
- Remind students this is about learning human skills, not getting it “right”
