# Supplementary material for: The Palette of Science and Emotions: Art-Based Learning With Structured Peer Role-Plays for Early Clinical Exposure in Biochemistry
Source: MedEdPORTAL. 2026 May 19;22:11601. doi: 10.15766/mep_2374-8265.11601 (PMC13183865; doi:10.15766/mep_2374-8265.11601)
Supplement: Supplementary file 1 — Faculty Orientation.pptxCurated Artworks.docxActivity Instructions.docxRole-Play Resources.docxFacilitator Guide.docxPersonal Reflection Questionnaire.docxEvaluation Questionnaire.docxSemistructured Interview Guide.docxPostsession Assessment.docxConfidence Questionnaire.docx [file mep_2374-8265.11601-s001.zip › F. Personal Reflection Questionnaire.docx]

**Personal Reflection**

**(Created as SurveyMonkey link and shared the online link with students)**

**1. I used to think...**

**2. Now I think...**

**3. Now I will...**
