# Supplementary material for: The Palette of Science and Emotions: Art-Based Learning With Structured Peer Role-Plays for Early Clinical Exposure in Biochemistry
Source: MedEdPORTAL. 2026 May 19;22:11601. doi: 10.15766/mep_2374-8265.11601 (PMC13183865; doi:10.15766/mep_2374-8265.11601)
Supplement: Supplementary file 1 — Faculty Orientation.pptxCurated Artworks.docxActivity Instructions.docxRole-Play Resources.docxFacilitator Guide.docxPersonal Reflection Questionnaire.docxEvaluation Questionnaire.docxSemistructured Interview Guide.docxPostsession Assessment.docxConfidence Questionnaire.docx [file mep_2374-8265.11601-s001.zip › G. Evaluation Questionnaire.docx]

**Evaluation Questionnaire**

1. **The Visual Thinking Strategies session helped me notice emotions, suffering, and important details in the artwork.**

- Strongly Disagree
- Disagree
- Neutral
- Agree
- Strongly Agree

1. **The discussion helped me understand the emotions shown in the artwork more deeply.**

- Strongly Disagree
- Disagree
- Neutral
- Agree
- Strongly Agree

1. **The session helped me think beyond what is visible and imagine the patient's experience.**

- Strongly Disagree
- Disagree
- Neutral
- Agree
- Strongly Agree

1. **Listening to others’ observations gave me new ways of seeing and thinking.**

- Strongly Disagree
- Disagree
- Neutral
- Agree
- Strongly Agree

1. **The VTS session helped me appreciate how observation skills are important in clinical practice.**

- Strongly Disagree
- Disagree
- Neutral
- Agree
- Strongly Agree

1. **The structured peer roleplay helped me understand the emotions and suffering experienced by patients and families.**

- Strongly Disagree
- Disagree
- Neutral
- Agree
- Strongly Agree

1. **Taking on a specific role helped me think more clearly about real clinical situations.**

- Strongly Disagree
- Disagree
- Neutral
- Agree
- Strongly Agree

1. **The interaction with other roles made the structured peer roleplay feel realistic and engaging.**

- Strongly Disagree
- Disagree
- Neutral
- Agree
- Strongly Agree

1. **I was able to communicate and collaborate effectively with my group during the structured peer roleplay.**

- Strongly Disagree
- Disagree
- Neutral
- Agree
- Strongly Agree

1. **The structured peer roleplay helped me practice empathy and active listening.**

- Strongly Disagree
- Disagree
- Neutral
- Agree
- Strongly Agree

1. **The clinical problem linked to the artwork made me think deeply about real life patient care.**

- Strongly Disagree
- Disagree
- Neutral
- Agree
- Strongly Agree

1. **The activity helped me understand how biochemistry knowledge applies in real patient experiences.**

- Strongly Disagree
- Disagree
- Neutral
- Agree
- Strongly Agree

1. **The activity helped me understand the underlying biochemical mechanisms responsible for the patient’s clinical features.**

- Strongly Disagree
- Disagree
- Neutral
- Agree
- Strongly Agree

1. **The reflection exercise helped me become more aware of my original thoughts and assumptions.**

- Strongly Disagree
- Disagree
- Neutral
- Agree
- Strongly Agree

1. **The reflection helped me notice how my understanding of illness and communication changed after the session.**

- Strongly Disagree
- Disagree
- Neutral
- Agree
- Strongly Agree

1. **The “I used to think, now I think, now I will” format made me think about how I will improve in future.**

- Strongly Disagree
- Disagree
- Neutral
- Agree
- Strongly Agree

1. **The reflection made me realize the importance of understanding patient emotions, suffering, and communication skills in real practice.**

- Strongly Disagree
- Disagree
- Neutral
- Agree
- Strongly Agree

1. **Compared to traditional lecture-based sessions in Biochemistry, this activity improved my confidence in applying biochemical concepts to clinical scenarios.**

- Strongly Disagree
- Disagree
- Neutral
- Agree
- Strongly Agree

1. **Compared to small group case-based learning in ECE, this activity enhanced my engagement and depth of understanding.**

- Strongly Disagree
- Disagree
- Neutral
- Agree
- Strongly Agree

1. **Overall, the session helped me connect observation, empathy, communication, and clinical thinking in a meaningful way.**

- Strongly Disagree
- Disagree
- Neutral
- Agree
- Strongly Agree
