# Supplementary material for: The Palette of Science and Emotions: Art-Based Learning With Structured Peer Role-Plays for Early Clinical Exposure in Biochemistry
Source: MedEdPORTAL. 2026 May 19;22:11601. doi: 10.15766/mep_2374-8265.11601 (PMC13183865; doi:10.15766/mep_2374-8265.11601)
Supplement: Supplementary file 1 — Faculty Orientation.pptxCurated Artworks.docxActivity Instructions.docxRole-Play Resources.docxFacilitator Guide.docxPersonal Reflection Questionnaire.docxEvaluation Questionnaire.docxSemistructured Interview Guide.docxPostsession Assessment.docxConfidence Questionnaire.docx [file mep_2374-8265.11601-s001.zip › J. Confidence Questionnaire.docx]

**Confidence Questionnaire**

**How confident are you in the following areas?**
(*Rate each item* ***before*** *and* ***after*** *the session using the 1–5 scale*)

- **1 = Not at all confident**
- **2 = Slightly confident**
- **3 = Moderately confident**
- **4 = Very confident**
- **5 = Extremely confident**

| **Area** | **Before the Session** | **After the Session** |
| --- | --- | --- |
| 1. Observing and noticing important details when looking at patient situations | [1–5] | [1–5] |
| 2. Understanding and responding to patient emotions | [1–5] | [1–5] |
| 3. Thinking from the patient and family perspective during clinical encounters | [1–5] | [1–5] |
| 4. Communicating clearly and sensitively during difficult conversations | [1–5] | [1–5] |
| 5. Working with others as a team during clinical discussions | [1–5] | [1–5] |
| 6. Applying my basic science knowledge like biochemistry to patient care | [1–5] | [1–5] |
| 7. Reflecting on my own thinking and emotions to improve my learning | [1–5] | [1–5] |
