# Supplementary material for: The Palette of Science and Emotions: Art-Based Learning With Structured Peer Role-Plays for Early Clinical Exposure in Biochemistry
Source: MedEdPORTAL. 2026 May 19;22:11601. doi: 10.15766/mep_2374-8265.11601 (PMC13183865; doi:10.15766/mep_2374-8265.11601)
Supplement: Supplementary file 1 — Faculty Orientation.pptxCurated Artworks.docxActivity Instructions.docxRole-Play Resources.docxFacilitator Guide.docxPersonal Reflection Questionnaire.docxEvaluation Questionnaire.docxSemistructured Interview Guide.docxPostsession Assessment.docxConfidence Questionnaire.docx [file mep_2374-8265.11601-s001.zip › B. Curated Artwork.docx]

**ART 1**


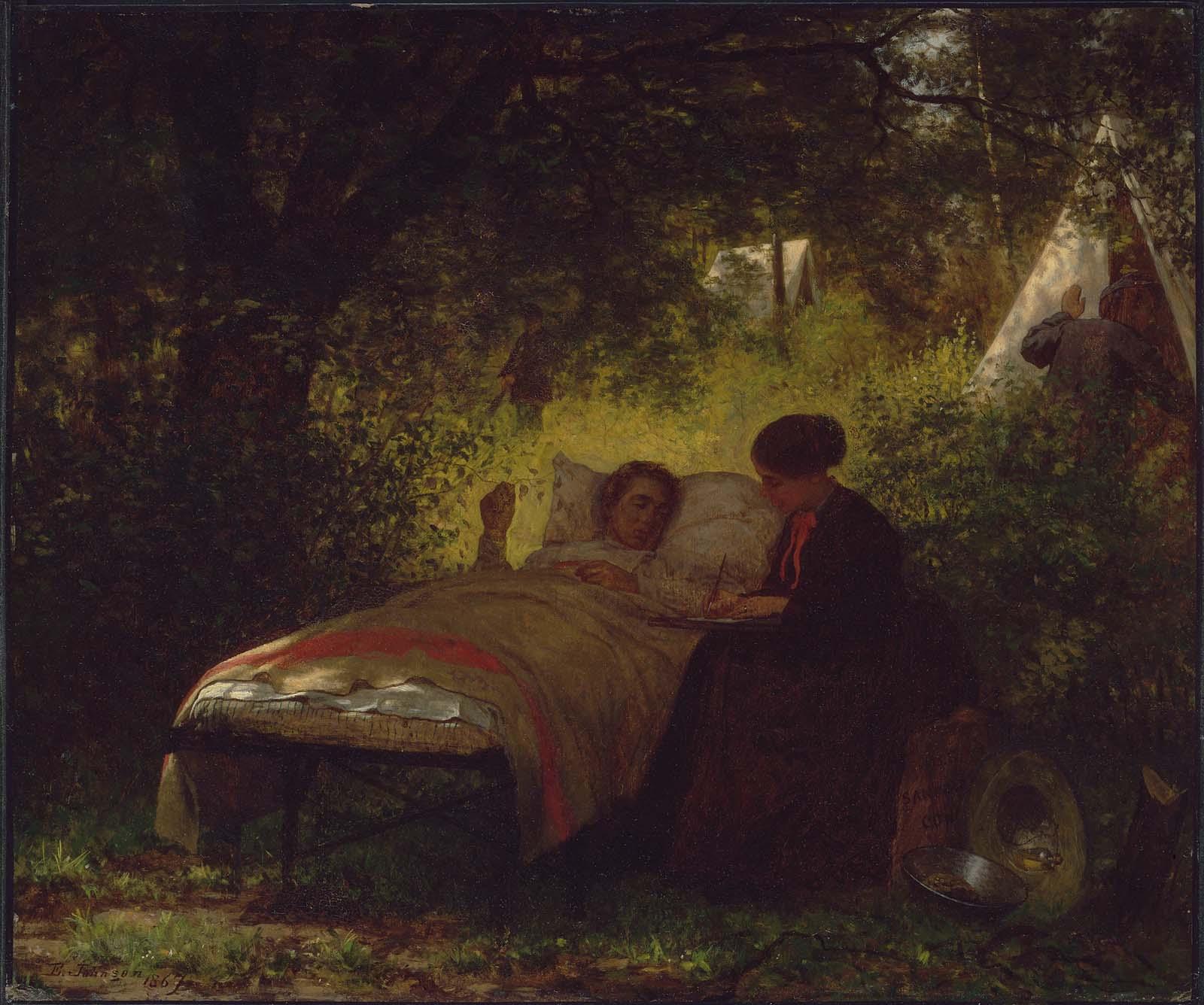


**The Field Hospital By Eastman Johnson (American, 1824–1906) 1867**

Image by [EASTMAN JOHNSON], retrieved from: [https://commons.wikimedia.org/wiki/File:Eastman_Johnson_-_The_Field_Hospital_-_48.434_-_Museum_of_Fine_Arts.jpg] on [12/09/2025]. Image is in the public domain

**ART 2**


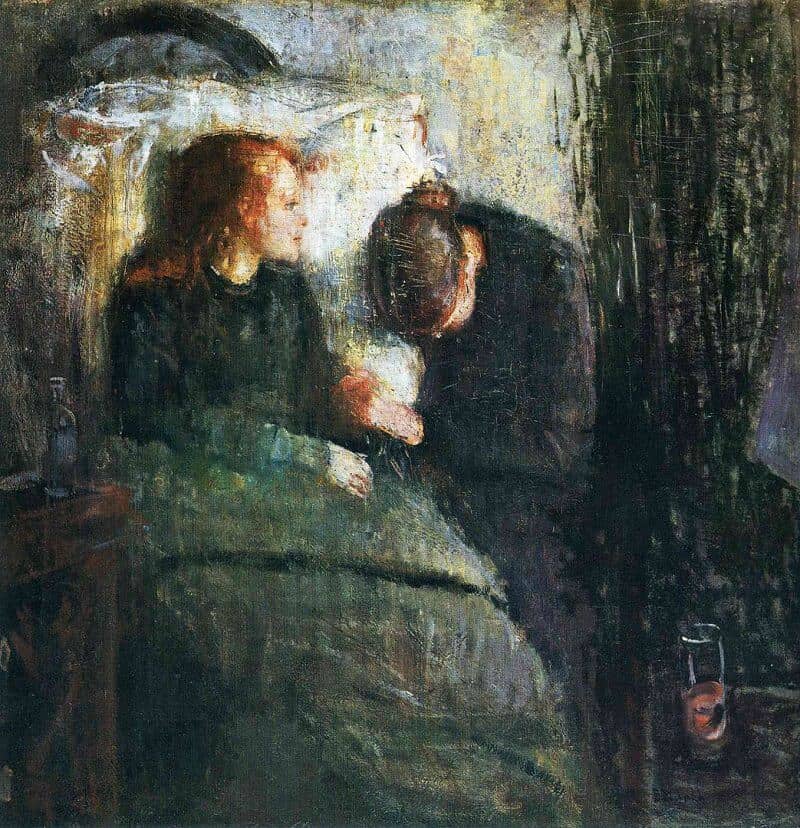


**The Sick Child, 1885 by Edvard Munch**

Image by [EDVARD MUNCH], retrieved from: [<https://commons.wikimedia.org/wiki/File:Edvard_Munch_-_The_Sick_Child_-_NG.M.00839_-_National_Museum_of_Art,_Architecture_and_Design.jpg>] on [12/09/2025]. Image is in the public domain

**ART 3**


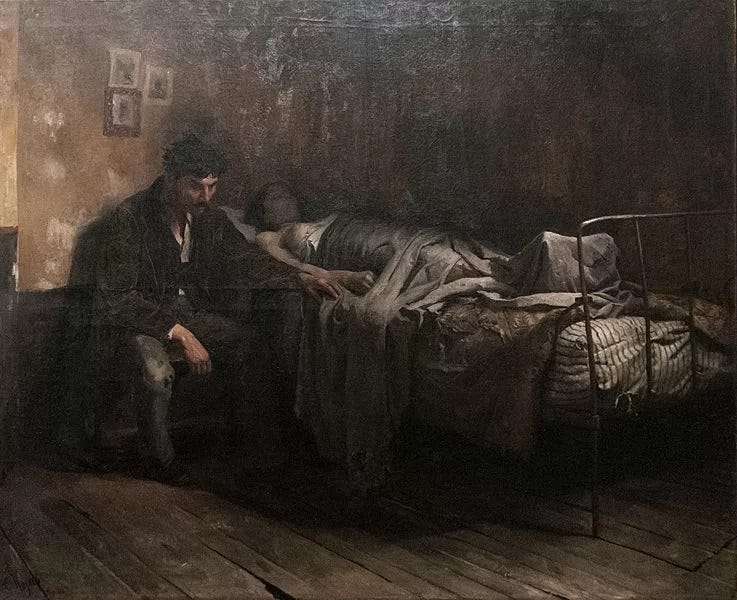


**La Miseria (1886). Cristobal Rojas**

Image by [CRISTOBAL ROJAS], retrieved from: [<https://commons.wikimedia.org/wiki/File:La_miseria_(1886)._Cristobal_Rojas.jpg>] on [12/09/2025]. Image is in the public domain

**ART 4**


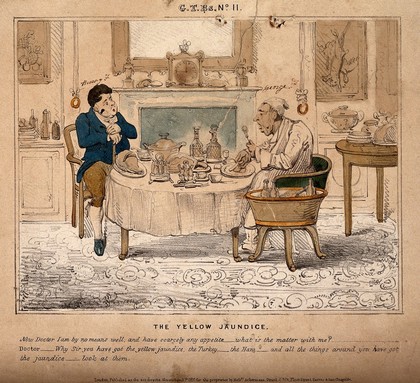


**The Yellow Jaundice, Coloured lithograph, 1835.**

“Image by [WELLCOME TRUST], retrieved from: [<https://commons.wikimedia.org/wiki/File:A_man_surrounded_by_a_feast_while_professing_to_have_no_appe_Wellcome_V0010997.jpg>] on [12/09/2025]. Creative Commons License associated: [https://creativecommons.org/licenses/by/2.0/deed.en].”

**ART – 5**


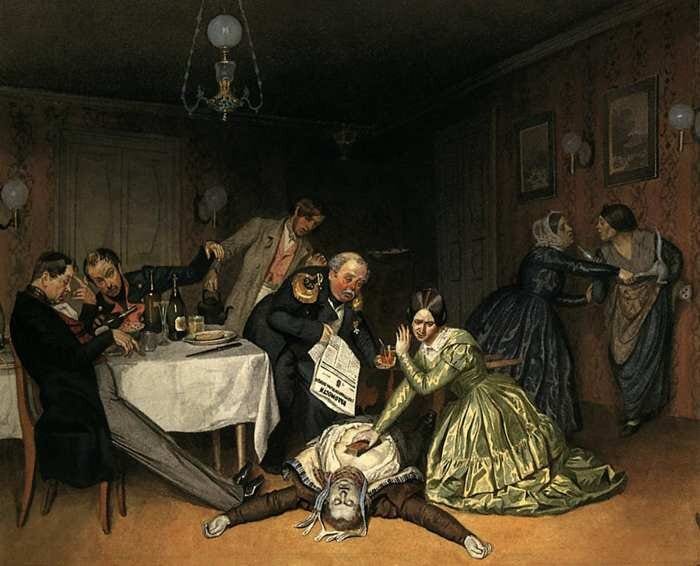


**Pavel Fedotov’s, mid 19^th^ century**

Image by [PAVEL FEDOTOV], retrieved from: [<https://www.wikiart.org/en/pavel-fedotov/it-is-cholera-to-blame>] on [12/09/2025]. Image is in the public domain

**ART 6**


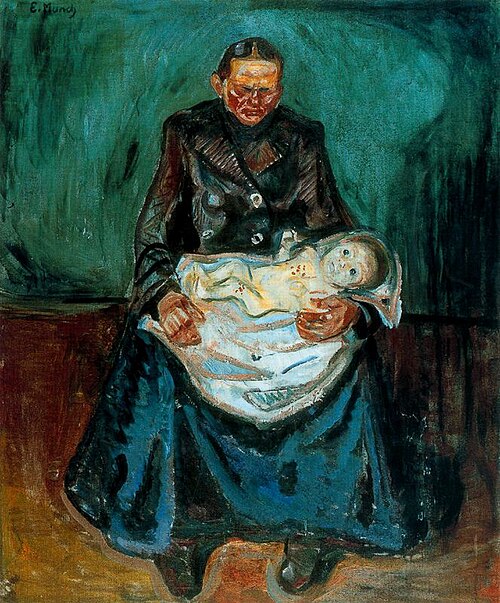


***Inheritance* (Munch), 1877-1899**

Image by [EDVARD MUNCH], retrieved from: [<https://commons.wikimedia.org/wiki/File:Edvard_Munch_-_Inheritance.jpg>] on [12/09/2025]. Image is in the public domain

**ART – 7**


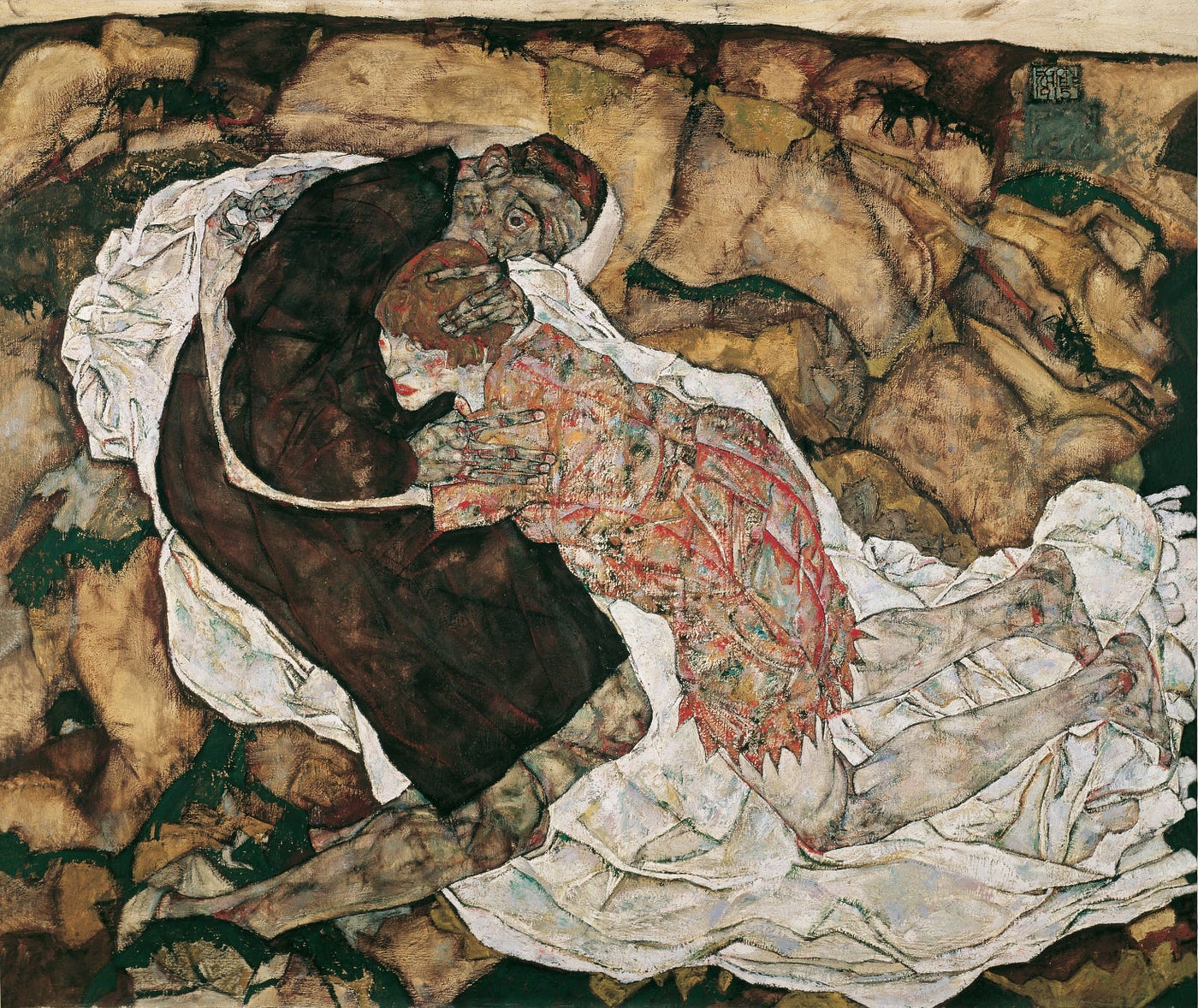


**Death and the Maiden (Schiele) 1915**

Image by [EGON SCHIELE], retrieved from: [<https://commons.wikimedia.org/wiki/File:Egon_Schiele_012.jpg>] on [12/09/2025]. Image is in the public domain

**ART – 8**

**
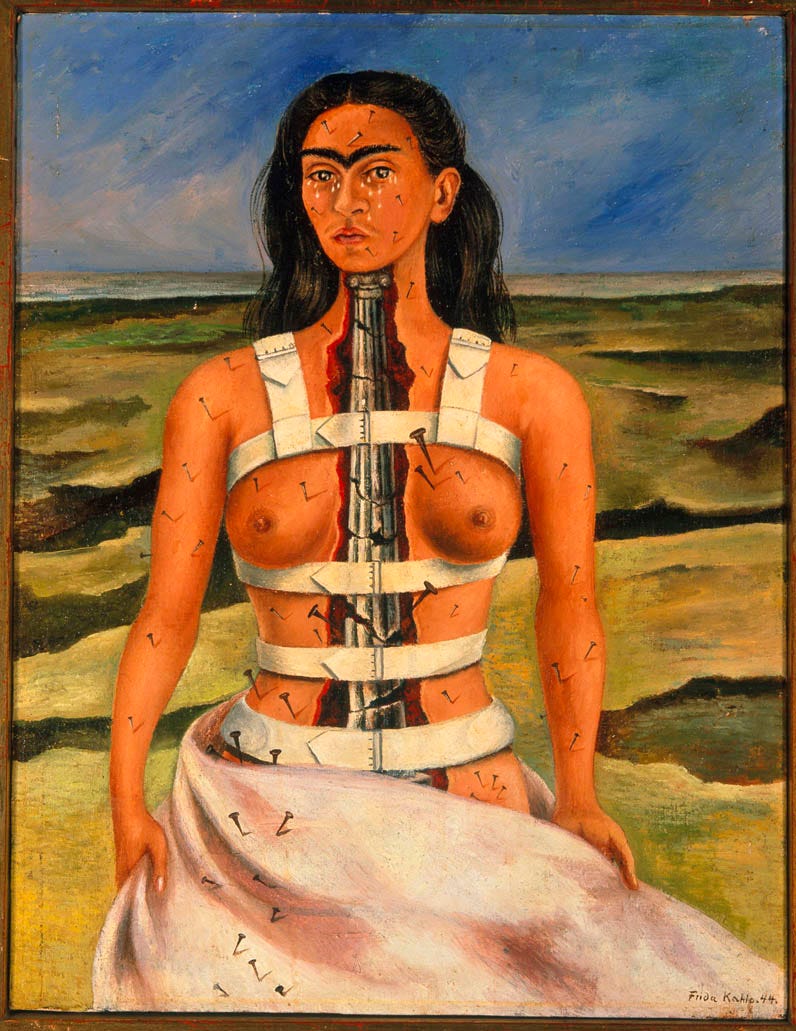
**

**Frida Kahlo “The Broken Column” (1944)**

Image by [FRIDA KAHLO], retrieved from: [<https://www.wikiart.org/en/frida-kahlo/the-broken-column-1944>] on [12/09/2025]. Image is in the public domain

**ART – 9**

**
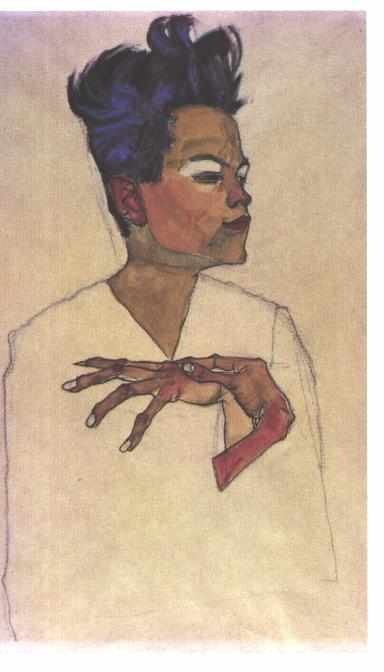
**

**Egon Schiele – Self-Portrait with Hands on Chest (1910)**

Image by [EGON SCHIELE], retrieved from: [https://commons.wikimedia.org/wiki/File:Schiele_-_Selbstbildnis_mit_Händen_vor_der_Brust.jpg] on [12/09/2025]. Image is in the public domain

**ART – 10**


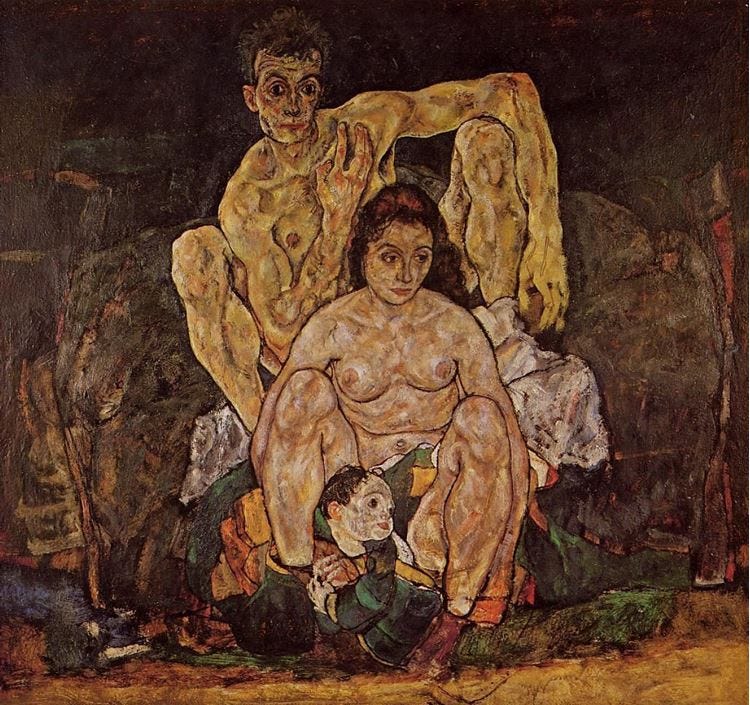


**The Family / 1918 — Egon Schiele**

Image by [EGON SCHIELE], retrieved from: [https://commons.wikimedia.org/wiki/File:Egon_Schiele_-_Kauerndes_Menschenpaar_(Die_Familie)_-_4277_-_Österreichische_Galerie_Belvedere.jpg] on [12/09/2025]. Image is in the public domain

**ART – 11**

**
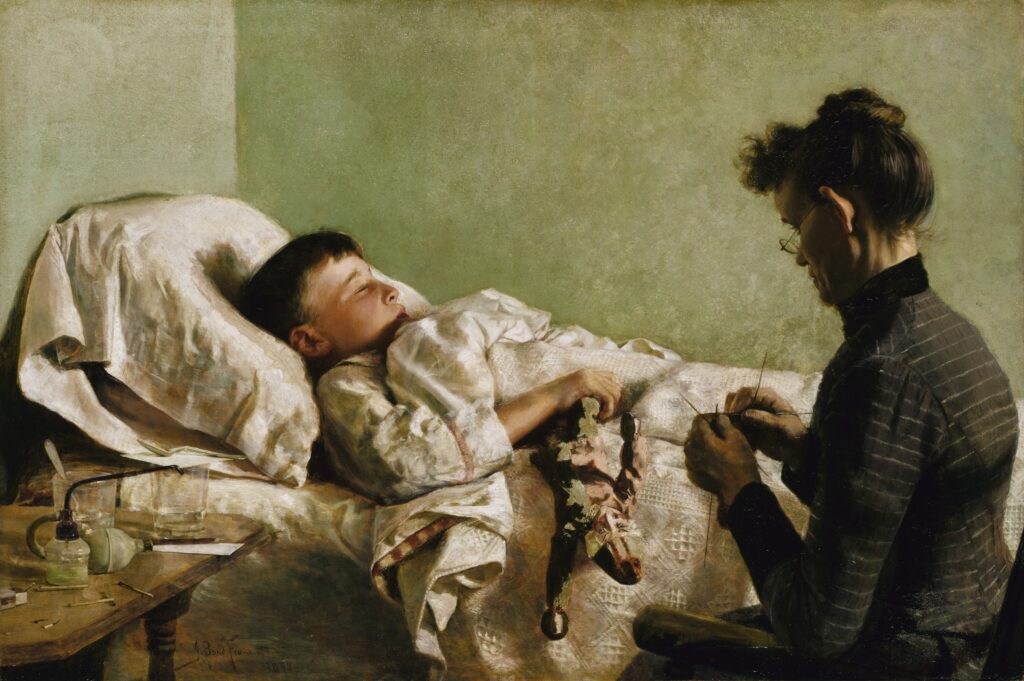
**

**John Bond Francisco, The Sick Child, 1893**

Image by [JOHN BOND FRANCISCO], retrieved from: [<https://commons.wikimedia.org/wiki/File:J._Bond_Francisco_-_The_Sick_Child_-_1991.9_-_Smithsonian_American_Art_Museum.jpg>] on [12/09/2025]. Image is in the public domain

**ART – 12**

**
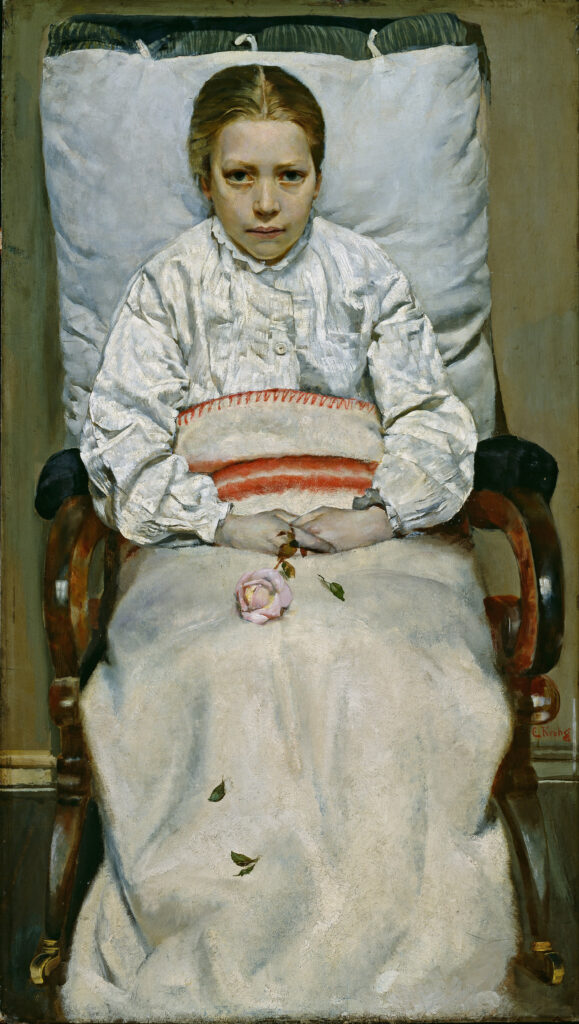
**

**Christian Krohg, Sick Girl, 1880–1881**

Image by [CHRISTIAN KROHG], retrieved from: [<https://commons.wikimedia.org/wiki/File:Sick-girl-christian-krohg-1881.jpg>] on [12/09/2025]. Image is in the public domain

**ART – 13**

**
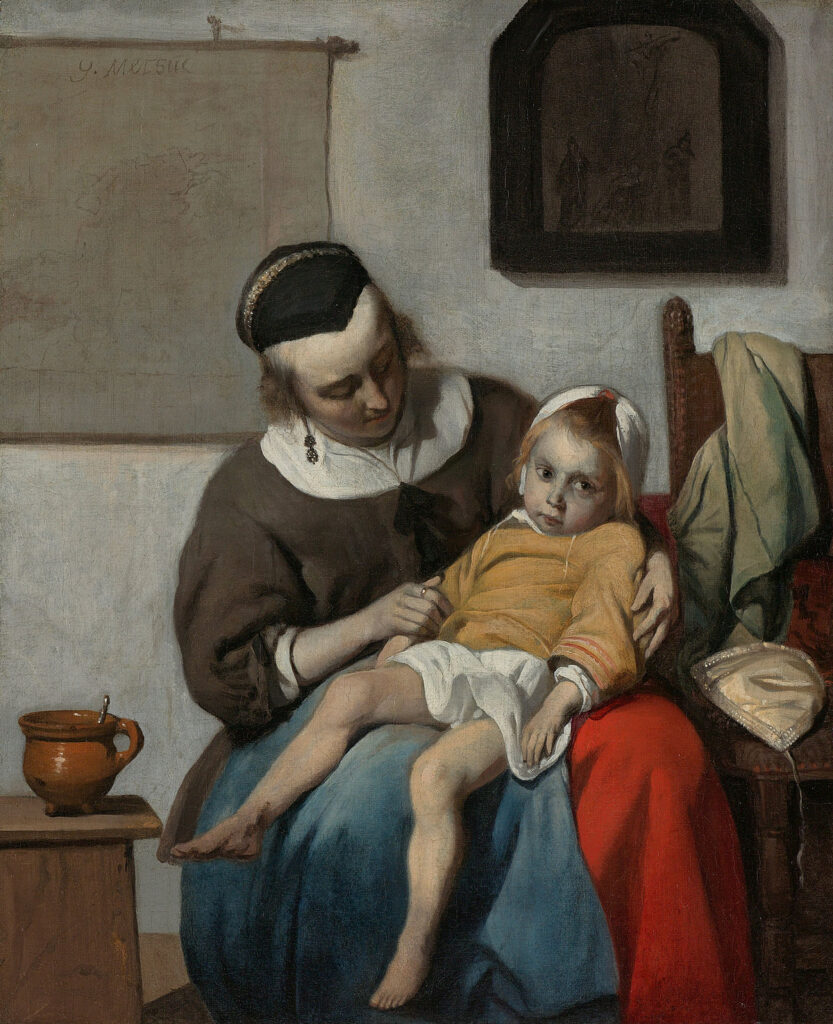
**

- **Gabriël Metsu, The Sick Child, ca 1663–1664,**

Image by [GABRIËL METSU], retrieved from: [<https://commons.wikimedia.org/wiki/File:Gabriël_Metsu_-_Het_zieke_kind_-_Google_Art_Project.jpg>] on [12/09/2025]. Image is in the public domain

**ART – 14**


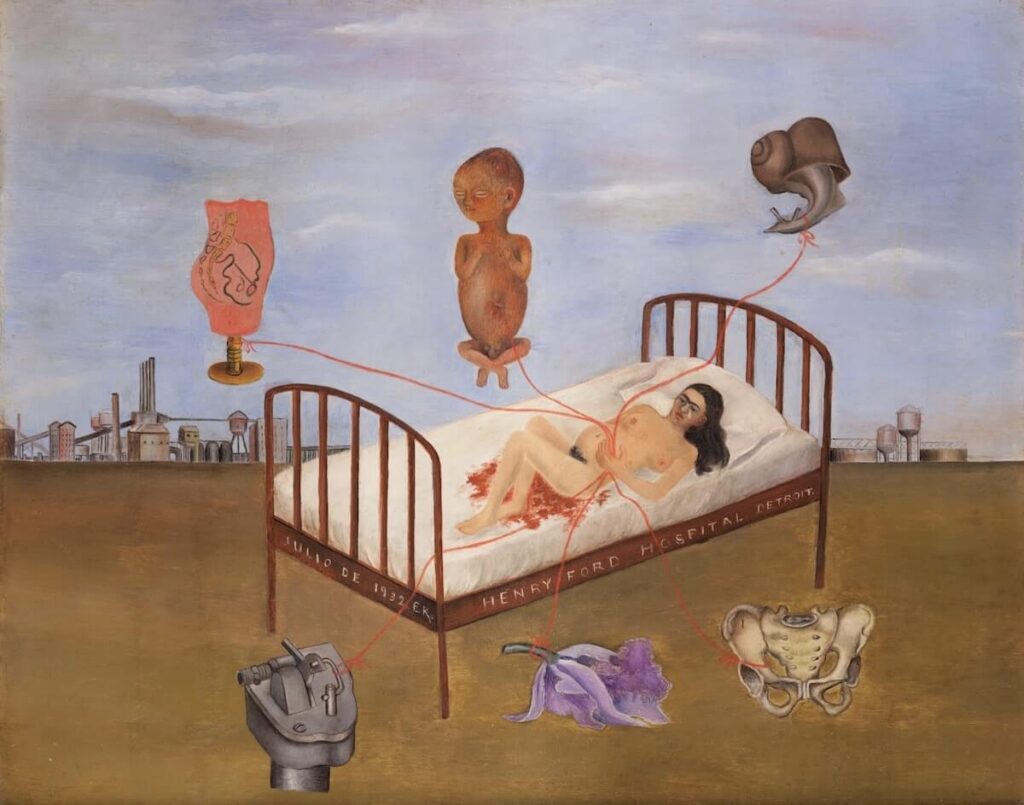


**Frida Kahlo, Henry Ford Hospital, 1932,**

Image by [FRIDA KAHLO], retrieved from: [<https://www.wikiart.org/en/frida-kahlo/henry-ford-hospital-the-flying-bed-1932>] on [12/09/2025]. Image is in the public domain

**ART 15**


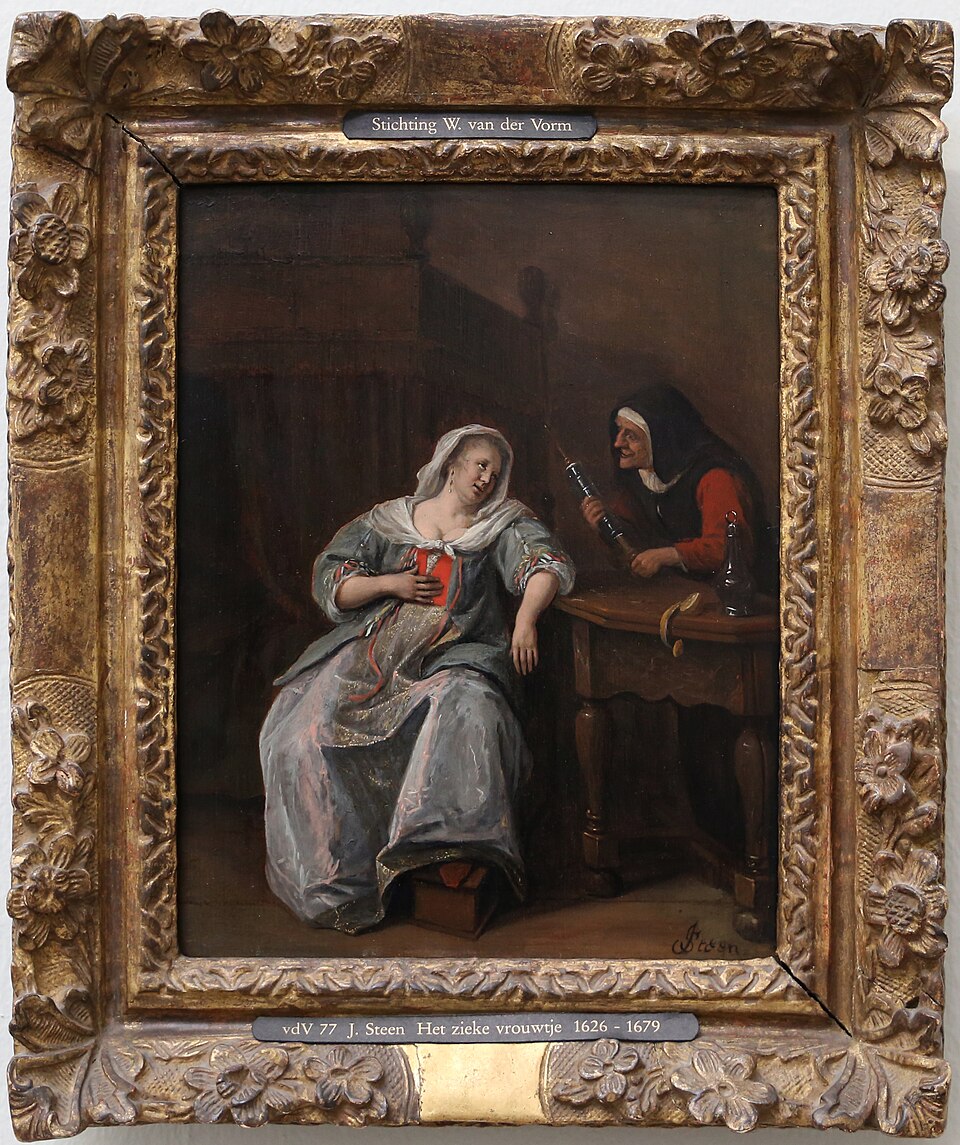


**Jan Steen, *The Sick Woman*, ca. 1660, Museum Boijmans van Beuningen, Rotterdam, Netherlands**

“Image by [SAILKO], retrieved from: [<https://commons.wikimedia.org/wiki/File:Jan_steen,_la_malata,_1660_ca.jpg>] on [12/09/2025]. Creative Commons License associated: [https://creativecommons.org/licenses/by/3.0/deed.en].”
