# Supplementary material for: The Palette of Science and Emotions: Art-Based Learning With Structured Peer Role-Plays for Early Clinical Exposure in Biochemistry
Source: MedEdPORTAL. 2026 May 19;22:11601. doi: 10.15766/mep_2374-8265.11601 (PMC13183865; doi:10.15766/mep_2374-8265.11601)
Supplement: Supplementary file 1 — Faculty Orientation.pptxCurated Artworks.docxActivity Instructions.docxRole-Play Resources.docxFacilitator Guide.docxPersonal Reflection Questionnaire.docxEvaluation Questionnaire.docxSemistructured Interview Guide.docxPostsession Assessment.docxConfidence Questionnaire.docx [file mep_2374-8265.11601-s001.zip › A. Faculty Orientation.pptx]

## Slide 1
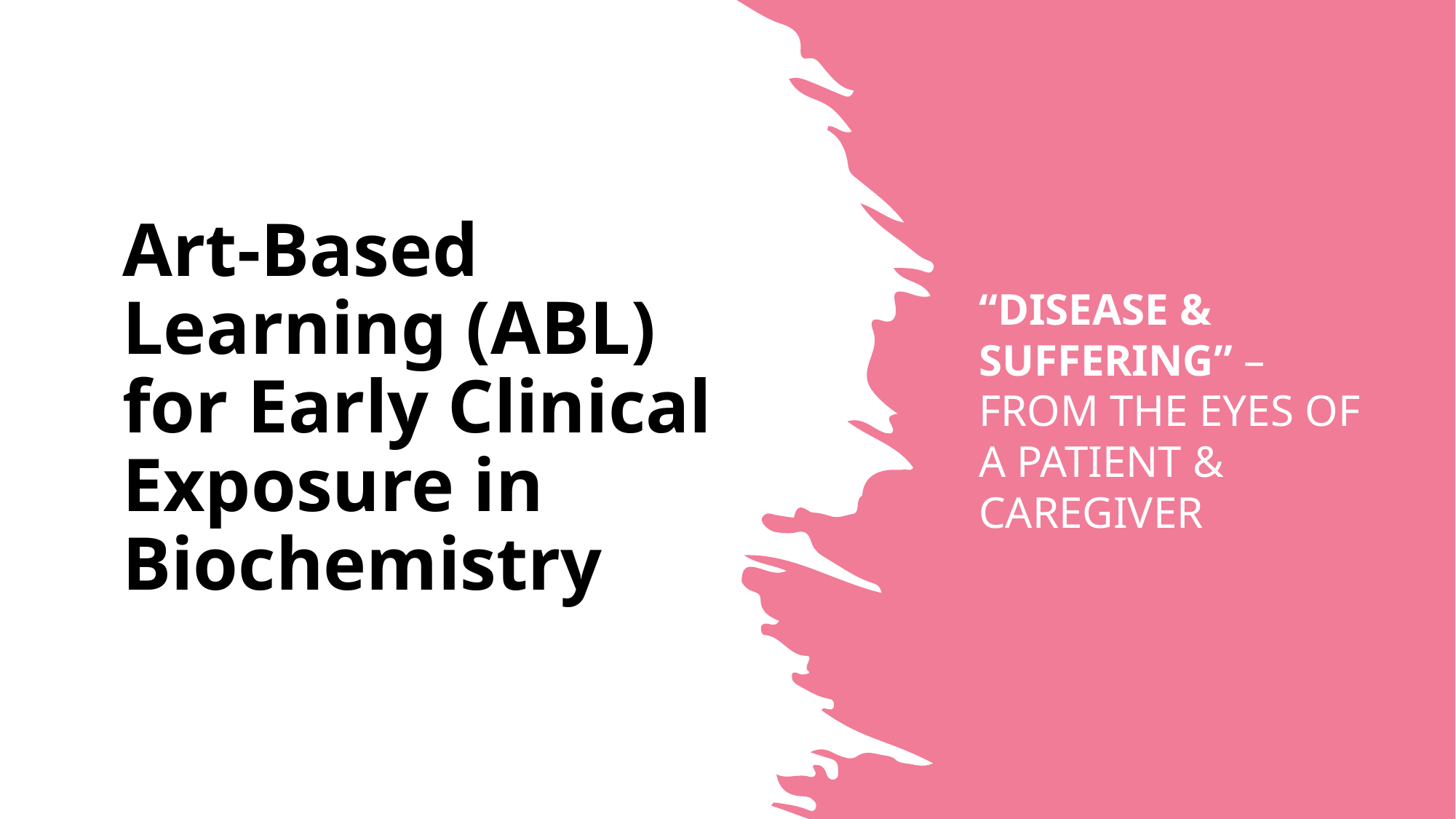

# Art-Based Learning (ABL) for Early Clinical Exposure in Biochemistry
“Disease & SUFFERING” – FROM THE EYES OF A PATIENT & CAREGIVER

## Slide 2
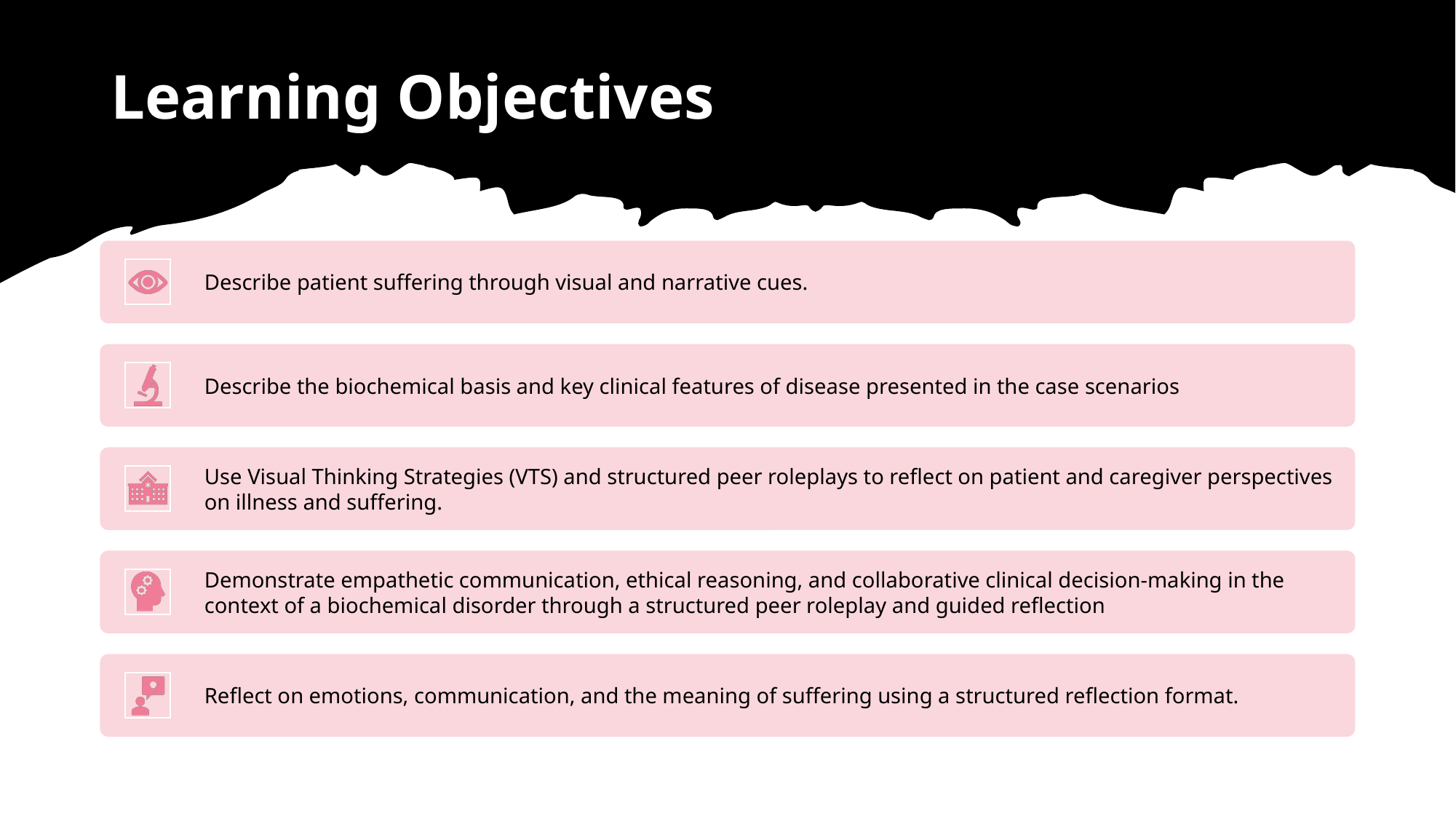

# Learning Objectives
By the end of this session, students will be able to

## Slide 3
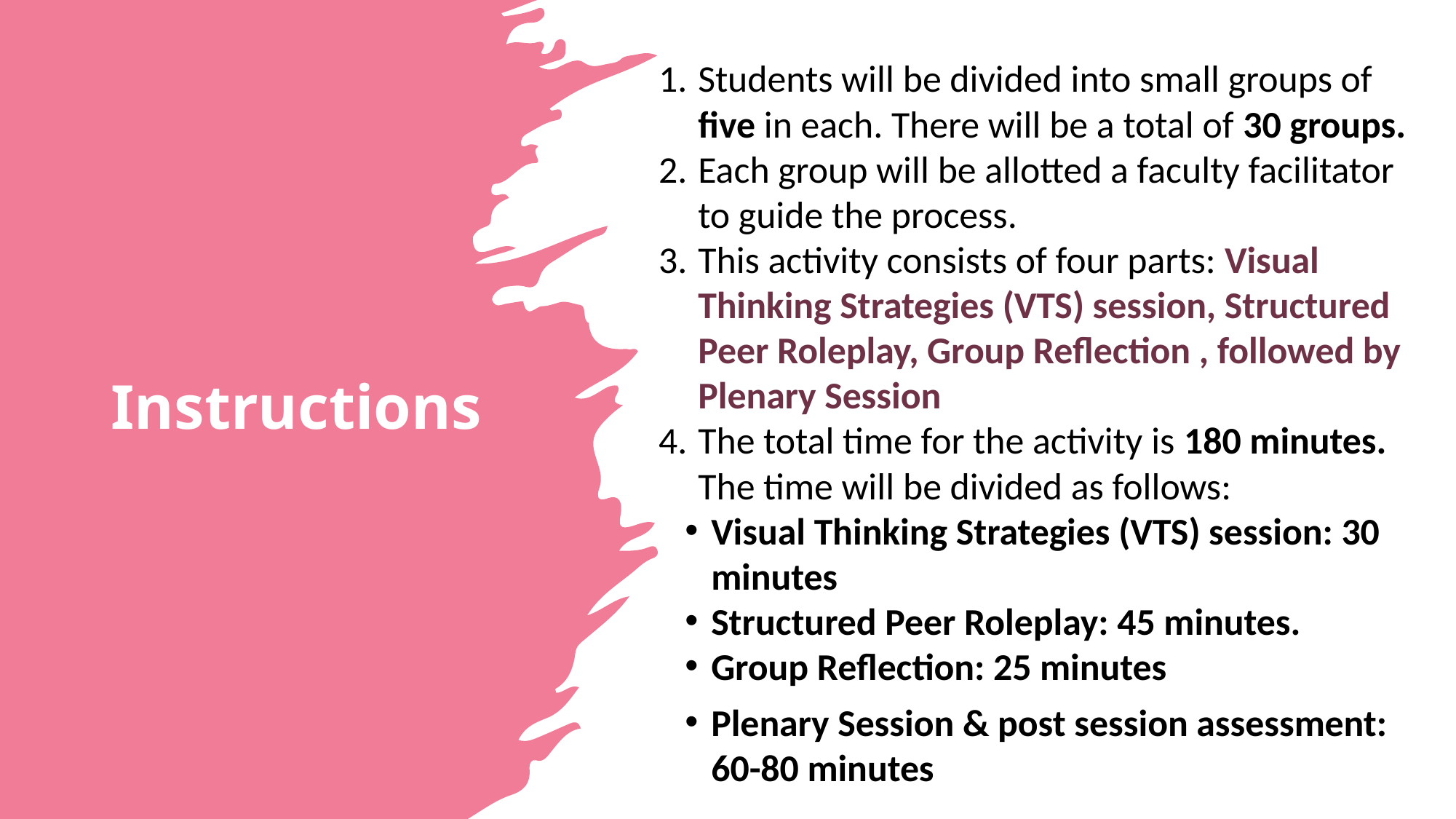

Students will be divided into small groups of five in each. There will be a total of 30 groups.
Each group will be allotted a faculty facilitator to guide the process.
This activity consists of four parts: Visual Thinking Strategies (VTS) session, Structured Peer Roleplay, Group Reflection , followed by Plenary Session
The total time for the activity is 180 minutes. The time will be divided as follows:
Visual Thinking Strategies (VTS) session: 30 minutes
Structured Peer Roleplay: 45 minutes.
Group Reflection: 25 minutes
Plenary Session & post session assessment: 60-80 minutes
# Instructions

## Slide 4
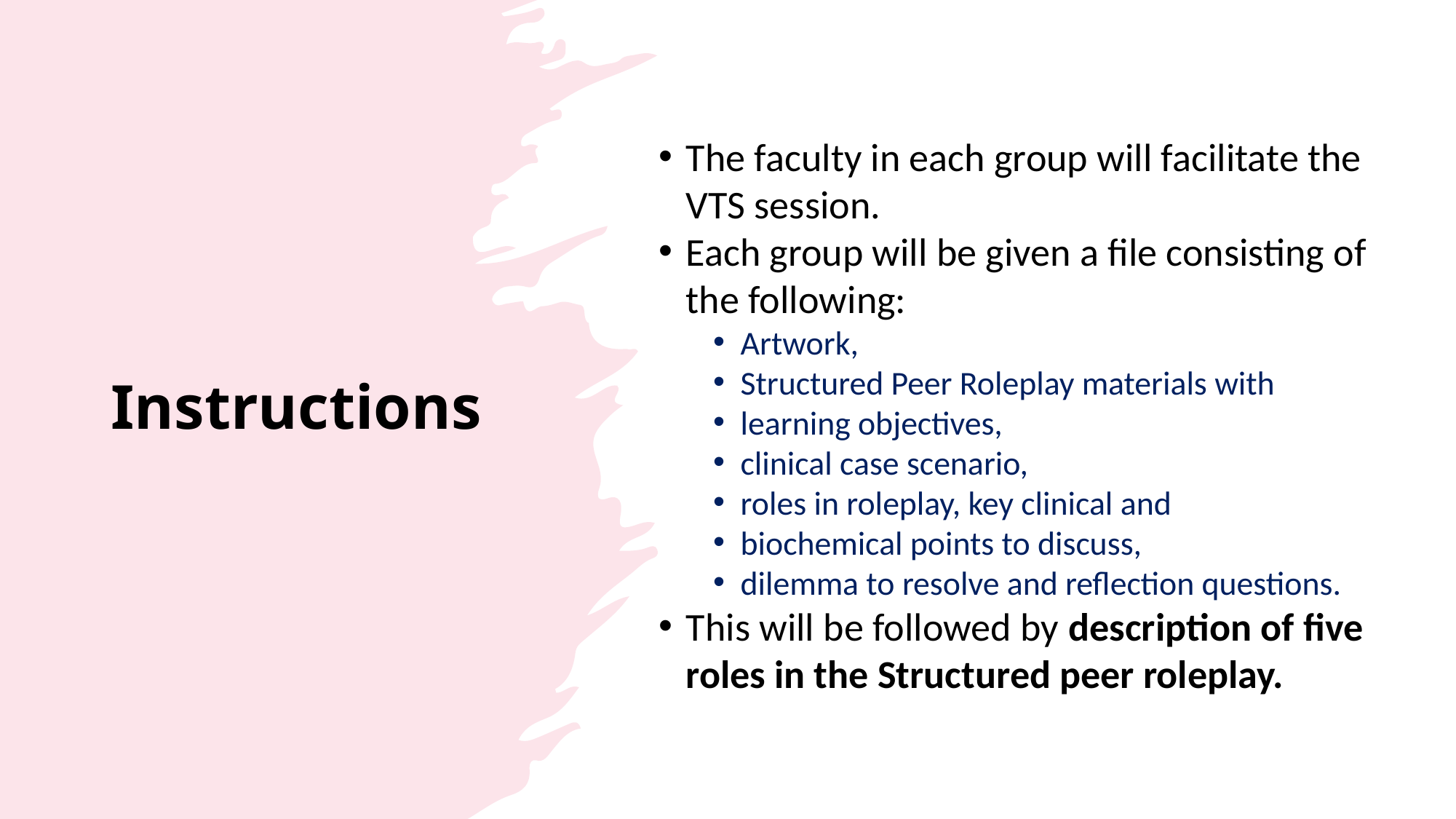

The faculty in each group will facilitate the VTS session.
Each group will be given a file consisting of the following:
Artwork,
Structured Peer Roleplay materials with
learning objectives,
clinical case scenario,
roles in roleplay, key clinical and
biochemical points to discuss,
dilemma to resolve and reflection questions.
This will be followed by description of five roles in the Structured peer roleplay.
# Instructions

## Slide 5
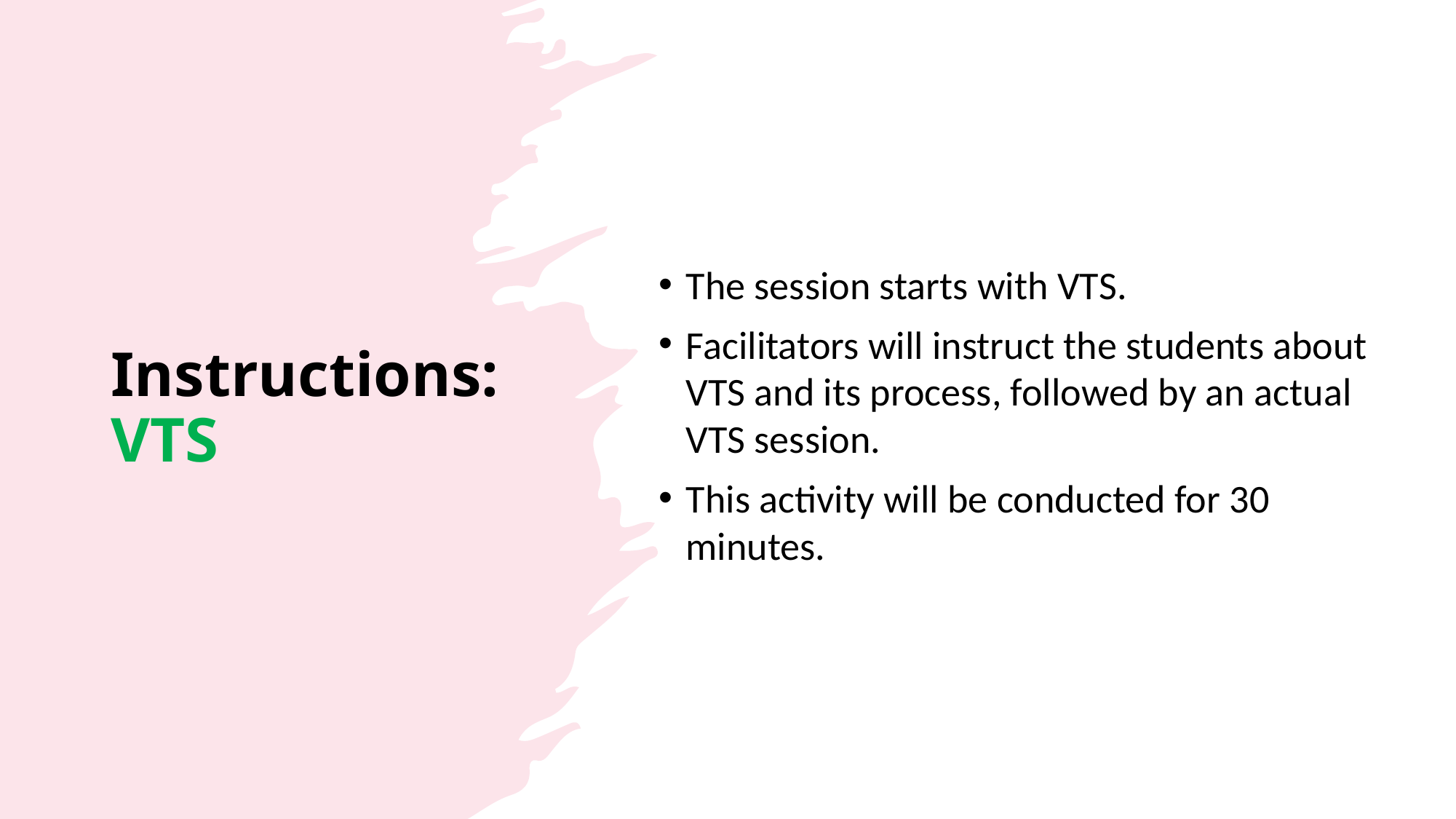

The session starts with VTS.
Facilitators will instruct the students about VTS and its process, followed by an actual VTS session.
This activity will be conducted for 30 minutes.
# Instructions: VTS

## Slide 6
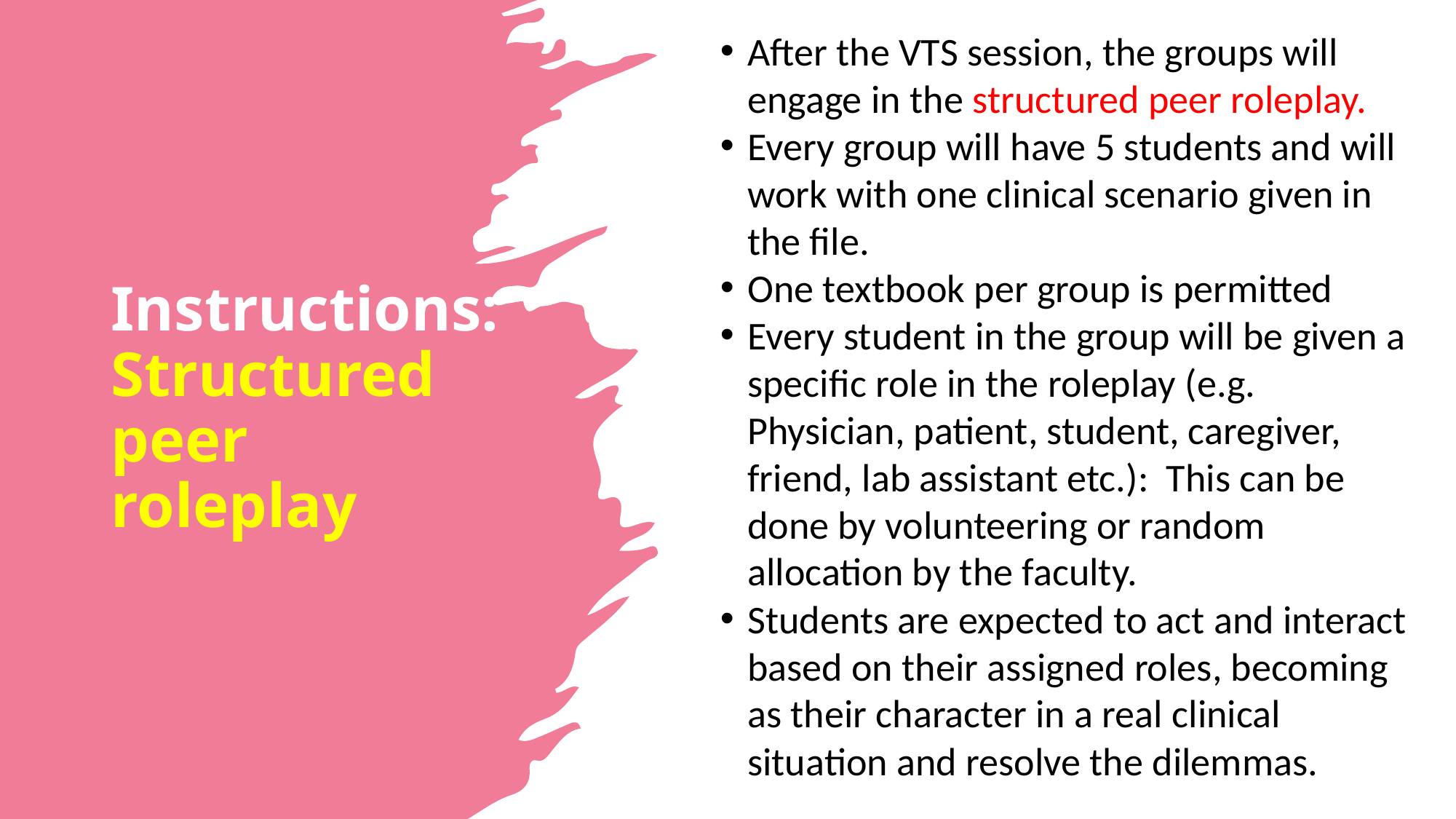

# Instructions: Structured peer roleplay
After the VTS session, the groups will engage in the structured peer roleplay.
Every group will have 5 students and will work with one clinical scenario given in the file.
One textbook per group is permitted
Every student in the group will be given a specific role in the roleplay (e.g. Physician, patient, student, caregiver, friend, lab assistant etc.): This can be done by volunteering or random allocation by the faculty.
Students are expected to act and interact based on their assigned roles, becoming as their character in a real clinical situation and resolve the dilemmas.

## Slide 7
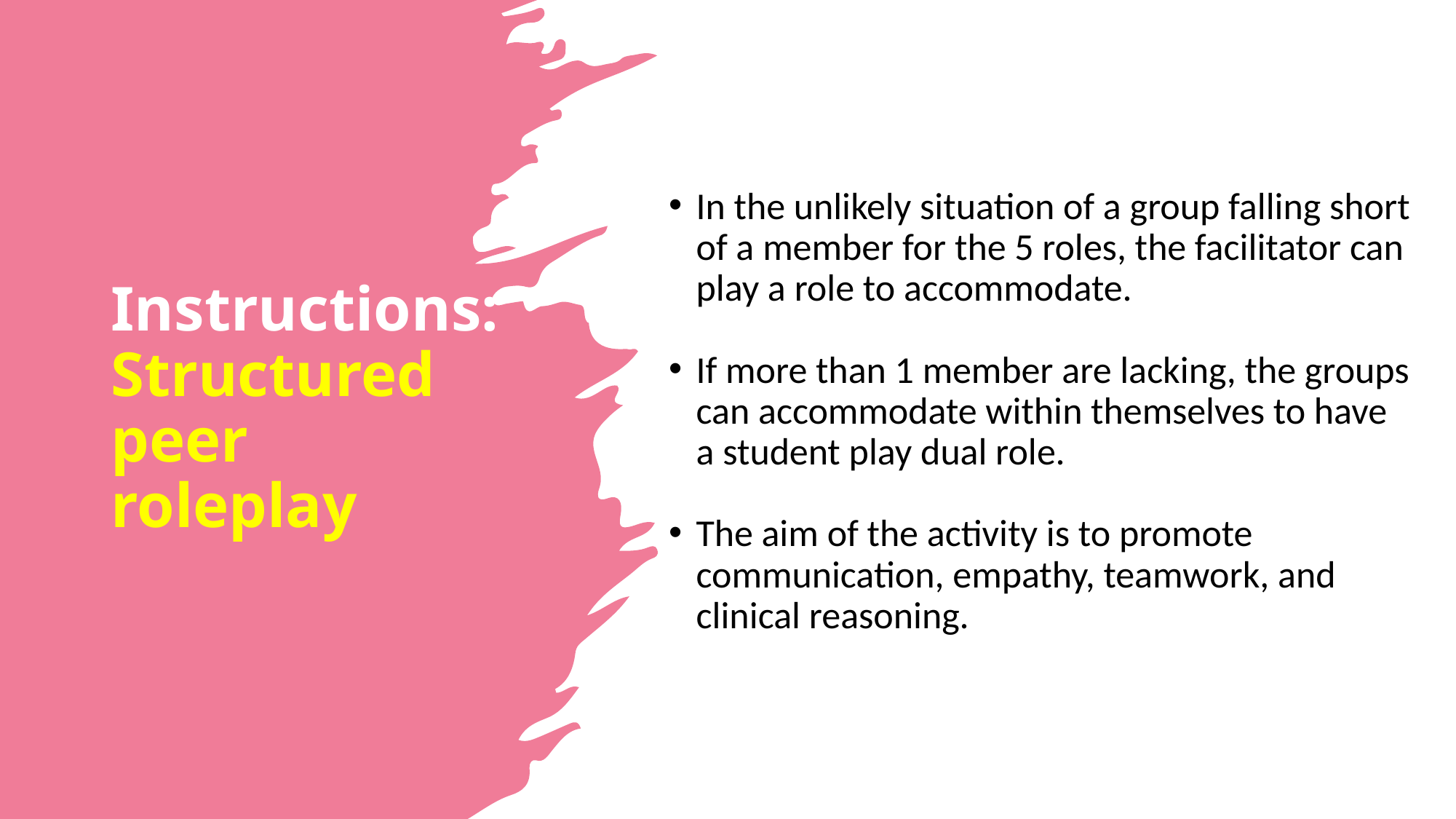

# Instructions: Structured peer roleplay
In the unlikely situation of a group falling short of a member for the 5 roles, the facilitator can play a role to accommodate.
If more than 1 member are lacking, the groups can accommodate within themselves to have a student play dual role.
The aim of the activity is to promote communication, empathy, teamwork, and clinical reasoning.

## Slide 8
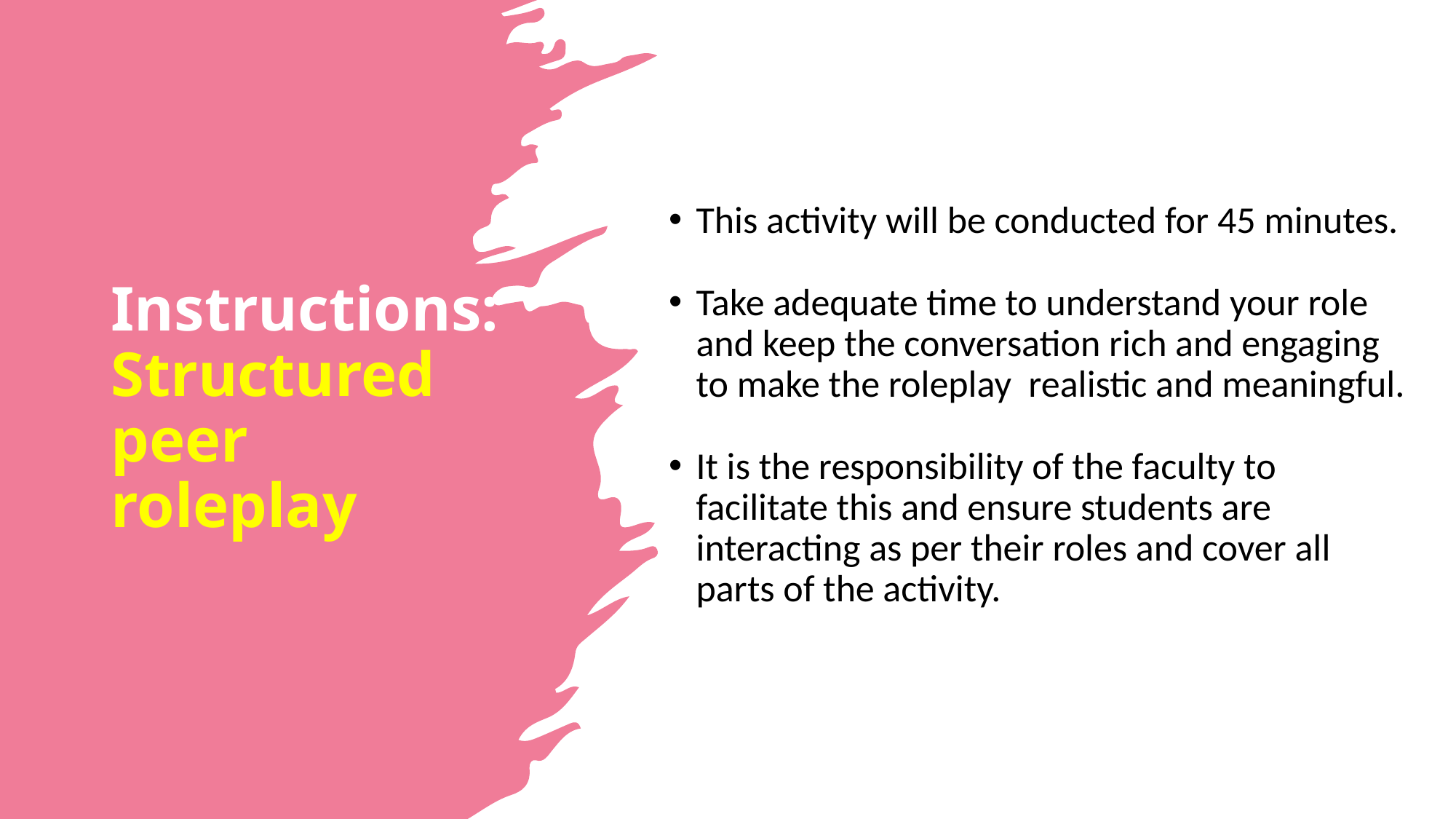

# Instructions: Structured peer roleplay
This activity will be conducted for 45 minutes.
Take adequate time to understand your role and keep the conversation rich and engaging to make the roleplay realistic and meaningful.
It is the responsibility of the faculty to facilitate this and ensure students are interacting as per their roles and cover all parts of the activity.

## Slide 9
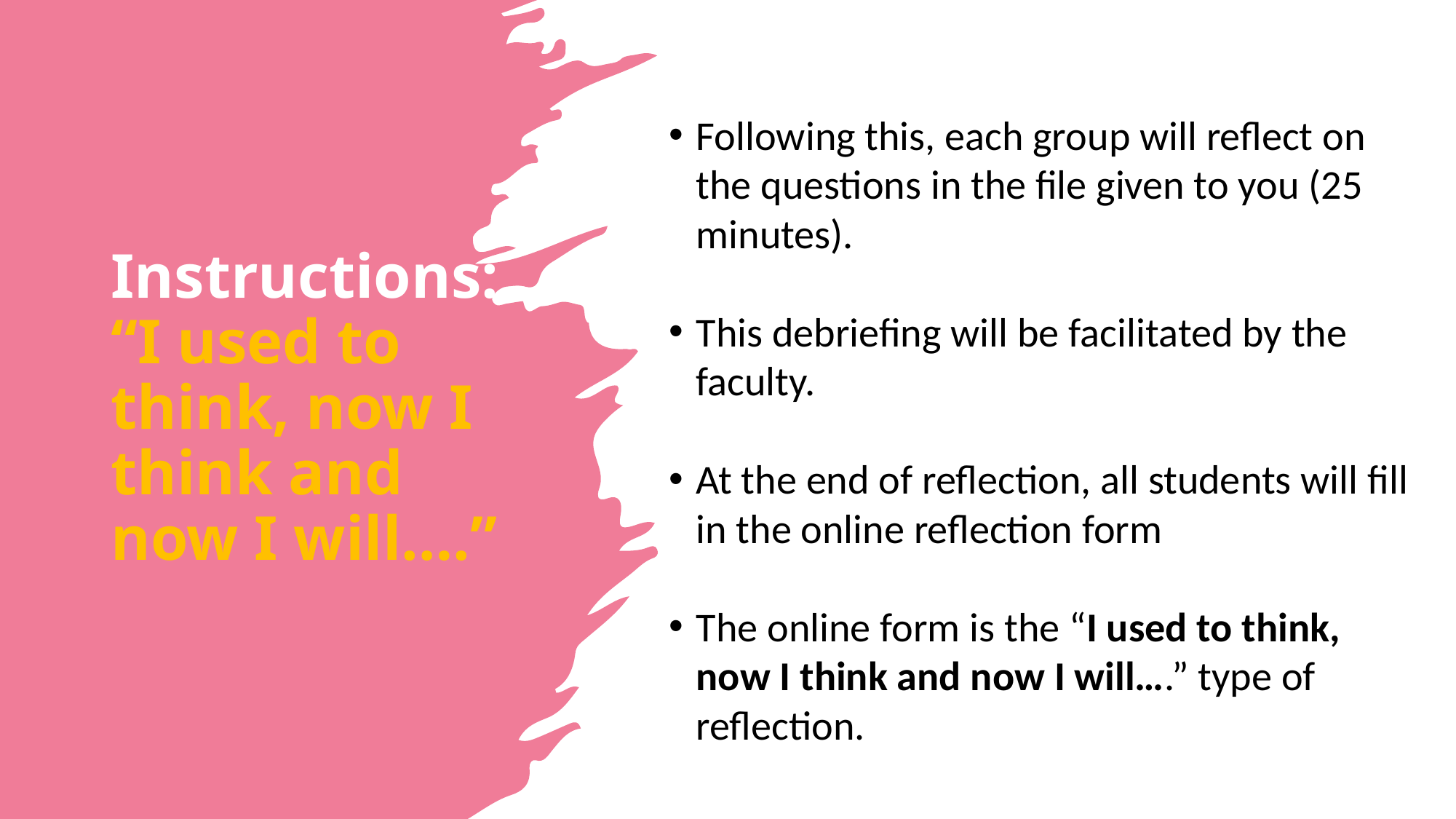

Following this, each group will reflect on the questions in the file given to you (25 minutes).
This debriefing will be facilitated by the faculty.
At the end of reflection, all students will fill in the online reflection form
The online form is the “I used to think, now I think and now I will….” type of reflection.
# Instructions: “I used to think, now I think and now I will….”

## Slide 10
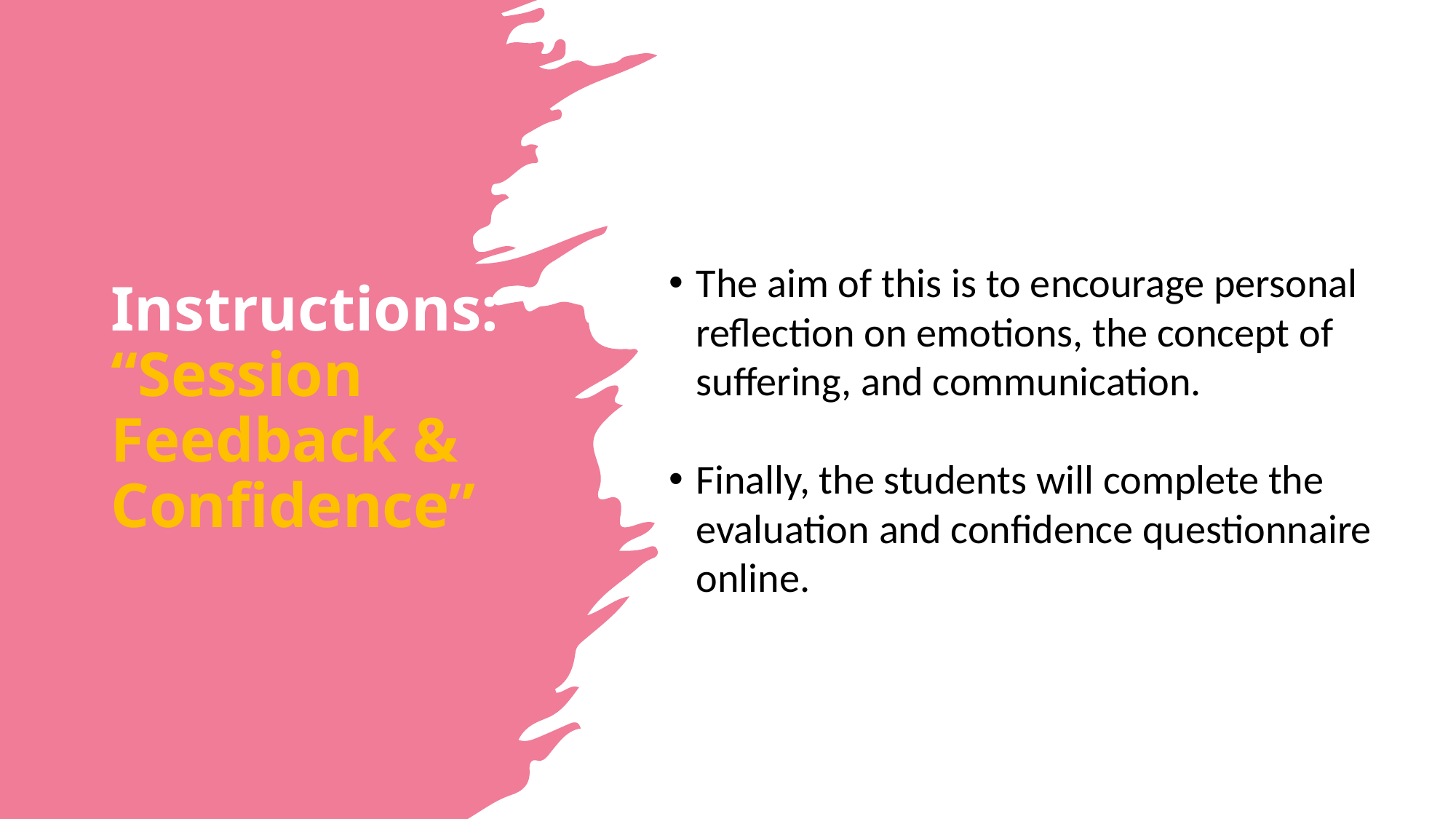

The aim of this is to encourage personal reflection on emotions, the concept of suffering, and communication.
Finally, the students will complete the evaluation and confidence questionnaire online.
# Instructions: “Session Feedback & Confidence”

## Slide 11
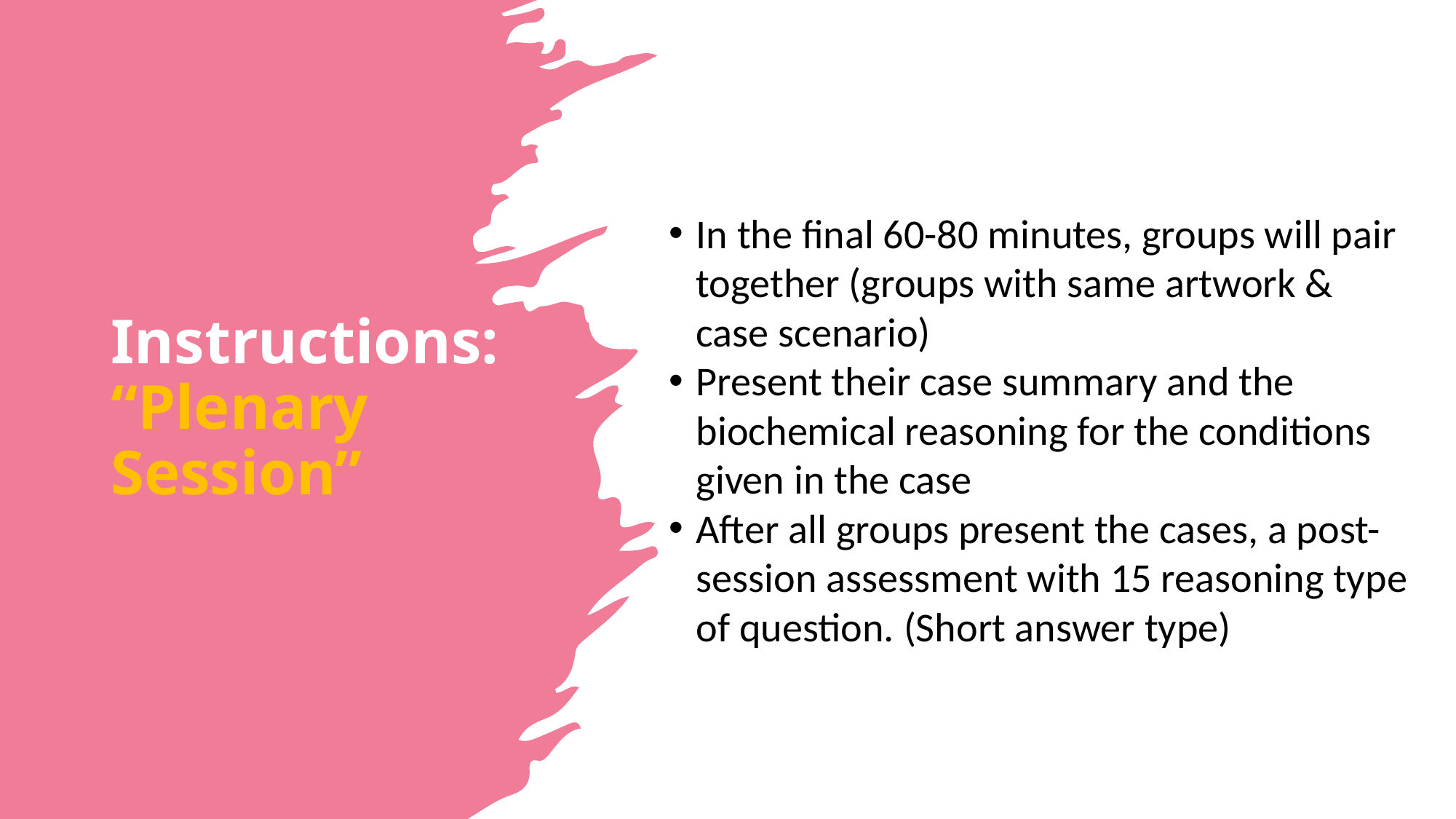

In the final 60-80 minutes, groups will pair together (groups with same artwork & case scenario)
Present their case summary and the biochemical reasoning for the conditions given in the case
After all groups present the cases, a post-session assessment with 15 reasoning type of question. (Short answer type)
# Instructions: “Plenary Session”

## Slide 12
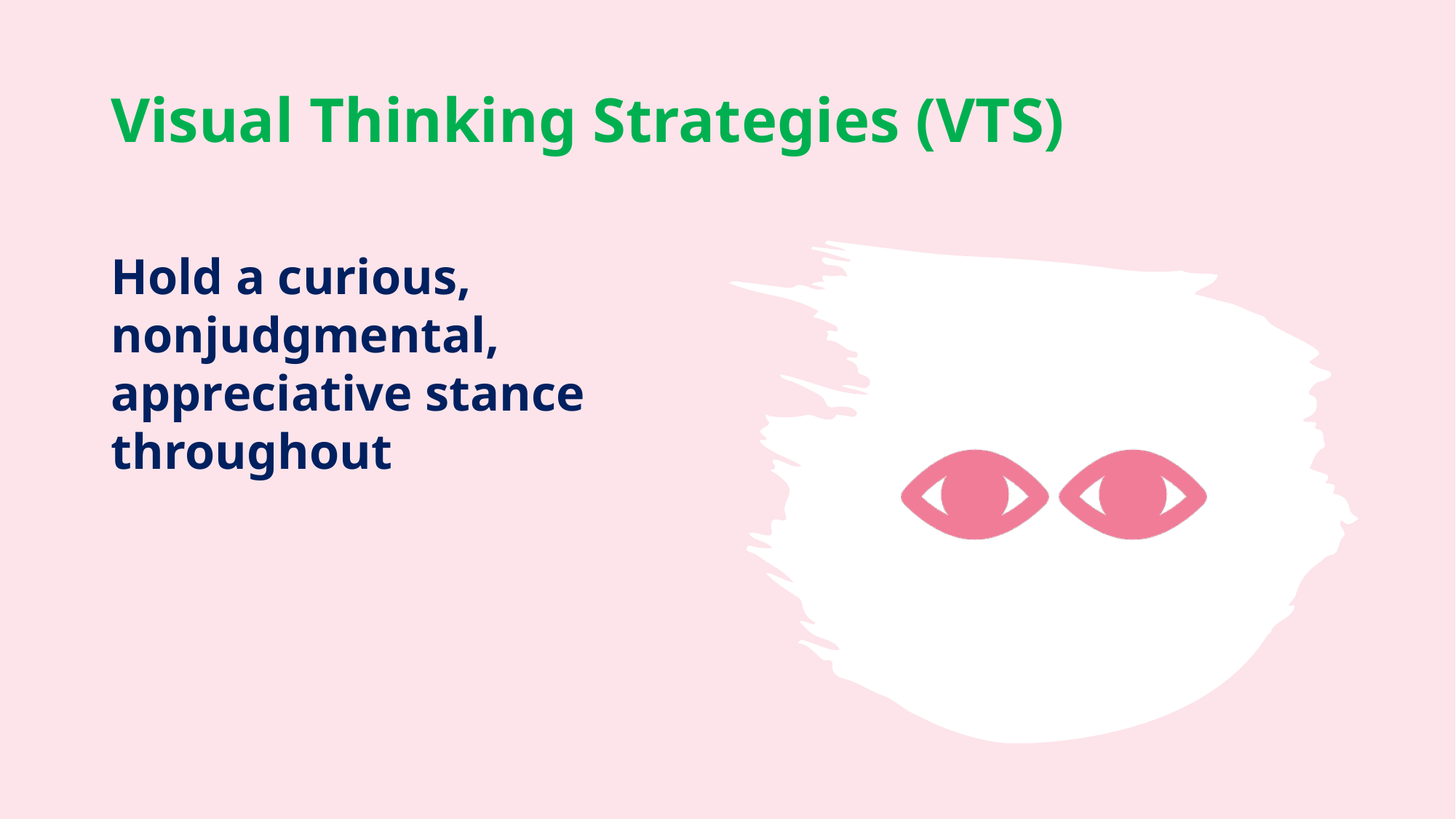

# Visual Thinking Strategies (VTS)
Hold a curious, nonjudgmental, appreciative stance throughout

## Slide 13
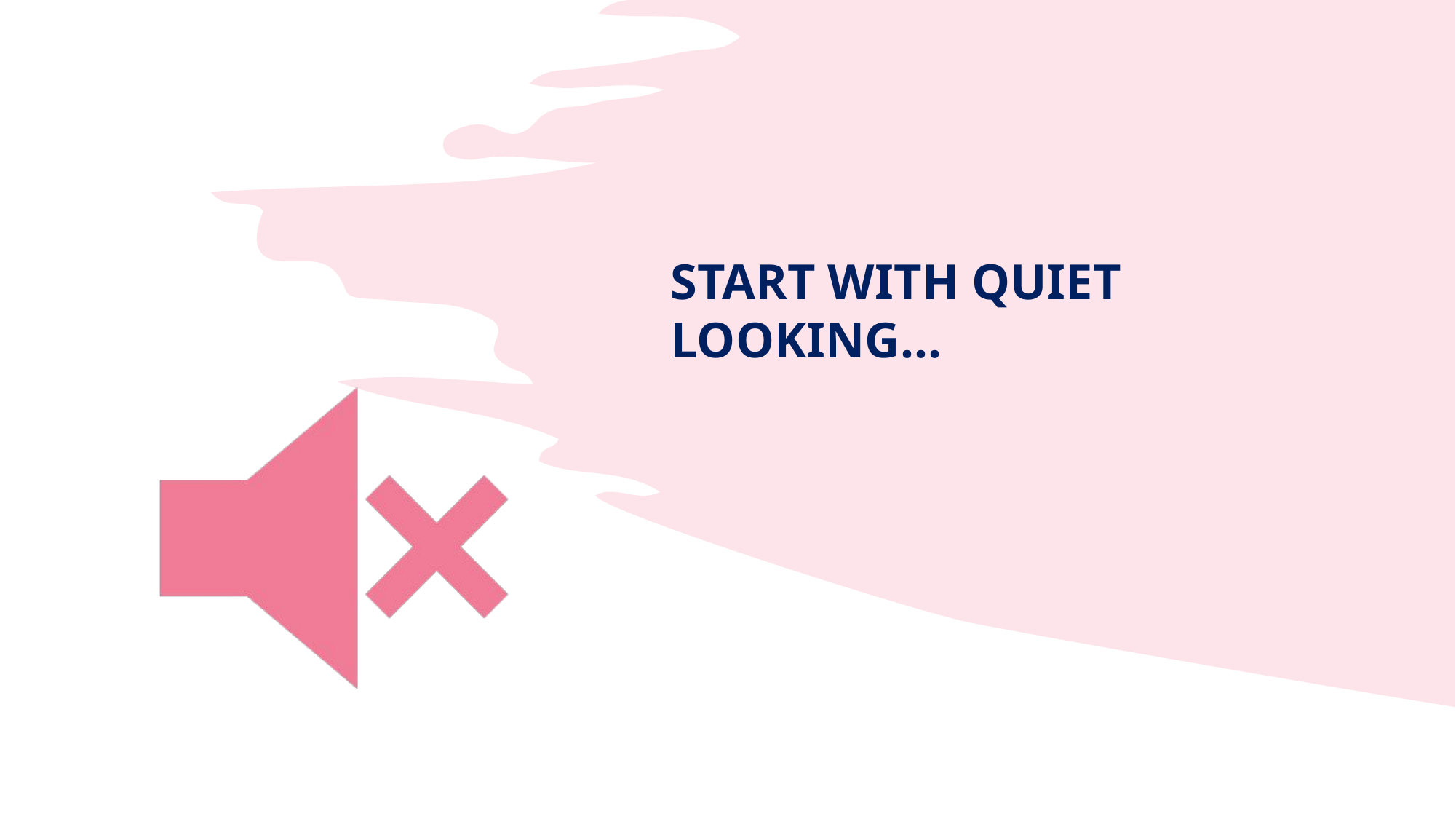

Start with quiet looking…

## Slide 14
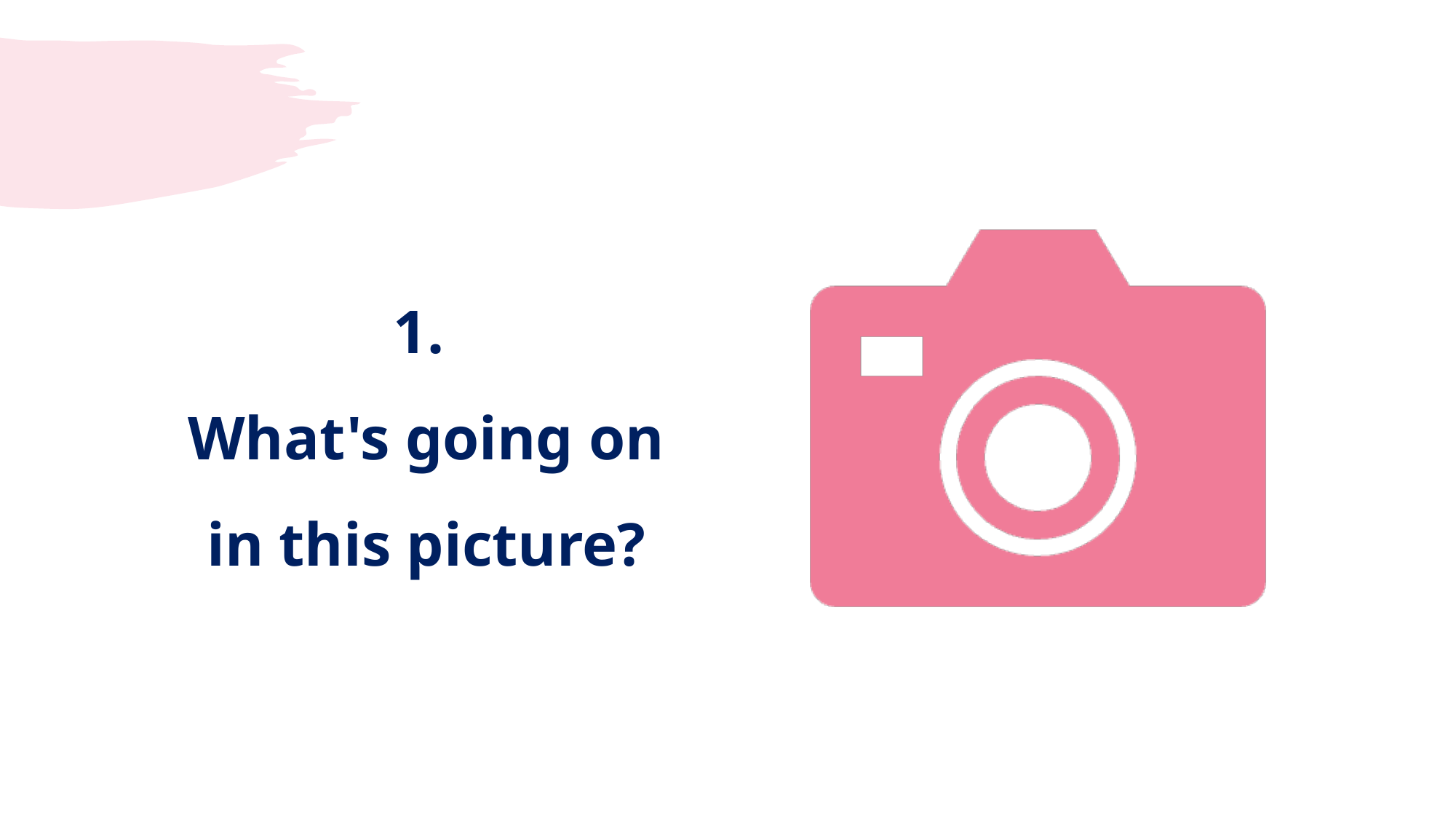

# 1. What's going on in this picture?

## Slide 15
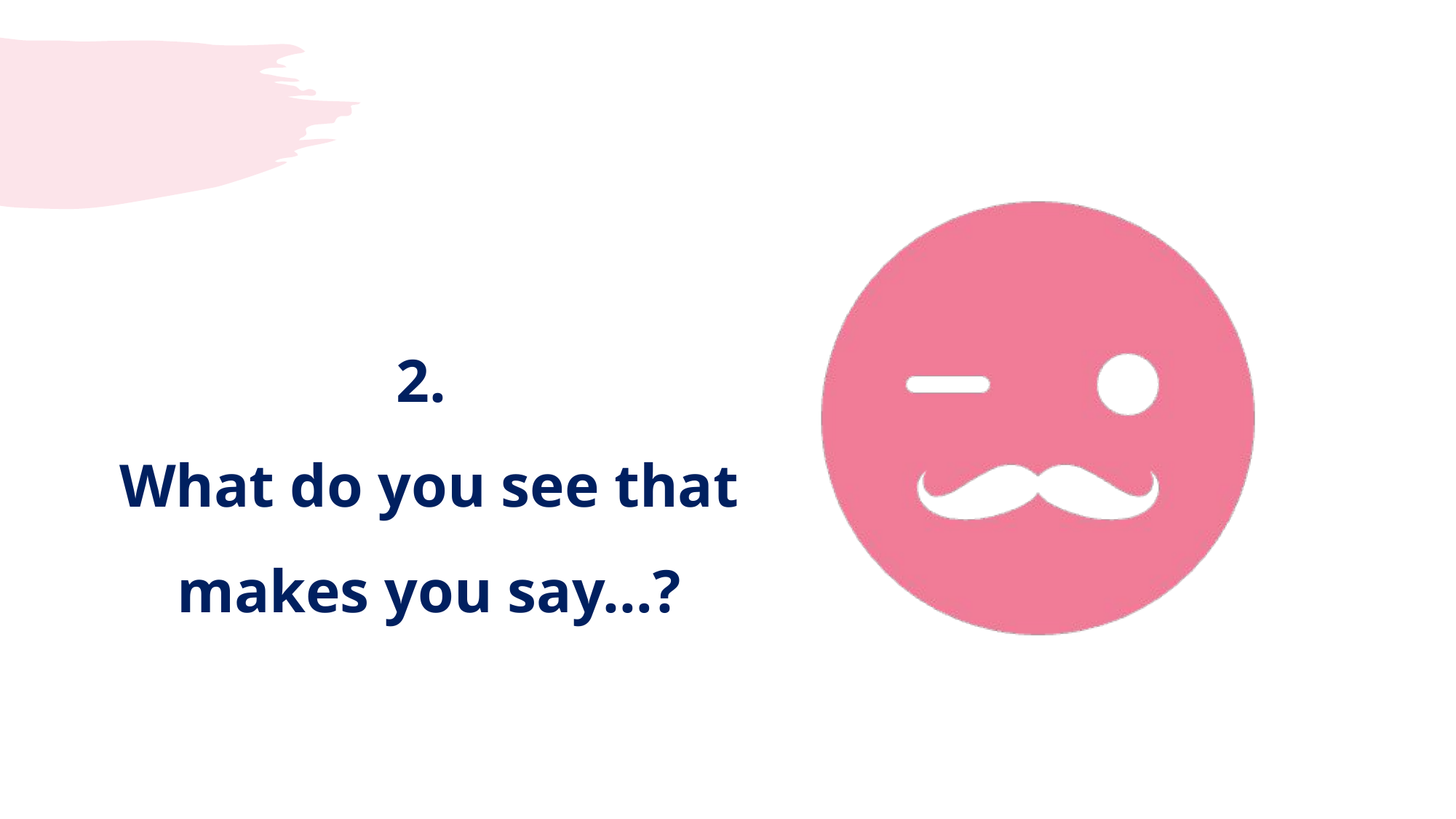

# 2. What do you see that makes you say…?

## Slide 16
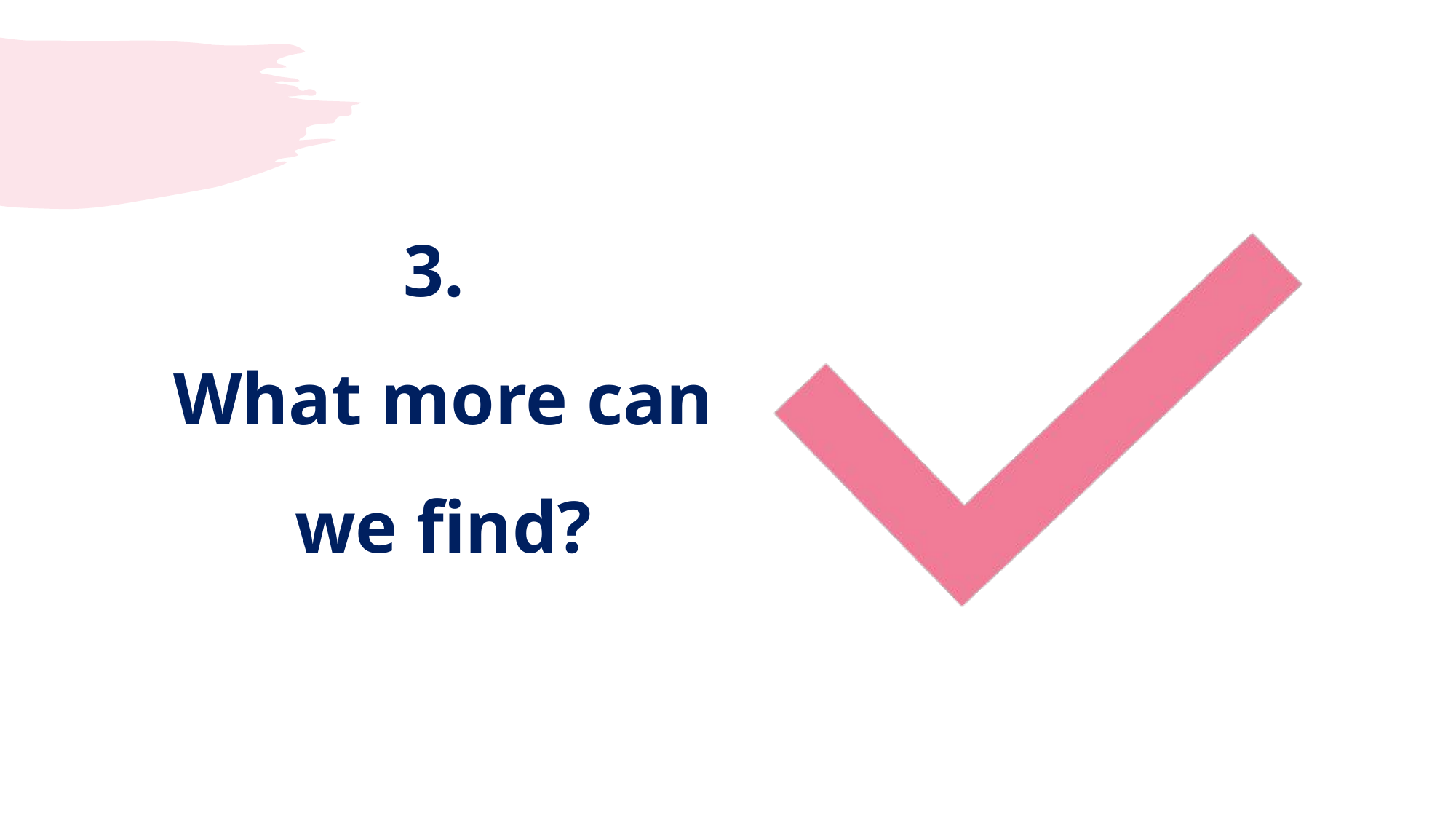

# 3. What more can we find?

## Slide 17
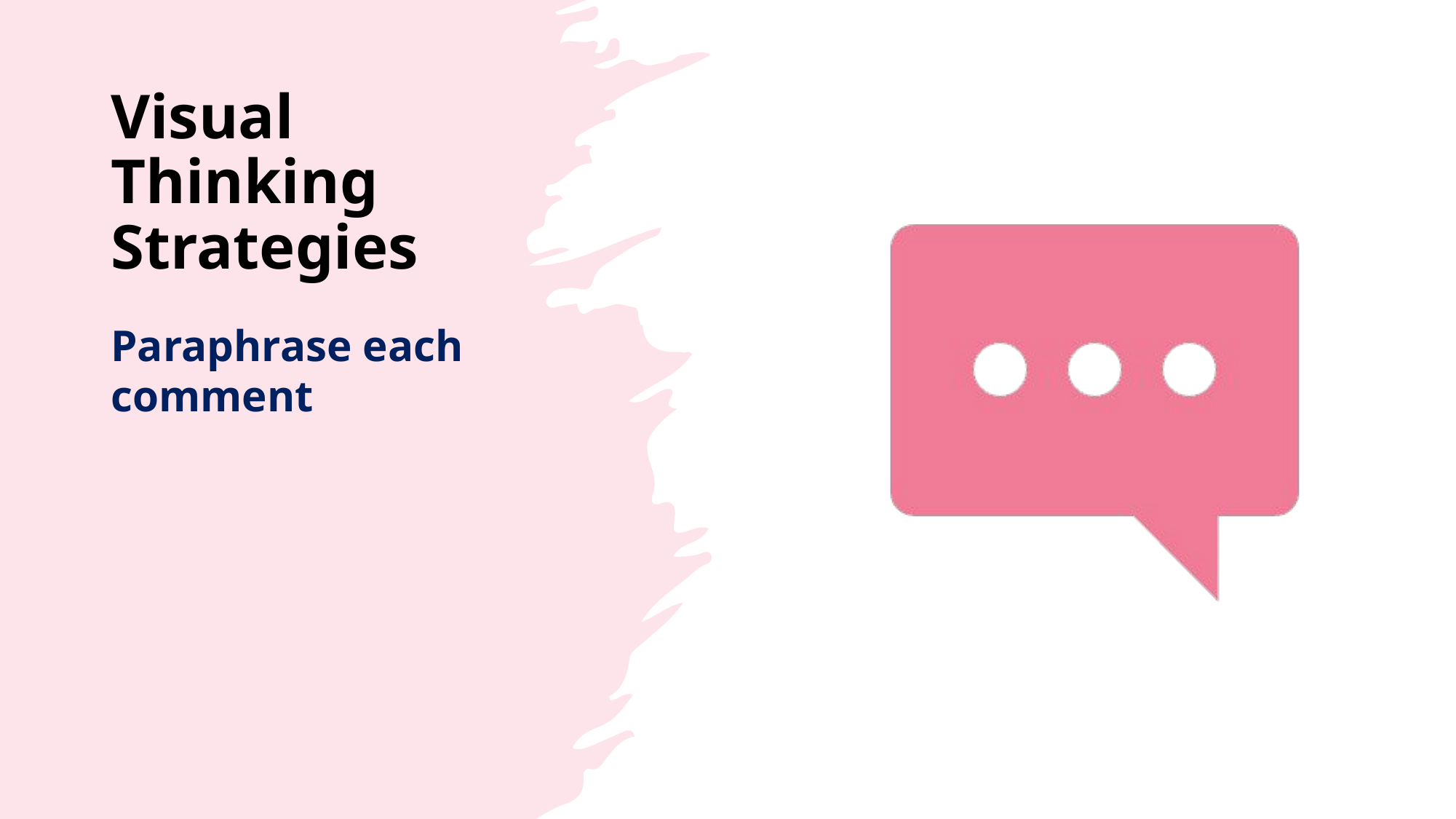

# Visual Thinking Strategies
Paraphrase each comment

## Slide 18
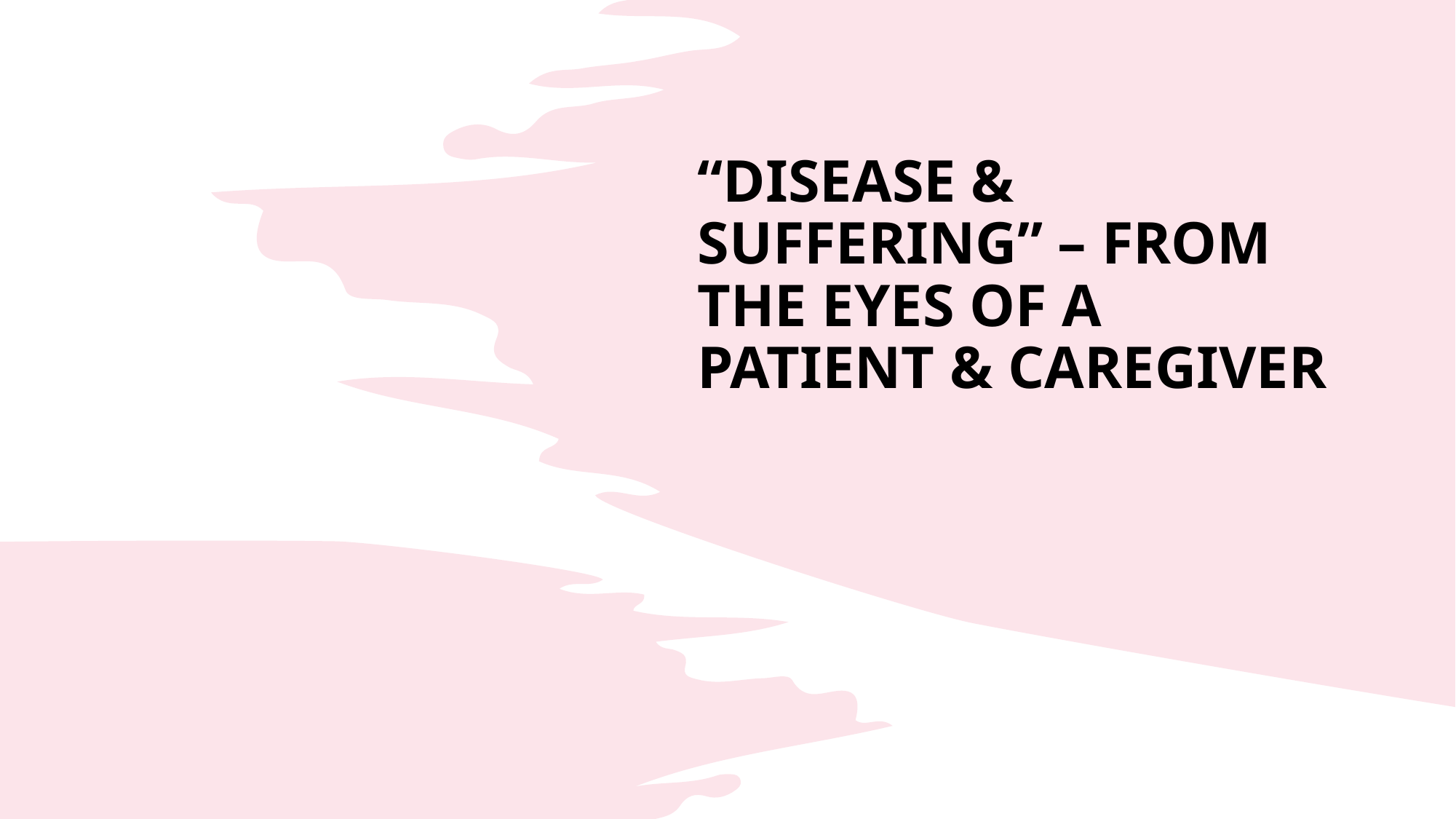

# “DISEASE & SUFFERING” – FROM THE EYES OF A PATIENT & CAREGIVER

## Slide 19
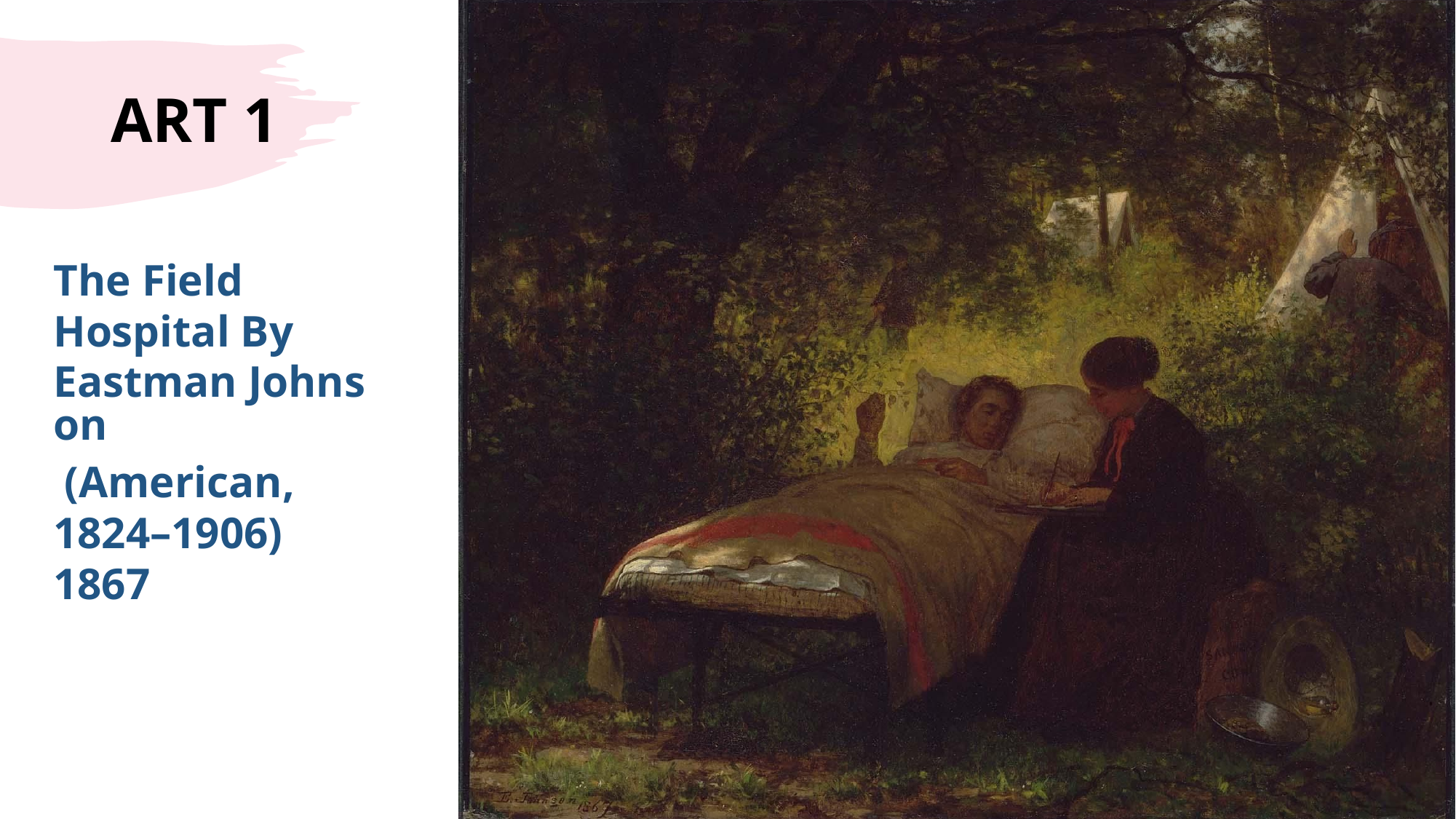

# ART 1
The Field Hospital By Eastman Johnson (American, 1824–1906) 1867

## Slide 20
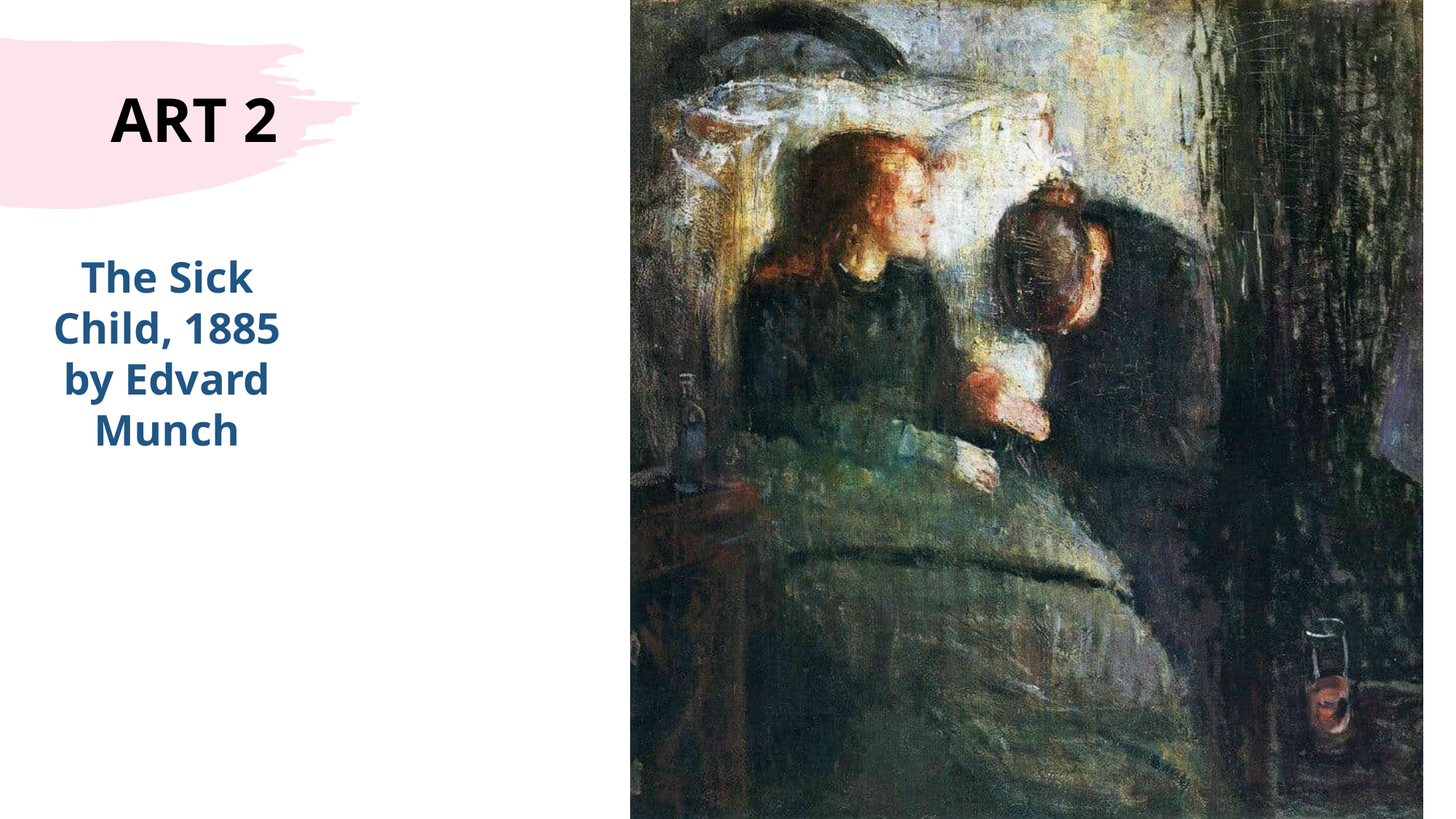

# ART 2
The Sick Child, 1885 by Edvard Munch

## Slide 21
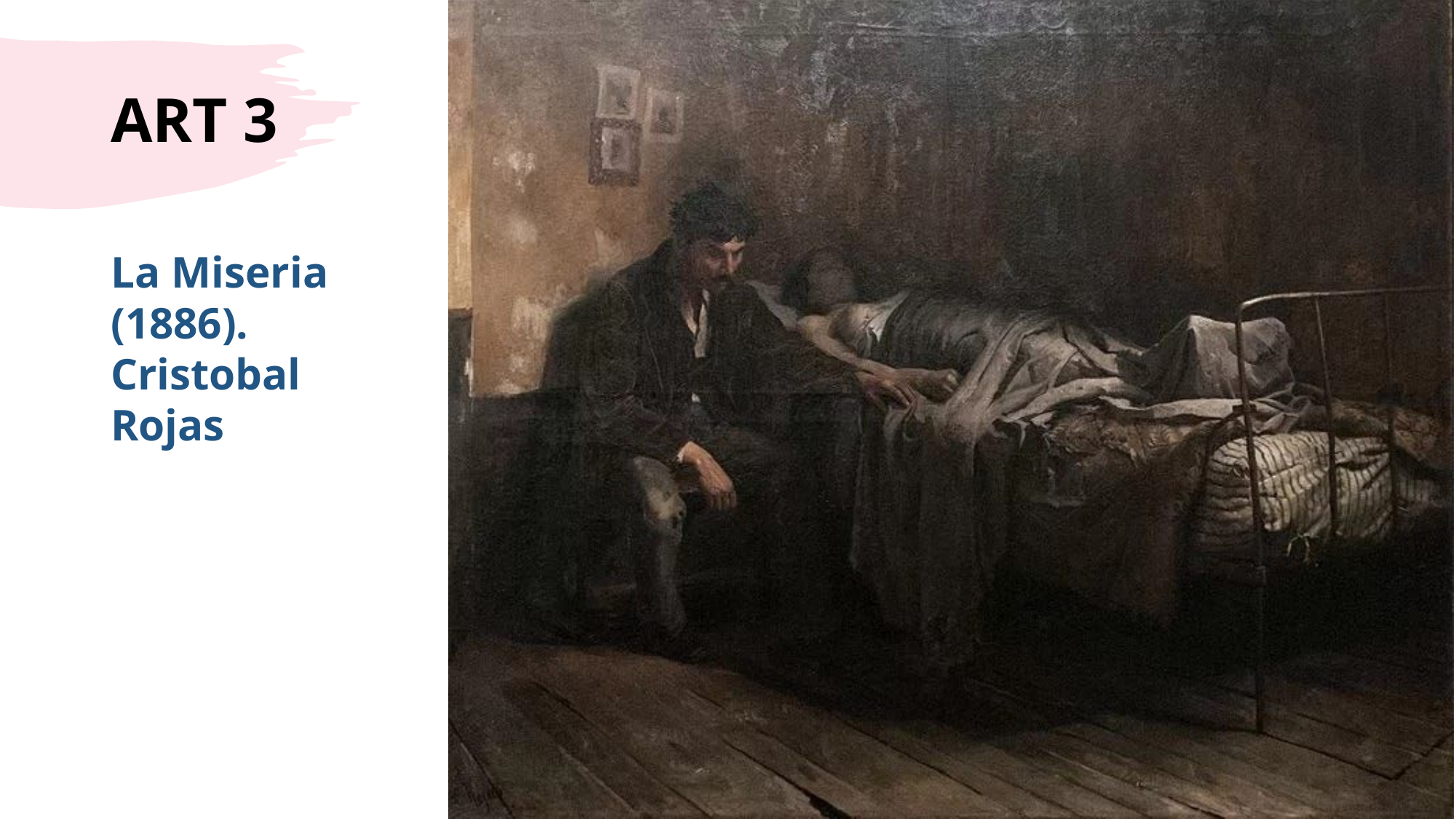

# ART 3
La Miseria (1886). Cristobal Rojas

## Slide 22
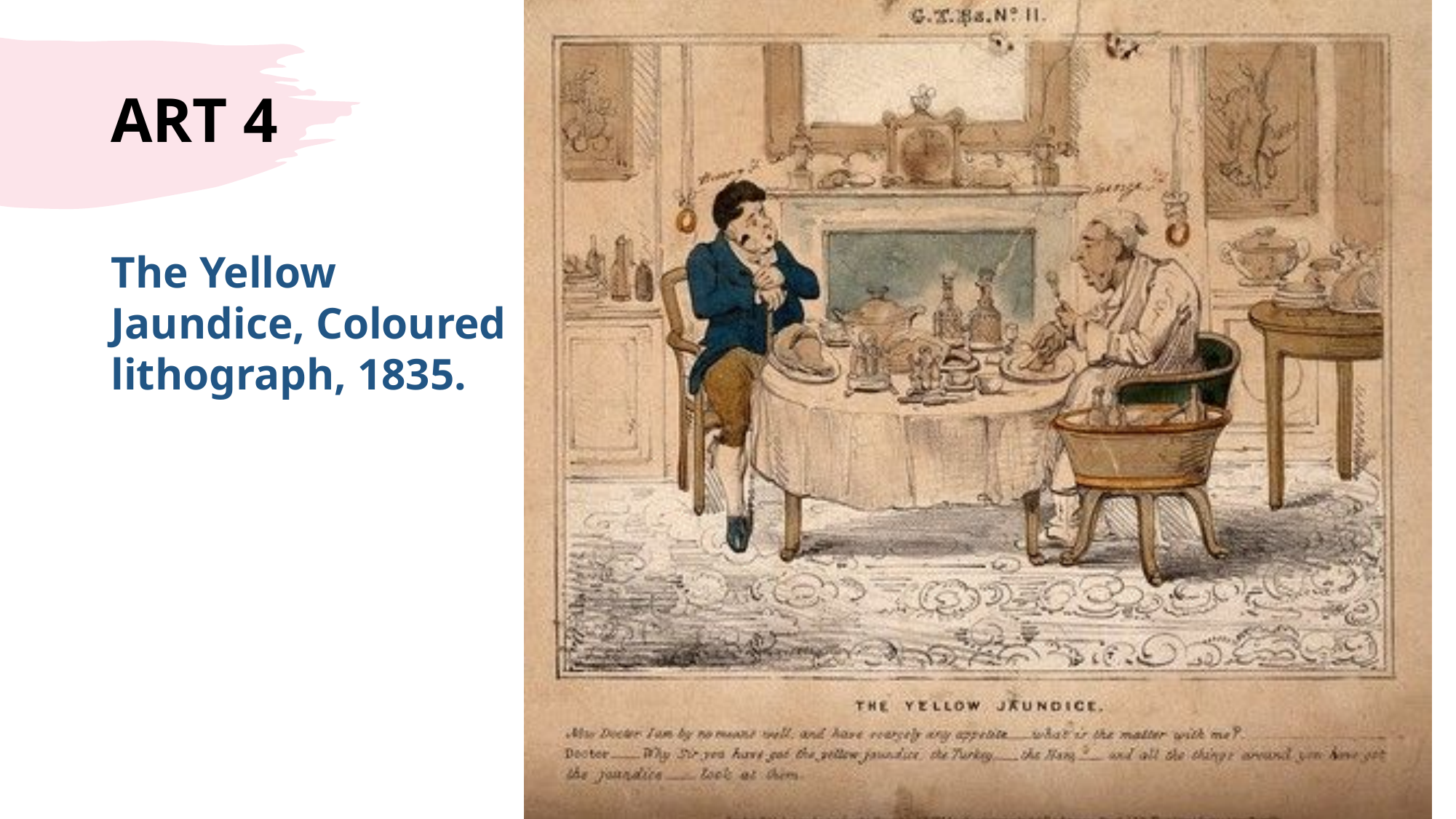

# ART 4
The Yellow Jaundice, Coloured lithograph, 1835.

## Slide 23
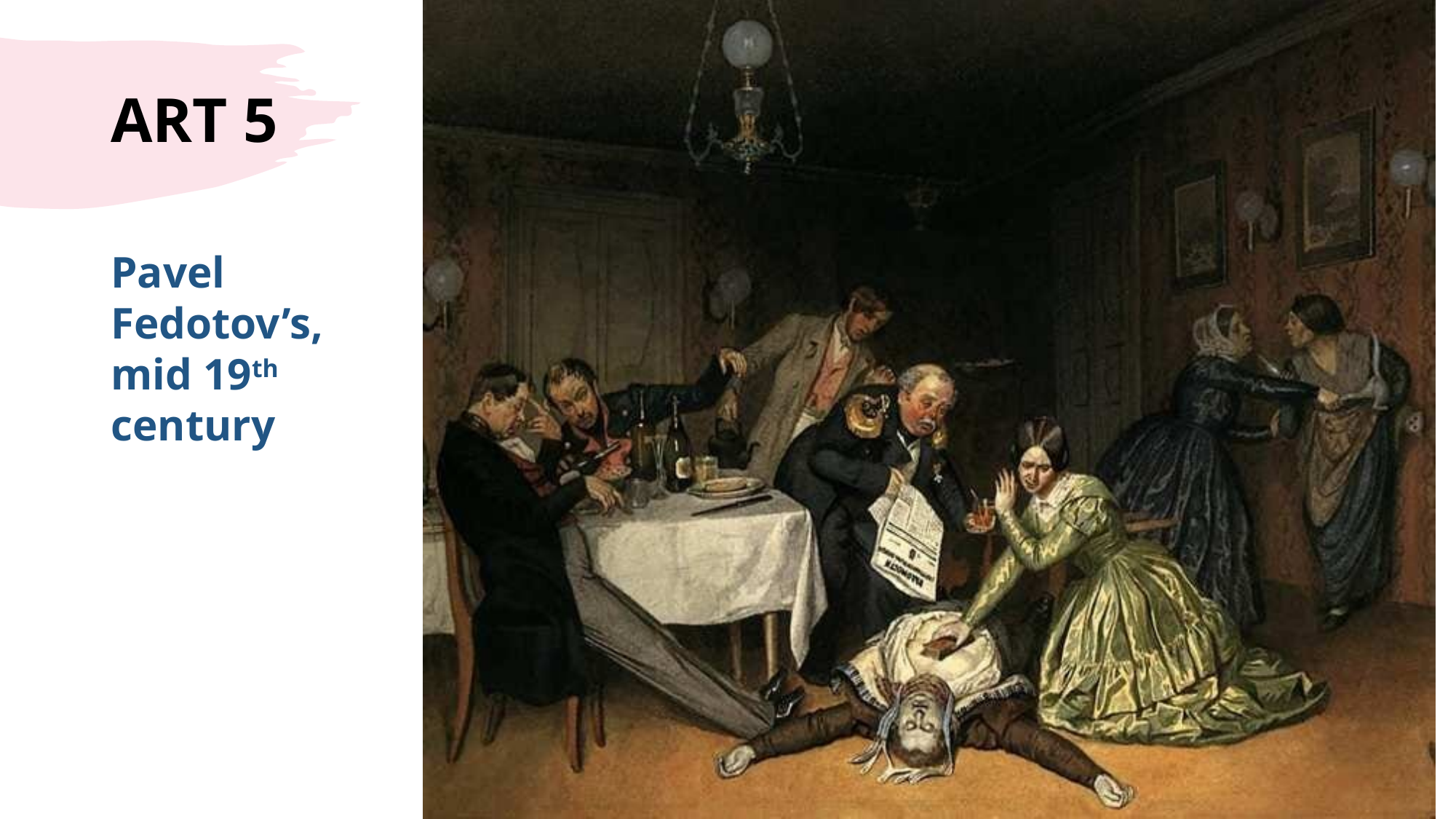

# ART 5
Pavel Fedotov’s, mid 19th century

## Slide 24
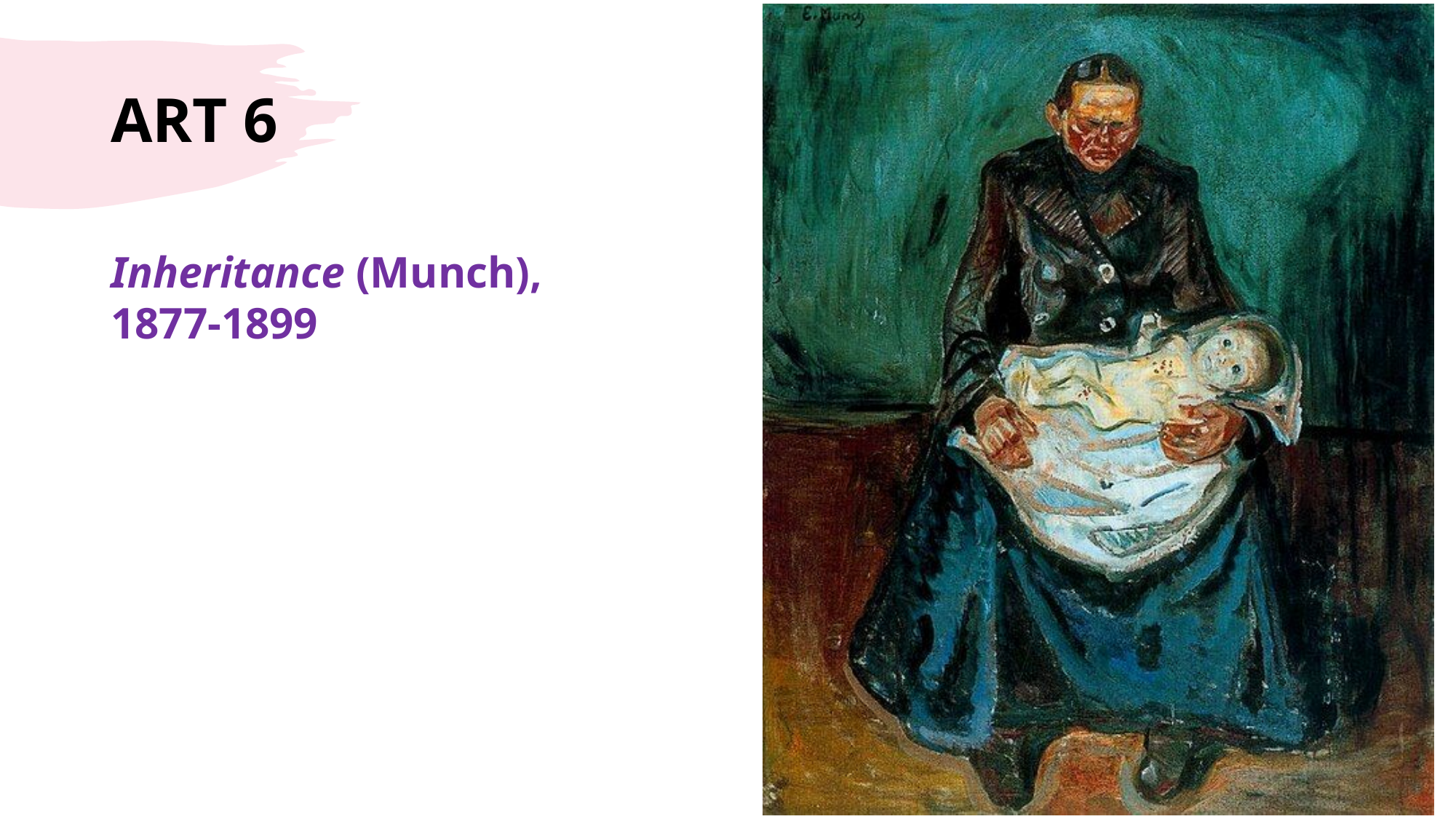

# ART 6
Inheritance (Munch), 1877-1899

## Slide 25
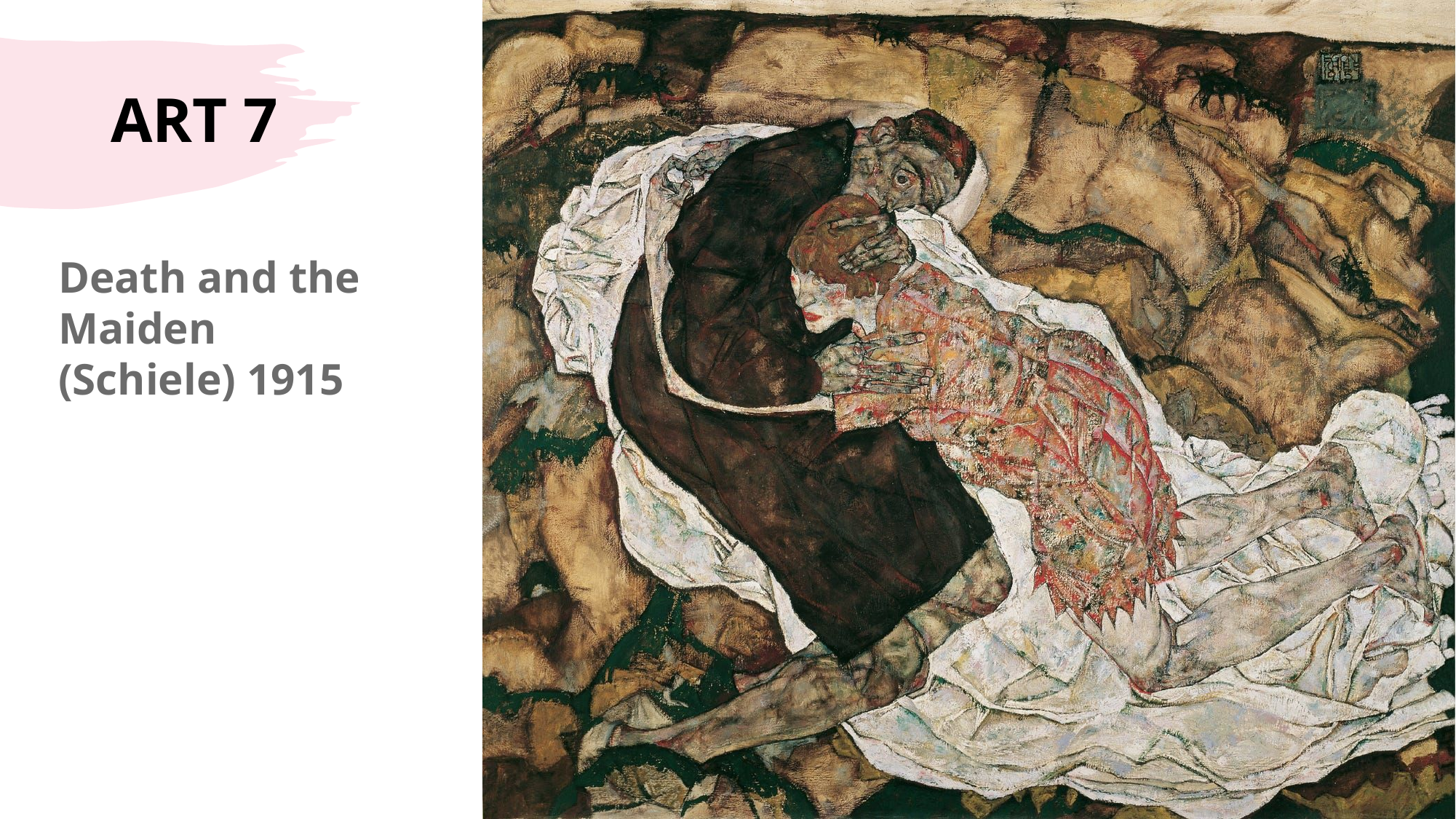

# ART 7
Death and the Maiden (Schiele) 1915

## Slide 26
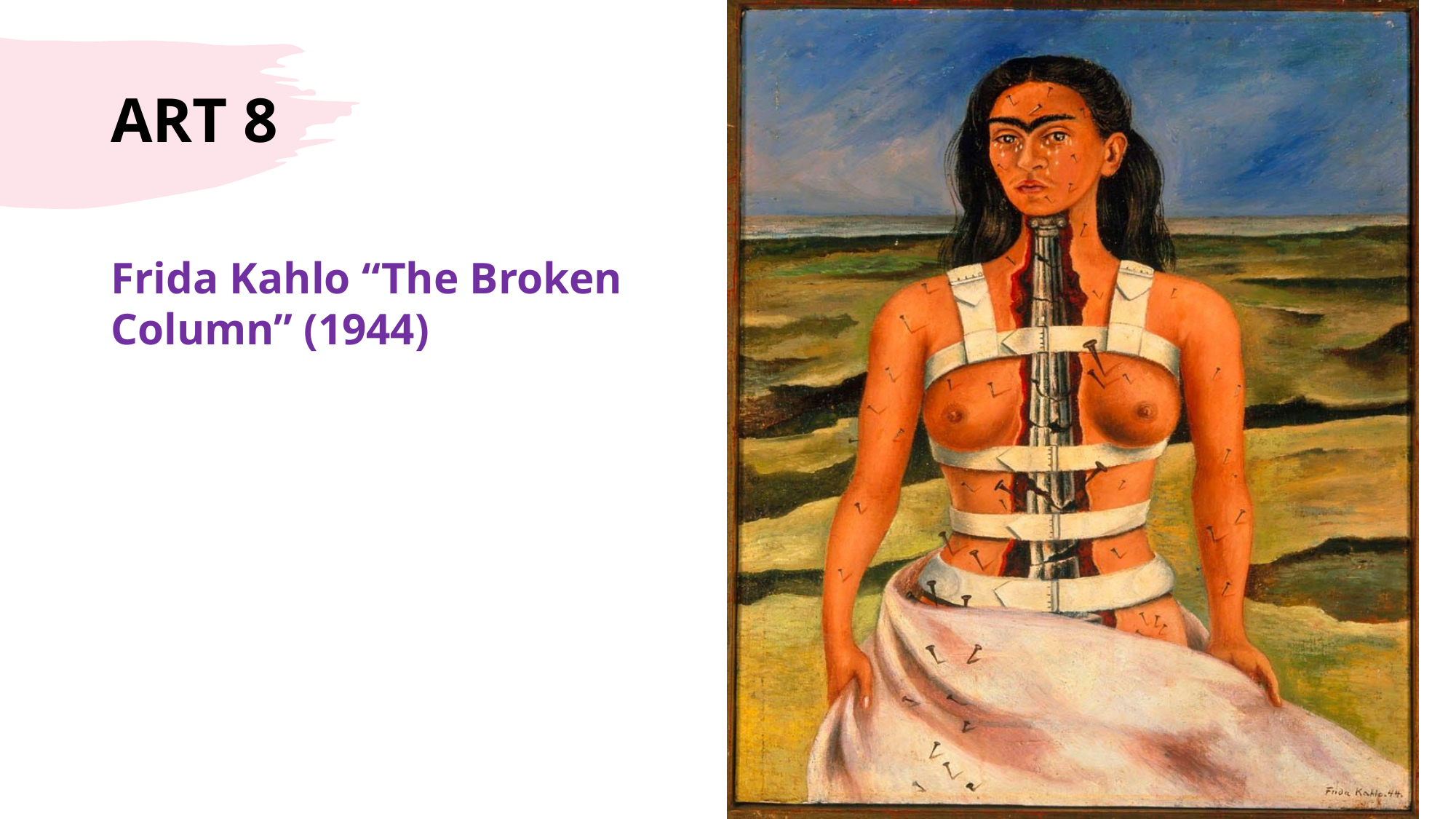

# ART 8
Frida Kahlo “The Broken Column” (1944)

## Slide 27
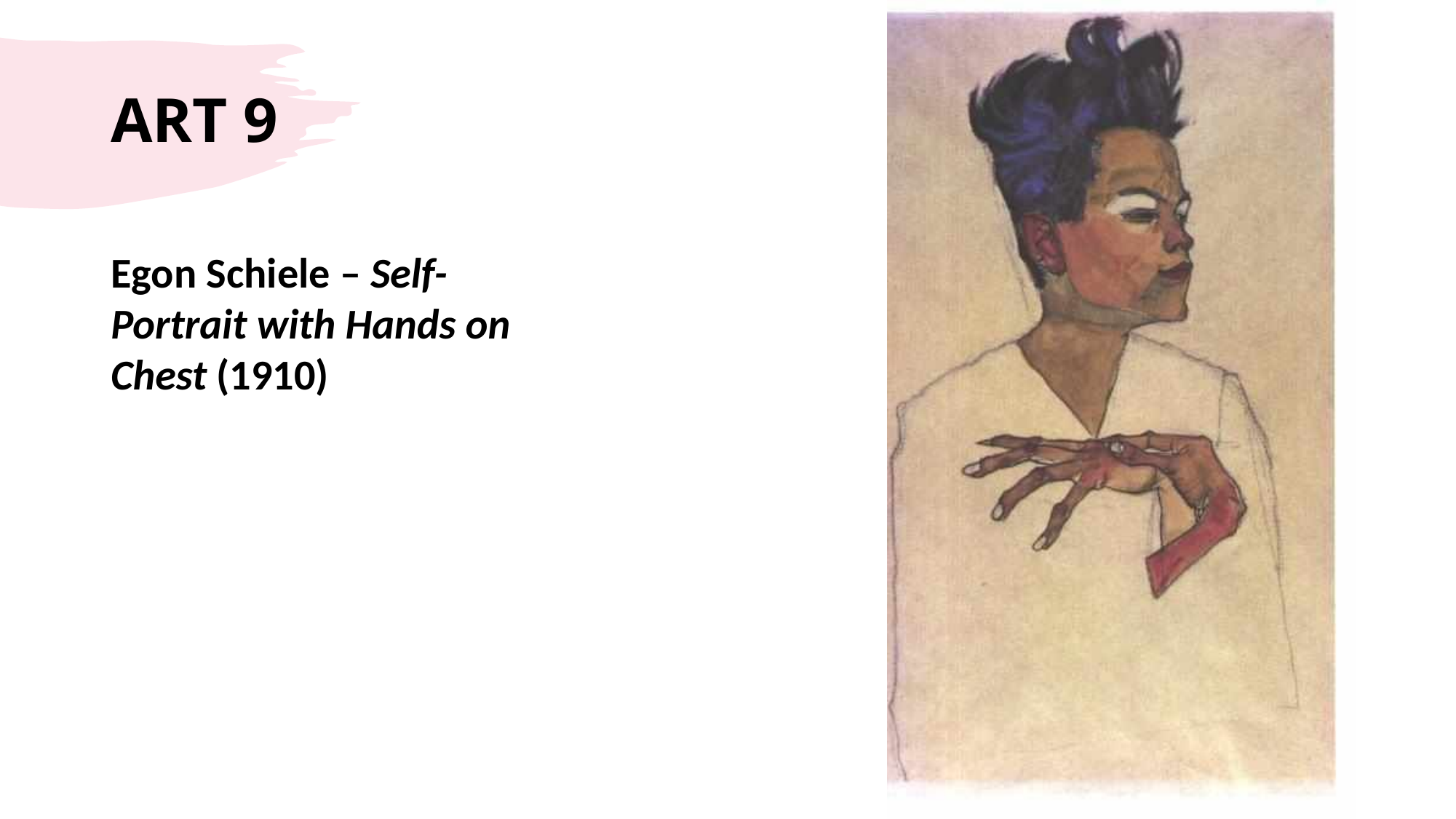

# ART 9
Egon Schiele – Self-Portrait with Hands on Chest (1910)

## Slide 28
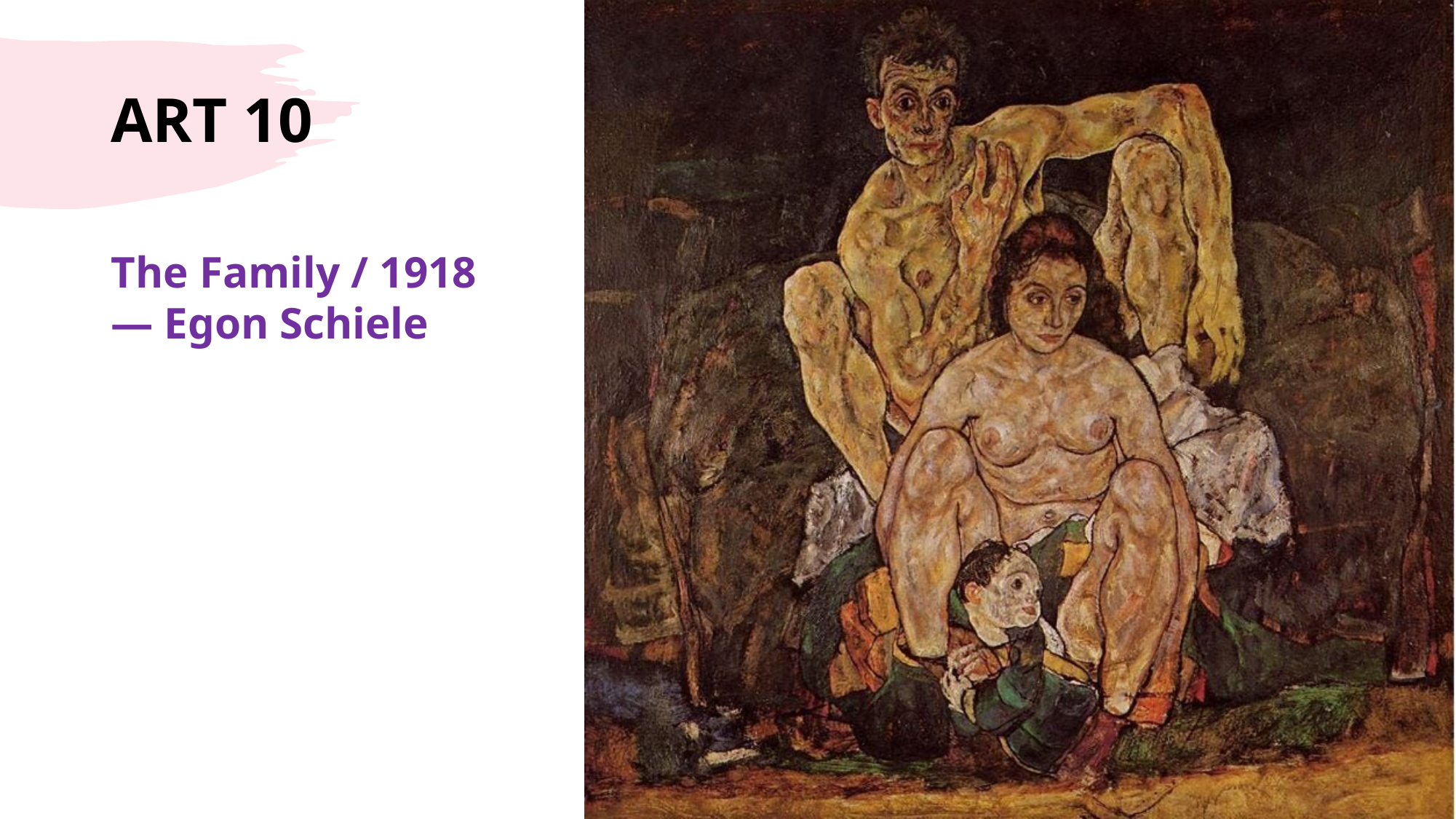

# ART 10
The Family / 1918 — Egon Schiele

## Slide 29
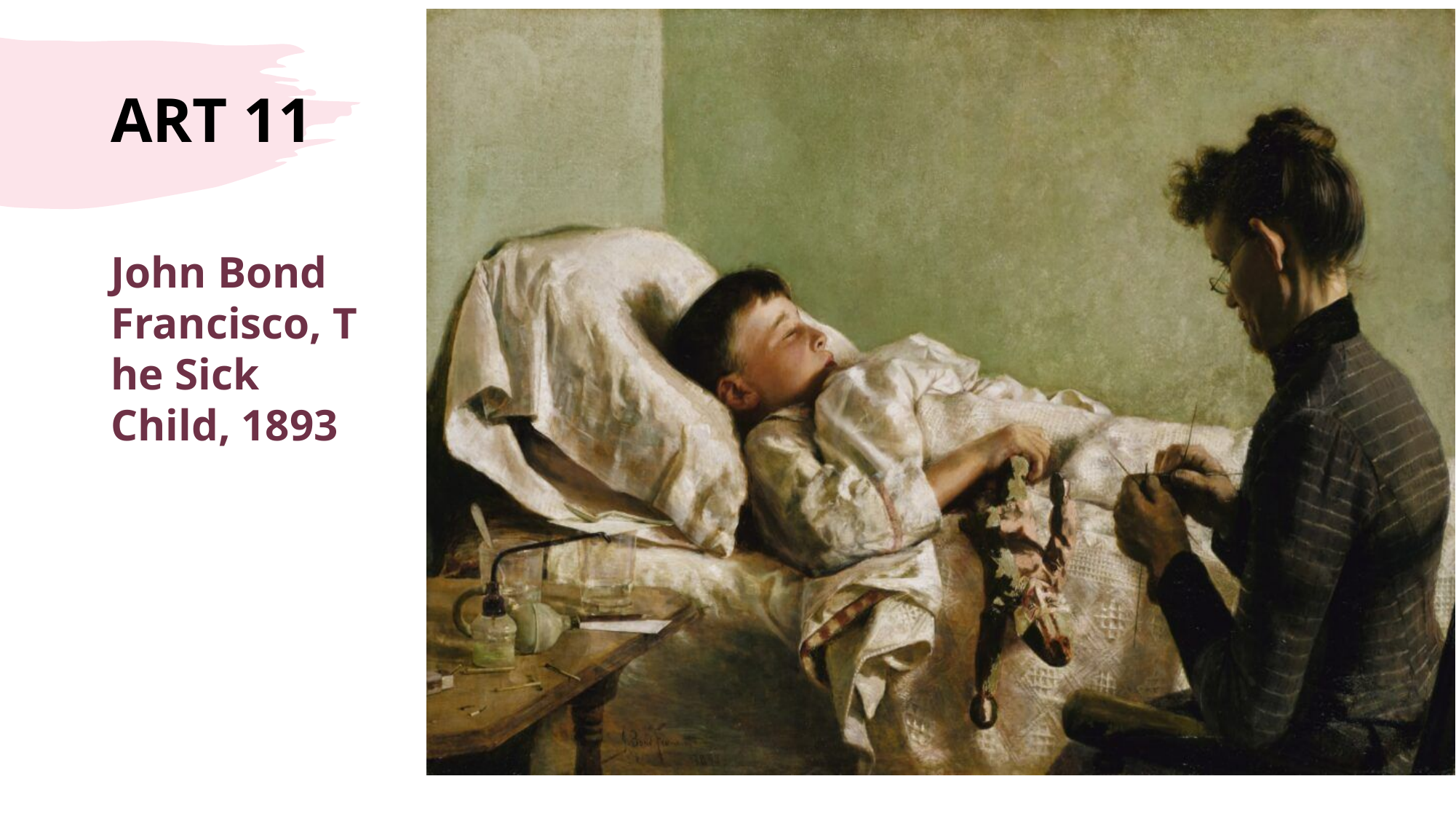

# ART 11
John Bond Francisco, The Sick Child, 1893

## Slide 30
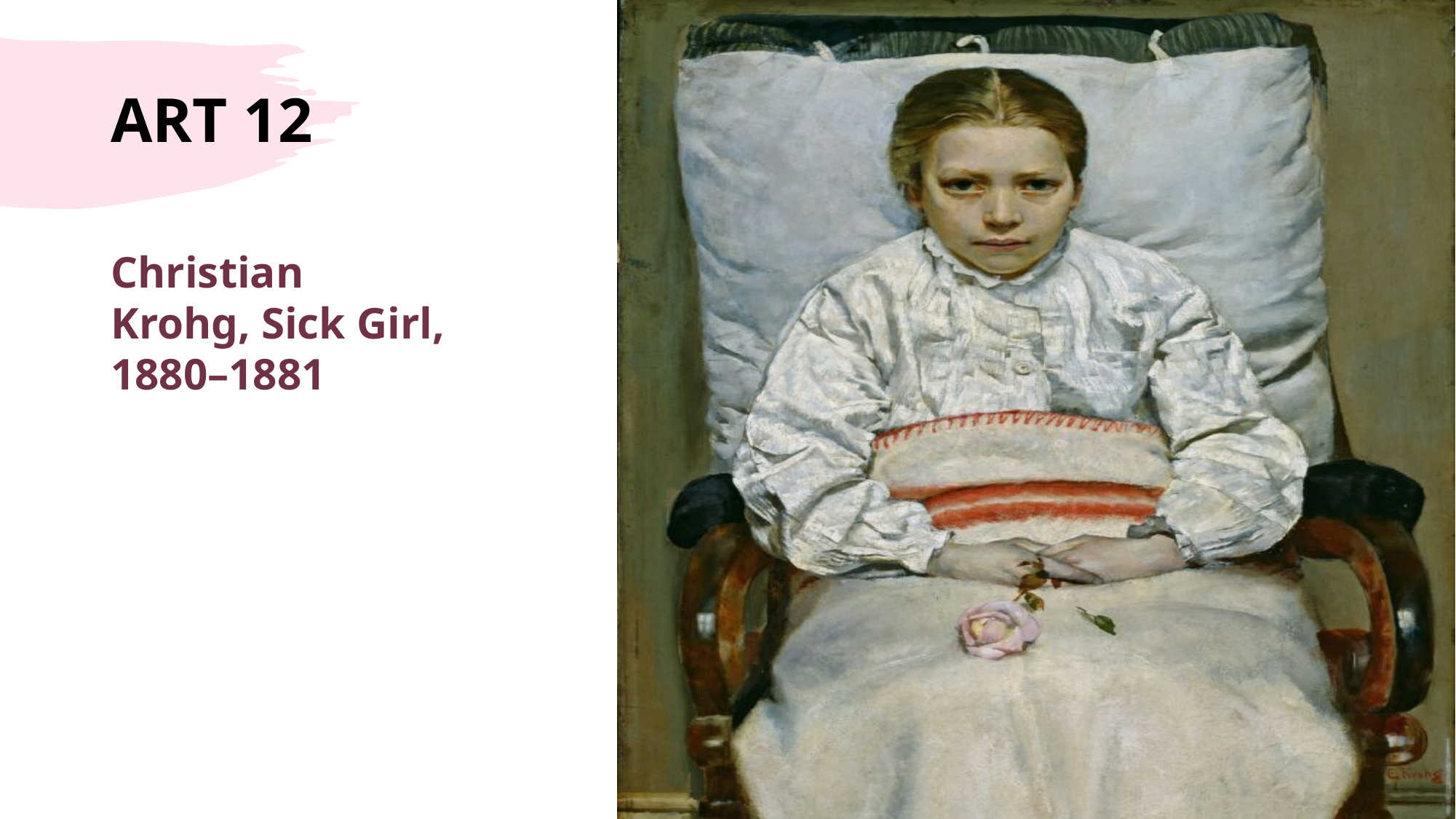

# ART 12
Christian Krohg, Sick Girl, 1880–1881

## Slide 31
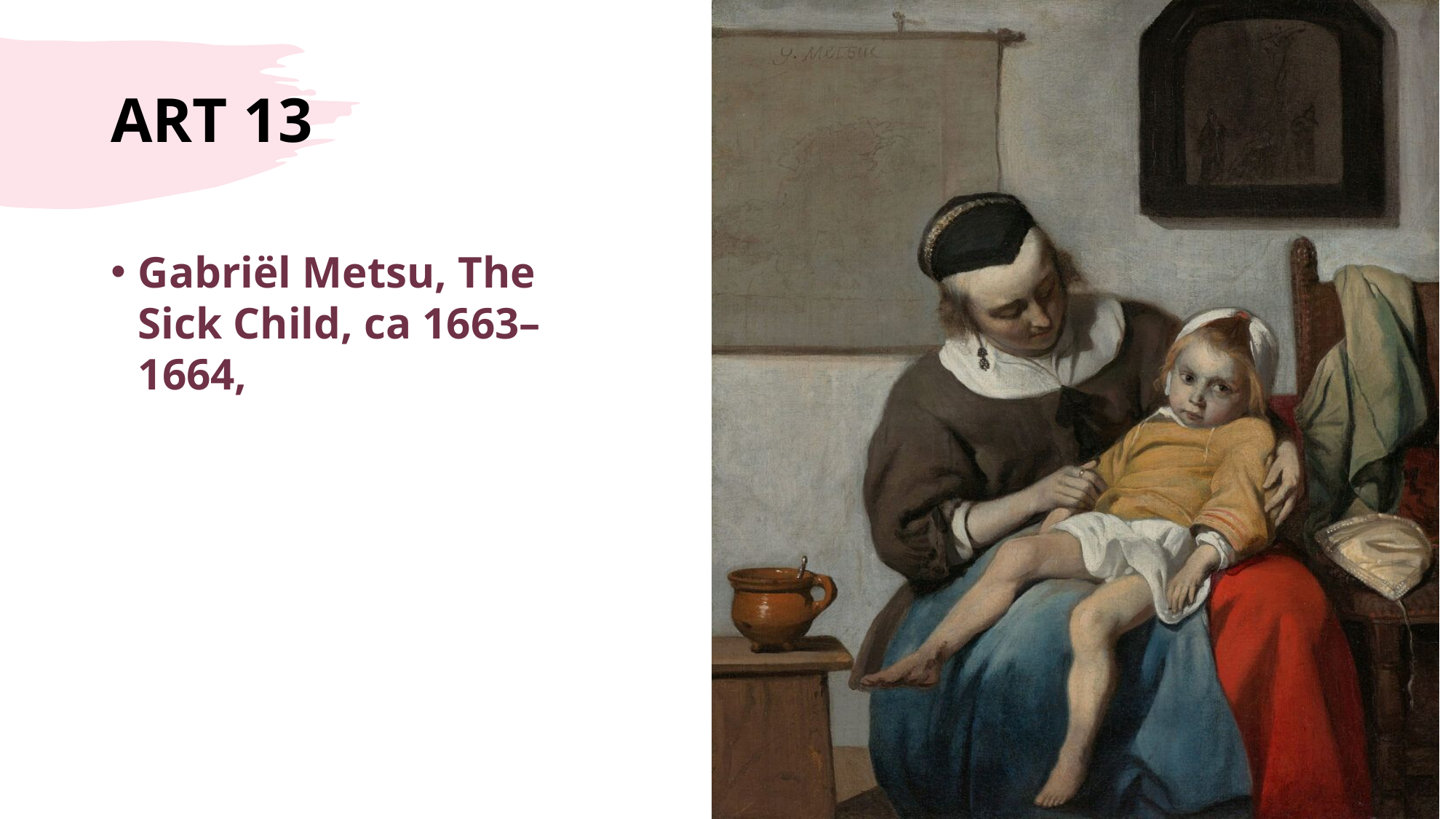

# ART 13
Gabriël Metsu, The Sick Child, ca 1663–1664,

## Slide 32
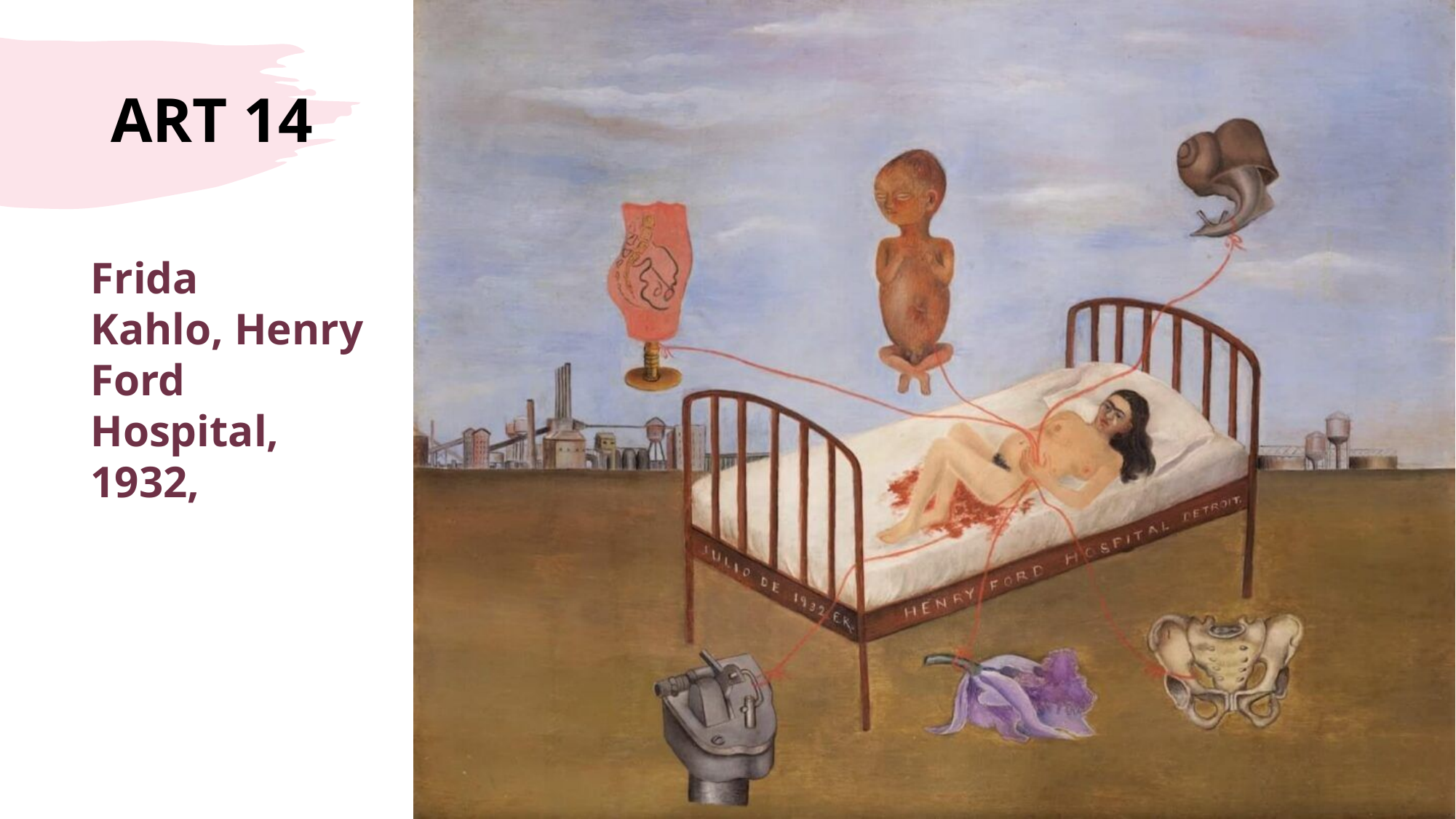

# ART 14
Frida Kahlo, Henry Ford Hospital, 1932,

## Slide 33
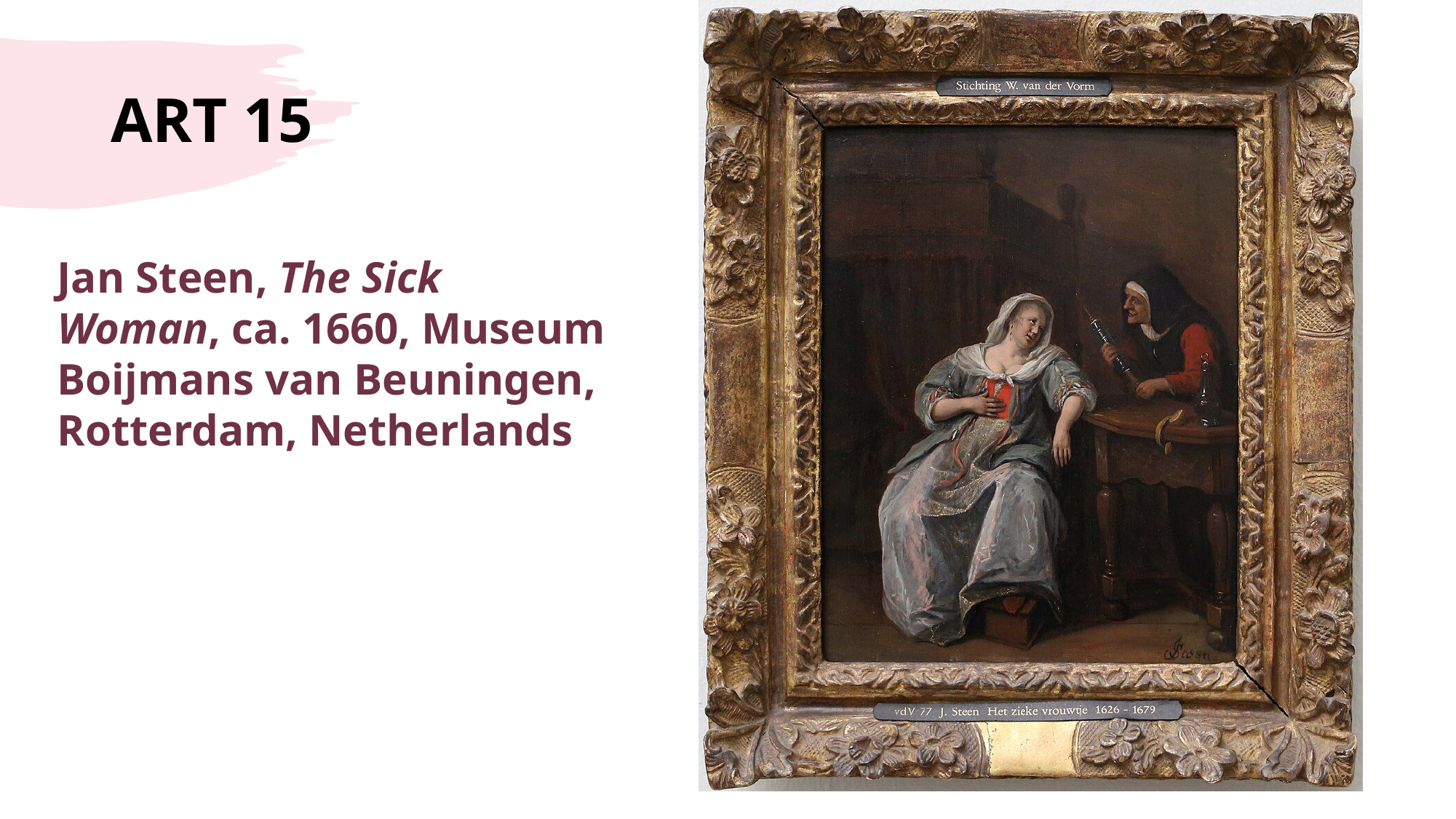

# ART 15
Jan Steen, The Sick Woman, ca. 1660, Museum Boijmans van Beuningen, Rotterdam, Netherlands

## Slide 34
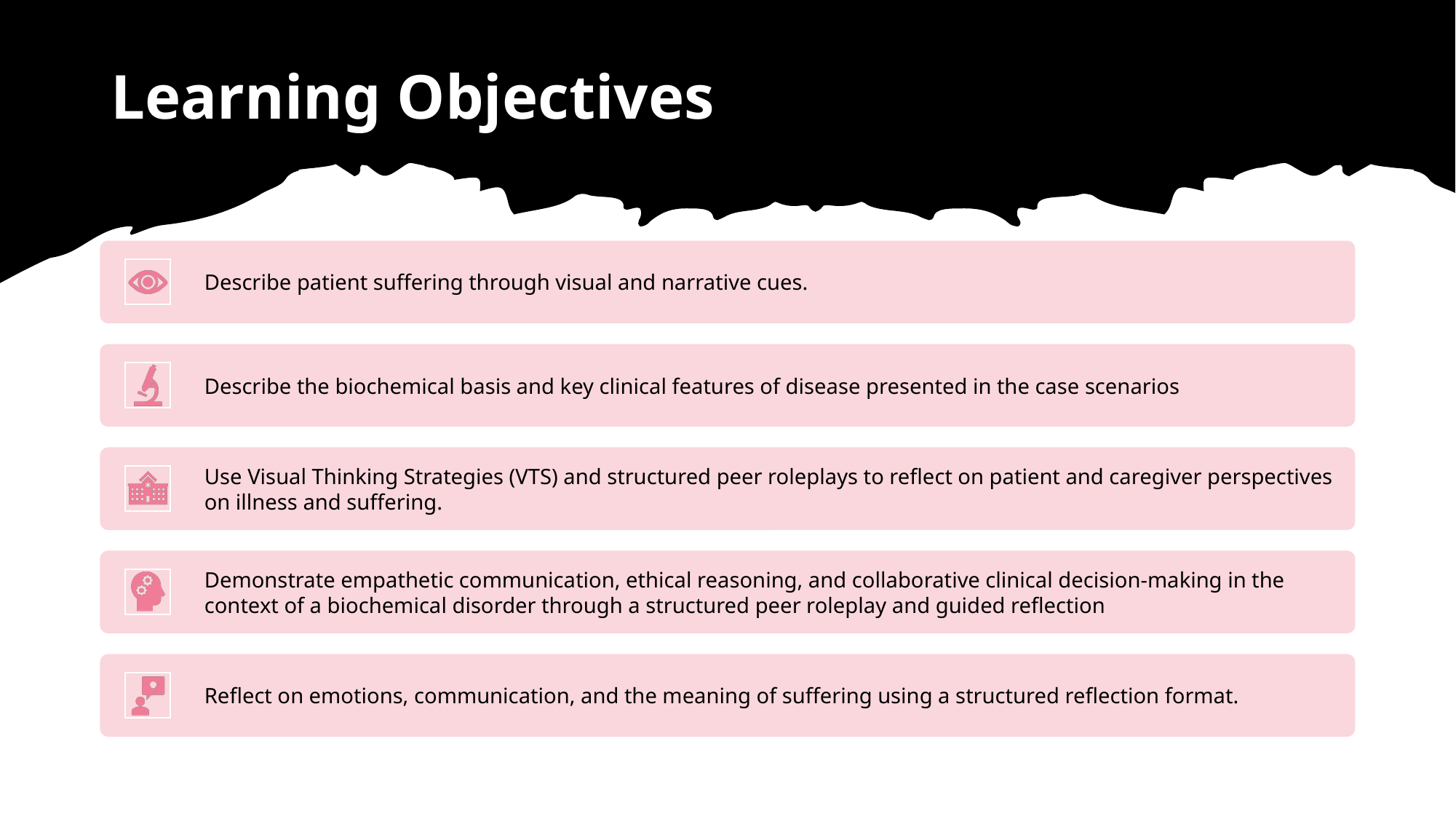

# Learning Objectives
By the end of this session, students will be able to

## Slide 35
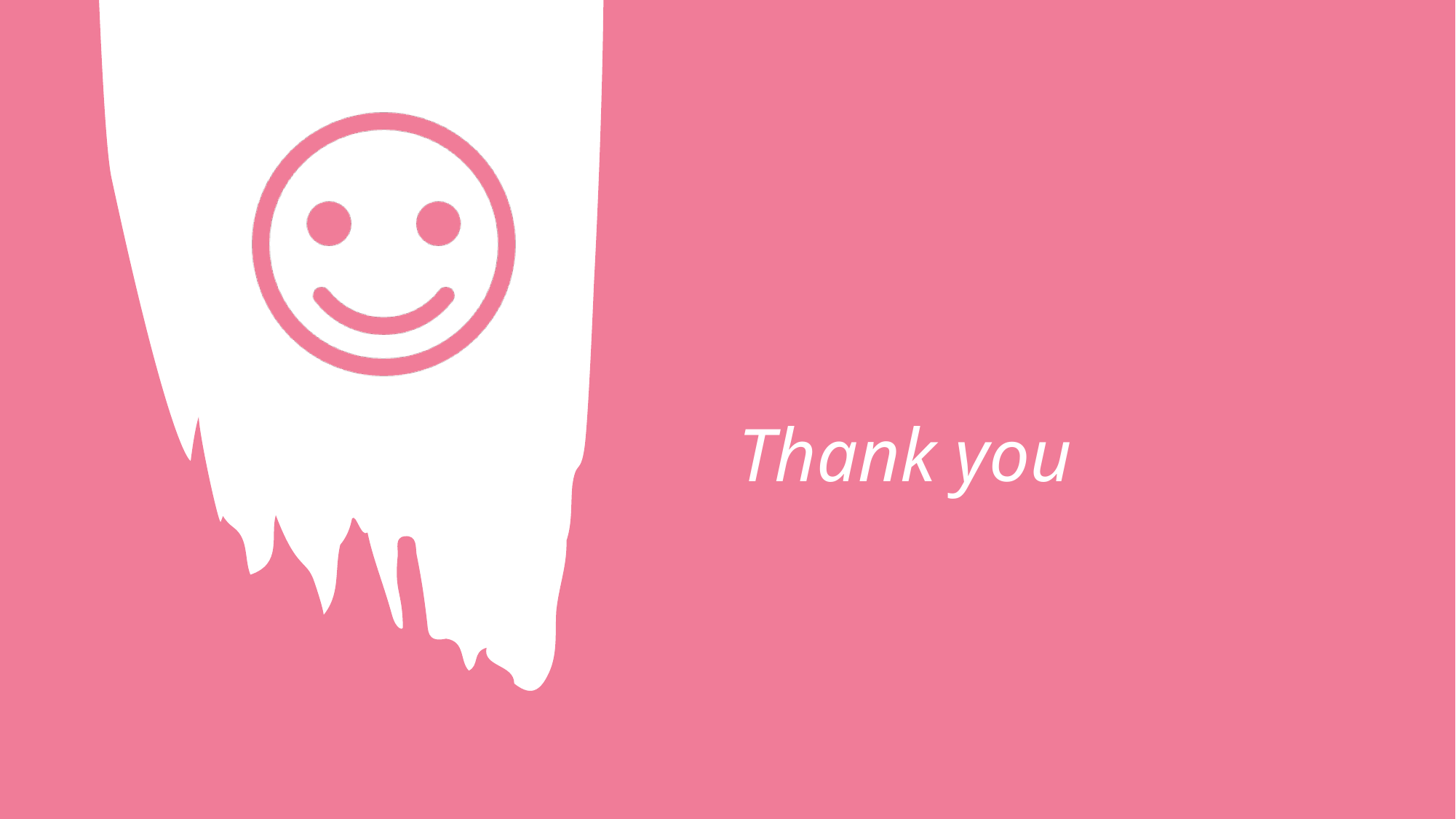

# Thank you
