# Supplementary material for: The Palette of Science and Emotions: Art-Based Learning With Structured Peer Role-Plays for Early Clinical Exposure in Biochemistry
Source: MedEdPORTAL. 2026 May 19;22:11601. doi: 10.15766/mep_2374-8265.11601 (PMC13183865; doi:10.15766/mep_2374-8265.11601)
Supplement: Supplementary file 1 — Faculty Orientation.pptxCurated Artworks.docxActivity Instructions.docxRole-Play Resources.docxFacilitator Guide.docxPersonal Reflection Questionnaire.docxEvaluation Questionnaire.docxSemistructured Interview Guide.docxPostsession Assessment.docxConfidence Questionnaire.docx [file mep_2374-8265.11601-s001.zip › I. Postsession Assessment.docx]

**Post-Session Assessment**

**(Distributed to students as print-out and collected after the test)**

**Note:** Educators who wish to implement this activity in their own settings can consider reshuffling the order of questions before distribution. This may help reduce recall bias related to the sequence of the workshop.

**Instructions to Students**

This assessment consists of 15 short-answer clinical reasoning questions. Each question requires explanation of the underlying biochemical mechanism responsible for the clinical presentation described.

Responses will be evaluated using a 0–2 analytic rubric:

- **2 marks** – Satisfactory clinical-biochemical understanding (clear mechanistic reasoning and appropriate correlation with clinical features)
- **1 mark** – Needs further conceptual strengthening (partial understanding, incomplete linkage between mechanism and presentation)
- **0 marks** – Inadequate clinical-biochemical reasoning (incorrect or absent explanation)

**Maximum score: 30 marks**

**Questions**

1. A 52-year-old man presents with confusion and asterixis. He is disoriented on examination. Serum ammonia is 120 µmol/L. Explain how excess ammonia affects astrocytes and neurotransmission in the brain.
2. A 10-year-old boy presents with recurrent bone pain, pallor, and fatigue. Hb is 7 g/dL, and peripheral smear shows sickled RBCs. Explain how the Glu to Val substitution in β-globin leads to HbS polymerization and sickling.
3. A 22-year-old man requiring transfusions since childhood presents with hepatosplenomegaly and bronze skin pigmentation. Ferritin is 1200 ng/mL. Explain how repeated transfusions result in excess iron deposition and tissue damage.
4. A 25-year-old woman presents with mild jaundice but is otherwise asymptomatic. Total bilirubin is 2.5 mg/dL (unconjugated). Explain the role of UDP-glucuronyl transferase in bilirubin conjugation.
5. A 48-year-old man with chronic alcohol use presents with confusion, ascites, and flapping tremor. Ammonia is 95 µmol/L. Explain how impaired hepatic urea cycle activity leads to ammonia accumulation.
6. A 6-month-old infant presents with developmental delay, hypopigmentation, and a musty odor. Phenylalanine is 20 mg/dL. Explain how phenylalanine accumulation interferes with brain development.
7. A 14-year-old girl with hereditary spherocytosis presents with pallor and fatigue after a recent viral illness. Hb is 5 g/dL with low reticulocyte count. Explain how bone marrow suppression leads to reduced erythropoiesis.
8. A 35-year-old woman on long-term corticosteroids presents with back pain and vertebral fractures. T-score is −2.8. Explain how glucocorticoids inhibit osteoblast activity and reduce bone formation.
9. An 8-year-old boy presents with recurrent fractures and blue sclera. Explain how defective type I collagen weakens the bone matrix.
10. A 30-year-old woman presents with severe abdominal pain, anxiety, and dark-colored urine during stress. Urine porphobilinogen is elevated. Explain how accumulation of heme precursors leads to neurovisceral symptoms.
11. A 12-year-old boy presents with marfanoid habitus and downward lens dislocation. Homocysteine is 25 µmol/L. Explain how elevated homocysteine causes endothelial injury and thrombosis.
12. An 18-year-old woman presents with generalized hypopigmentation, photophobia, and reduced visual acuity. Explain how tyrosinase deficiency impairs melanin synthesis.
13. A 2-year-old child presents with developmental delay, hypotonia, and characteristic facial features. Karyotype shows trisomy 21. Explain how gene dosage imbalance affects development.
14. A 28-year-old woman presents with petechiae, mucosal bleeding, and easy bruising. Platelet count is 20,000/µL. Explain how autoantibodies lead to platelet destruction.
15. A 55-year-old man presents with chest pain and diaphoresis. Troponin I is 2.5 ng/mL. Explain why troponin is released during myocardial injury.

**Key**

**1. Hyperammonemia / Urea Cycle Dysfunction**
Excess ammonia accumulates due to impaired urea cycle activity. It enters astrocytes and is converted to glutamine, causing osmotic swelling and altered neurotransmission, leading to encephalopathy.

**2. Sickle Cell Disease**
A Glu to Val substitution in the β-globin chain produces HbS, which polymerizes under low oxygen. This causes RBC sickling, leading to hemolysis and vaso-occlusion.

**3. Beta Thalassemia Major**
Reduced β-globin synthesis leads to excess α chains and ineffective erythropoiesis. Repeated transfusions cause iron accumulation, resulting in oxidative tissue damage.

**4. Gilbert Syndrome**
Reduced activity of UDP-glucuronyl transferase impairs bilirubin conjugation. This leads to mild unconjugated hyperbilirubinemia.

**5. Alcoholic Liver Disease / Hepatic Encephalopathy**
Liver dysfunction impairs ammonia detoxification via the urea cycle. Accumulated ammonia disrupts brain metabolism, causing encephalopathy.

**6. Phenylketonuria (PKU)**
Deficiency of phenylalanine hydroxylase leads to accumulation of phenylalanine. Toxic metabolites impair brain development and neurotransmitter synthesis.

**7. Hereditary Spherocytosis with Aplastic Crisis**
Spectrin/ankyrin defects cause fragile RBCs and chronic hemolysis. Viral infection suppresses bone marrow, reducing erythropoiesis and causing acute anemia.

**8. Secondary Osteoporosis**
Glucocorticoids inhibit osteoblast activity and increase bone resorption. Reduced estrogen further enhances bone loss, leading to decreased bone density.

**9. Osteogenesis Imperfecta**
Defective type I collagen weakens the bone matrix. This reduces tensile strength, causing brittle bones and fractures.

**10. Acute Intermittent Porphyria**
Porphobilinogen deaminase deficiency leads to accumulation of ALA and PBG. These neurotoxic intermediates cause abdominal and neuropsychiatric symptoms.

**11. Homocystinuria**
Cystathionine β-synthase deficiency leads to elevated homocysteine. This damages endothelium and increases risk of thrombosis.

**12. Oculocutaneous Albinism**
Tyrosinase deficiency impairs conversion of tyrosine to melanin. This results in hypopigmentation and visual defects.

**13. Down Syndrome (Trisomy 21)**
An extra chromosome 21 causes gene dosage imbalance. This disrupts normal development, leading to multisystem abnormalities.

**14. Immune Thrombocytopenic Purpura (ITP)**
Autoantibodies target platelet membrane glycoproteins. This leads to splenic destruction of platelets and thrombocytopenia.

**15. Non-ST Elevation Myocardial Infarction (NSTEMI**
Troponin is released from damaged cardiomyocytes. Its elevation indicates myocardial cell injury and necrosis.
