# Supplementary material for: The Palette of Science and Emotions: Art-Based Learning With Structured Peer Role-Plays for Early Clinical Exposure in Biochemistry
Source: MedEdPORTAL. 2026 May 19;22:11601. doi: 10.15766/mep_2374-8265.11601 (PMC13183865; doi:10.15766/mep_2374-8265.11601)
Supplement: Supplementary file 1 — Faculty Orientation.pptxCurated Artworks.docxActivity Instructions.docxRole-Play Resources.docxFacilitator Guide.docxPersonal Reflection Questionnaire.docxEvaluation Questionnaire.docxSemistructured Interview Guide.docxPostsession Assessment.docxConfidence Questionnaire.docx [file mep_2374-8265.11601-s001.zip › H. Semistructured Interview Guide.docx]

**Semi-Structured Interview Guide**

1. Can you describe your overall experience of the session?
2. What stood out to you most?
3. How did beginning with artwork influence your engagement with the clinical case?
4. Did the VTS discussion change how you approached the scenario?
5. In what ways did the session help you connect biochemical mechanisms to patient care?
6. Did your understanding of any pathway or concept change after the activity?
7. How did the roleplay influence your approach to clinical reasoning?
8. Did you find yourself thinking differently compared to traditional case discussions?
9. How did assuming a role (patient, caregiver, clinician) affect your thinking?
10. Did the activity change how you perceive patient emotions or suffering?
11. How did group interaction shape your learning?
12. Were there moments where peer perspectives expanded your understanding?
13. What emotions did you experience during the session?
14. Did these emotions influence your learning or reflection?
15. Has this session influenced how you plan to approach patients in the future?
16. Do you think this method improved your understanding of biochemistry beyond exams?
17. What aspects of the activity were most effective?
18. What could be improved?
19. Is there anything else about your experience that we have not discussed but you feel is important?
