# Supplementary material for: The Palette of Science and Emotions: Art-Based Learning With Structured Peer Role-Plays for Early Clinical Exposure in Biochemistry
Source: MedEdPORTAL. 2026 May 19;22:11601. doi: 10.15766/mep_2374-8265.11601 (PMC13183865; doi:10.15766/mep_2374-8265.11601)
Supplement: Supplementary file 1 — Faculty Orientation.pptxCurated Artworks.docxActivity Instructions.docxRole-Play Resources.docxFacilitator Guide.docxPersonal Reflection Questionnaire.docxEvaluation Questionnaire.docxSemistructured Interview Guide.docxPostsession Assessment.docxConfidence Questionnaire.docx [file mep_2374-8265.11601-s001.zip › D. Role-Play Resources.docx]

**(This appendix contains all materials needed for the STRUCTURED PEER ROLEPLAY ACTIVITY for Group 1-15)**

**Artwork disclaimer: Images are used as reflective prompts, not diagnostic representations. Artistic depictions may differ from the diseases discussed.**

**ART 1**

**STRUCTURED PEER ROLEPLAY ACTIVITY**

**Artwork Context**

A young soldier lies on a makeshift cot under trees in a remote conflict setting, barely conscious. A woman sits beside him, quietly caring for him. In the distance, tents from a temporary field hospital suggest constrained medical access and delayed transfer to advanced care. The scene reflects metabolic crisis in a resource-limited environment where laboratory confirmation and specialist support may not be immediately available.

**Learning Objectives**

By the end of this session, learners will be able to:

1. Describe patient suffering through visual and narrative cues.
2. Describe the biochemical basis and key clinical features of disease presented in the case scenarios.
3. Use Visual Thinking Strategies (VTS) and structured peer roleplays to reflect on patient and caregiver perspectives on illness and suffering.
4. Demonstrate empathetic communication, ethical reasoning, and collaborative clinical decision-making in the context of a biochemical disorder through a structured peer roleplay and guided reflection.
5. Reflect on emotions, communication, and the meaning of suffering using a structured reflection format.

**Clinical Scenario**

A 19-year-old soldier named Tom was rescued from the forest three days after he went missing during a battle in a remote field setting with limited access to advanced medical facilities. He had no food or water during this time and is now only partially conscious, confused, and showing signs of flapping tremor. He is breathing irregularly and sometimes mutters things that do not make sense.

His elder sister, Mary, a volunteer nurse, is sitting by his side. She says he was a healthy boy until now and insists, “He’s just weak from not eating.”

You are the team on duty in a temporary field hospital. A senior doctor has been called to join a junior doctor and an intern, who are attending to Tom. There is also a ward helper who noticed some unusual movements and behavioral changes. You must examine the patient, articulate your biochemical reasoning, and decide what can be done next.

Only one intravenous glucose infusion and a small batch of antibiotics are available. Dialysis and ammonia-lowering agents are not accessible at this facility. The ambulance will not arrive for 6 hours. His condition is worsening.

**Role Descriptions**

**Role Descriptions**

1. **Dr. Anita – Senior Doctor**
2. **Mr. Rohan – Intern**
3. **Mr. Tom – Patient**
4. **Ms. Mary – Sister and Caregiver**
5. **Mr. Ali – Ward Helper**

**Key Clinical and Biochemical Points to Elicit**

- Hyperammonemia signs: altered sensorium, asterixis, disorientation
- Urea cycle overload secondary to increased protein catabolism during starvation
- Ammonia crossing the blood–brain barrier leading to neurotoxicity
- Glucose infusion reducing protein breakdown and nitrogen load
- Limited treatment options without dialysis or scavenger therapy

Learners should also consider whether severe metabolic stress may unmask an underlying vulnerability in nitrogen metabolism and whether follow-up evaluation would be appropriate if the patient stabilizes.

**Dilemma to Resolve**

- You have limited supplies. Should you treat Tom aggressively or wait to see if he worsens further?
- Can you explain the biochemical basis of his condition to Mary in a way she understands and agrees with?
- What supportive measures can be implemented until transfer to higher-level care?
- How should scarce resources be allocated ethically in a constrained setting?

**Reflection Questions**

1. What clinical features most strongly supported your biochemical reasoning?
2. How did articulating the mechanism aloud influence your diagnostic confidence?
3. What emotions arose when communicating with a distressed caregiver?
4. How might resource limitations alter clinical decision-making?
5. In what ways does the artwork illuminate aspects of suffering not captured by laboratory reasoning?
6. Where does the artwork fail to represent the biomedical reality of the disease process?

**1. Dr. Anita – Senior Doctor**

You have served in military postings for a decade. Rather than immediately stating a diagnosis, you facilitate reasoning.

Ask the intern:

- What metabolic processes are activated during prolonged starvation?
- How does increased protein breakdown affect nitrogen metabolism?
- What explains the neurological signs?
- What interventions are feasible in this setting?

You guide the team to connect altered sensorium and asterixis to a biochemical mechanism. You clarify inaccuracies and structure the discussion.

You must also communicate clearly with Mary, who believes Tom only needs rest.

You are responsible for making the final treatment decision: whether to use the remaining intravenous glucose infusion now or reserve it for another unstable patient. Your role requires clinical judgment, ethical reasoning, and clear communication.

You must interact with all members of the team.

**2. Mr. Rohan – Intern**

This is your second week in the field unit. You observe that Tom is confused and demonstrates flapping tremor. You recall from biochemistry that ammonia can cross the blood–brain barrier and disrupt astrocyte metabolism.

You are encouraged to think and answer the questions by Dr.Anita

You attempt to explain the suspected biochemical process to Mary in simple language. You take relevant history and communicate preliminary impressions gently and respectfully.

**3. Mr. Tom – Patient**

You drift in and out of consciousness. You feel confused and disoriented. You say random phrases from childhood. Occasionally, you attempt to sit up but collapse.

You mostly remain minimally responsive, but when called by name or touched, you may react slowly or speak incoherently. You may have one brief moment of clarity when you squeeze your sister’s hand.

**4. Mrs. Mary – Sister and Caregiver**

You searched for your brother for three days and have just found him. You are convinced he is weak from starvation and exhaustion. You do not understand how a substance like ammonia could affect the brain.

You are emotional but willing to listen if explanations are clear. You want specific answers. You ask:

- What exactly is happening?
- Is this reversible?
- Are you sure this is not just dehydration?

You challenge vague explanations but respond to structured, compassionate communication.

**5. Mr. Ali – Ward Helper**

You help carry patients and distribute supplies. You noticed Tom’s flapping hand movements. You also recall seeing similar movements in another soldier who deteriorated rapidly.

You are hesitant to speak but provide crucial observational information when asked. You mention that Tom’s breath smells sweet and unusual.

You do not understand the science, but your observations are accurate and clinically valuable.

**ART 2**

**STRUCTURED PEER ROLEPLAY ACTIVITY**

**Context of the Artwork**

A young girl lies in bed, fragile and pale. Her hair is red, her skin pale, and she appears weak and possibly febrile. A woman sits beside her, head bowed in grief or prayer, holding her hand tightly. The room is dim. The scene conveys helplessness and anticipatory loss. While the original painting is historically associated with tuberculosis, this reflective roleplay explores a hematologic condition presenting with recurrent crises and systemic suffering

**Learning Objectives**

By the end of this session, students will be able to:

1. Describe patient suffering through visual and narrative cues.
2. Describe the biochemical basis and key clinical features of disease presented in the case scenarios.
3. Use Visual Thinking Strategies (VTS) and structured peer roleplays to reflect on patient and caregiver perspectives on illness and suffering.
4. Demonstrate empathetic communication, ethical reasoning, and collaborative clinical decision-making in the context of a biochemical disorder through a structured peer roleplay and guided reflection.
5. Reflect on emotions, communication, and the meaning of suffering using a structured reflection format.

**Clinical Scenario**

A 10-year-old girl named Anna has been brought to a district hospital with high fever, body aches, fatigue, and difficulty breathing. Her mother reports that similar episodes have occurred “every few months” over the past two years. The family recently relocated from a rural region where newborn screening programs were not consistently available. No prior confirmatory testing was performed, though she was treated several times at local clinics for presumed viral fever or chest infection.

She now presents with pallor, mild jaundice, chest pain, and pain in her legs and arms. Her oxygen saturation is borderline low. A peripheral blood smear and hemoglobin electrophoresis have been sent but results are pending. The working diagnosis includes a sickling disorder with possible acute chest syndrome.

The senior and junior doctors must evaluate her together. The intern is responsible for history-taking and physical examination. The patient is withdrawn and anxious. Her mother is frustrated and exhausted. A ward helper reports additional observations.

**Roles in This Reflective Roleplay**

1. Dr. Lakshmi – Senior Doctor
2. Dr. Imran – Medical Intern
3. Ms. Anna – Patient
4. Mrs. Maria – Mother
5. Mrs. Revathi – Ward Helper

**Key Clinical and Biochemical Points to Elicit**

- Hemoglobin S mutation: substitution of valine for glutamic acid in the beta globin chain
- Polymerization of hemoglobin S under low oxygen tension
- Vaso-occlusion leading to ischemia and pain crises
- Chronic hemolysis causing anemia and jaundice
- Infection, dehydration, or hypoxia as common triggers
- Acute chest syndrome as a life-threatening complication
- Importance of confirmatory testing and long-term follow-up

Learners should also consider implications for:

- Carrier status in parents
- Risk to siblings
- Need for genetic counseling and family education

**Dilemma to Resolve**

1. Should treatment for a suspected sickling crisis begin before laboratory confirmation?
2. How should the suspected diagnosis be explained clearly and respectfully to the mother?
3. What immediate interventions are necessary to prevent deterioration?
4. How should future family screening and counseling be addressed once the patient stabilizes?

**Reflection Questions**

1. What clinical findings most strongly supported your working diagnosis?
2. How did articulating the molecular mechanism strengthen your reasoning?
3. What communication strategies were effective when addressing caregiver frustration?
4. How does early recognition influence long-term outcomes?
5. In what ways does the artwork illuminate aspects of suffering not fully explained by laboratory data?
6. Where does the artwork fail to represent the biomedical mechanisms of the disease?

**1. Dr. Lakshmi – Senior Doctor**

You are calm and experienced in managing hemoglobinopathies. Rather than immediately declaring a diagnosis, you prompt the intern:

- What explains recurrent pain episodes?
- How does a single amino acid substitution alter red blood cell behavior?
- What mechanism connects hypoxia to worsening symptoms?
- What makes chest symptoms concerning in this context?

You facilitate reasoning from molecular change to systemic manifestation. You support early stabilization while awaiting laboratory confirmation.

You also speak to Mrs. Maria with clarity and empathy. You explain that this condition, if confirmed, is inherited and manageable, and that family members may require testing. You outline the importance of follow-up and preventive care.

**2. Dr. Imran – Medical Intern**

You take a detailed history and note recurrent pain episodes, jaundice, and prior misdiagnoses. You connect pallor and fatigue to hemolysis and recall that sickled cells obstruct small vessels.

You answer the questions asked by Dr. Lakshmi.

You attempt to explain the suspected condition to the mother in simple terms, avoiding technical language.

**3. Ms. Anna – Patient**

You are 10 years old. You feel very tired and your chest hurts when you breathe. Your legs ache deeply. You respond softly when spoken to kindly.

You are afraid of being scolded by hospital staff for crying loudly during painful procedures in the past, and you try to remain quiet even when in pain. You are not afraid of your mother, but you worry about being seen as “difficult” in the hospital.

You do not understand why these episodes keep happening.

**4. Mrs. Maria – Mother**

You are exhausted and anxious. This is the fourth severe episode in one year. Earlier visits resulted in reassurance without definitive testing. You feel unheard and worry that something serious has been missed.

You ask directly:

- What exactly is this condition?
- Is it curable?
- Why was this not diagnosed earlier?

You respond positively to structured explanations and clear next steps.

**5. Mrs. Revathi – Ward Helper**

You assist in patient care and observe details others overlook. You noticed Anna’s fingers appear slightly bluish and that she cries quietly at night from pain. You recall hearing another child in the ward diagnosed with a sickling disorder.

When asked, you share your observations calmly.

**ART 3**

**STRUCTURED PEER ROLEPLAY ACTIVITY**

**Context of the Artwork**

The painting shows a dim, crumbling room. A woman lies critically ill on a worn bed. A man, likely her brother or husband, sits beside her in visible despair, his hand resting on hers. There is no visible medical support, only quiet suffering within a constrained environment. Poverty and helplessness fill the space.

This scene evokes the social reality of individuals living with chronic inherited blood disorders in settings without routine newborn screening for hemoglobinopathies, where diagnosis may be delayed and long-term follow-up inconsistent.

For this Reflective Roleplay, the focus is beta thalassemia major that has remained undiagnosed or poorly managed due to fragmented access to care.

**Learning Objectives**

By the end of this session, learners will be able to

1. Describe patient suffering through visual and narrative cues.
2. Describe the biochemical basis and key clinical features of disease presented in the case scenarios.
3. Use Visual Thinking Strategies (VTS) and structured peer roleplays to reflect on patient and caregiver perspectives on illness and suffering.
4. Demonstrate empathetic communication, ethical reasoning, and collaborative clinical decision-making in the context of a biochemical disorder through a structured peer roleplay and guided reflection.
5. Reflect on emotions, communication, and the meaning of suffering using a structured reflection format.

**Clinical Scenario**

A 22-year-old woman named Rekha has been brought in unconscious by her brother. She has been increasingly tired and breathless over the past month. The family reports she has received blood transfusions intermittently since childhood but was never given a clear diagnosis. Formal newborn screening was not available in the region where she was born, and confirmatory testing was never completed.

She now appears pale, thin, and mildly jaundiced. Her spleen is palpable. Her brother recalls being told years ago that she might have a “blood-making problem,” but no structured long-term plan was established. She has missed follow-up for over two years due to financial constraints and geographic barriers to specialty care.

You are the team attending to her in the emergency department.

**Roles in This Reflective Roleplay**

1. **Dr. Neha – Senior Resident**
2. **Mr. Aditya – Final-year Medical Student**
3. **Mrs. Rekha – Patient**
4. **Mr. Manoj – Elder Brother and Caregiver**
5. **Mrs. Shanthi – Ward Attendant**

**Key Clinical and Biochemical Points to Elicit**

- Beta thalassemia involves reduced or absent beta globin chain synthesis
- Excess alpha chains precipitate, causing ineffective erythropoiesis
- Microcytic hypochromic anemia and chronic hemolysis
- Marrow expansion and splenomegaly
- Iron overload due to repeated transfusions without chelation
- Diagnosis through hemoglobin electrophoresis
- Regular transfusions, iron chelation therapy, and genetic counseling are essential

Learners should also address:

- Autosomal recessive inheritance
- Carrier testing for family members
- Implications for future pregnancies
- Long-term multidisciplinary follow-up

**Dilemma to Resolve**

1. How do you explain a likely lifelong inherited diagnosis to Manoj, who feels responsible for delayed care?
2. Do you initiate transfusion immediately despite suspected iron overload?
3. How do you balance urgent stabilization with discussion of long-term management and family screening?

**Reflection Questions**

1. What clinical findings most strongly suggested beta thalassemia?
2. How did understanding globin chain imbalance clarify the patient’s presentation?
3. How did financial and systemic barriers influence disease progression?
4. What role did empathy play when discussing lifelong genetic disease?
5. In what ways does the artwork illuminate aspects of chronic illness not captured by laboratory findings?
6. Where does the artwork fail to represent the biochemical mechanisms underlying the condition?

**1. Dr. Neha – Senior Resident**

You recognize features suggestive of poorly managed beta thalassemia major. Rather than directly announcing the diagnosis, you prompt Aditya:

- What explains the microcytic anemia?
- What happens when beta globin chains are absent?
- How does ineffective erythropoiesis lead to splenomegaly?
- Why does repeated transfusion create additional long-term risk?

You guide reasoning from molecular defect to systemic complications.

You also speak with Manoj. You explain that this condition is inherited in an autosomal recessive pattern and may affect other family members as carriers. You introduce the importance of genetic counseling and structured long-term follow-up, while prioritizing immediate stabilization.

**2. Mr. Aditya – Final-Year Medical Student**

You examine Rekha under supervision and note severe pallor, jaundice, and splenomegaly. You review prior transfusion history.

You answer the questions asked by Dr. Neha. You attempt to explain the condition to Manoj in clear, non-technical language. You discuss the need for regular follow-up, iron chelation therapy, and possible curative options such as bone marrow transplantation where available.

**3. Mrs. Rekha – Patient**

You are mostly unresponsive and extremely weak. You have lived with chronic illness but without a clear explanation. Occasionally, you murmur, “Will it stop now?” or “I don’t want more needles.”

You represent the lived burden of untreated inherited disease.

**4. Mr. Manoj – Brother and Caregiver**

You have raised your sister and managed her illness with limited guidance. You feel guilt and anger that no one explained the condition clearly before.

You ask:

- Is this curable?
- Will this keep happening?
- Does this mean our family carries something abnormal?

You respond to honest, structured explanations and practical next steps.

**5. Mrs. Shanthi – Ward Attendant**

You noticed Rekha’s jaundice and history of repeated transfusions. You quietly mention similarities with another patient diagnosed with thalassemia.

Your observational details contribute to pattern recognition.

**ART 4**

**STRUCTURED PEER ROLEPLAY ACTIVITY**

**Context of the Artwork**

A well-dressed young man sits across from a physician at a dinner table. The physician appears matter-of-fact, pointing at the food and objects around them. The title reads The Yellow Jaundice, and the tone is satirical, suggesting exaggeration or misinterpretation of symptoms. The image reflects tension between patient anxiety and medical interpretation, and the risk of overreaction to benign clinical findings.

For this Reflective Roleplay, the case explores a benign inherited disorder of bilirubin metabolism, emphasizing biochemical reasoning, diagnostic restraint, and patient-centered communication.

**Learning Objectives**

By the end of this session, learners will be able to

1. Describe patient suffering through visual and narrative cues.
2. Describe the biochemical basis and key clinical features of disease presented in the case scenarios.
3. Use Visual Thinking Strategies (VTS) and structured peer roleplays to reflect on patient and caregiver perspectives on illness and suffering.
4. Demonstrate empathetic communication, ethical reasoning, and collaborative clinical decision-making in the context of a biochemical disorder through a structured peer roleplay and guided reflection.
5. Reflect on emotions, communication, and the meaning of suffering using a structured reflection format.

**Clinical Scenario**

A 21-year-old college student named Ravi presents to a hepatology clinic accompanied by his uncle, Mr. Suresh. He reports fatigue and occasional mild nausea and says others have told him he “looks yellow.” He searched his symptoms online and is concerned about liver failure or hepatitis.

He reports that he has been eating very poorly for several days while preparing for his final examinations. He has not had fever, alcohol use, high-risk exposure, or recent illness.

On examination, he has mild scleral icterus. There is no hepatosplenomegaly, rash, or bleeding tendency. Complete blood count is normal. Liver function tests show mild isolated elevation of unconjugated bilirubin; all liver enzymes are within normal limits. Reticulocyte count is normal.

He has presented for evaluation three times in two weeks. In this setting, patients may directly request repeat laboratory testing through private diagnostic services without physician referral. Mr. Suresh is concerned and requests advanced imaging and “all scans possible.”

**Roles in This Reflective Roleplay**

- Dr. Parveen – Consultant in Hepatology
- Rishi – Third-year Medical Student
- Ravi – Patient
- Mr. Suresh – Uncle and Caregiver
- Ms. Naina – Laboratory Technician

**Key Clinical and Biochemical Points to Elicit**

- Gilbert syndrome involves reduced activity of the uridine diphosphate glucuronosyltransferase enzyme
- Results in isolated unconjugated hyperbilirubinemia
- Often triggered by fasting, stress, or illness
- No hemolysis and normal liver enzymes
- No treatment required
- Important to differentiate from hemolysis and hepatocellular injury

Learners should also briefly address:

- Autosomal recessive inheritance
- Benign prognosis
- Reassurance and education as primary management

**Dilemma to Resolve**

1. How do you explain to Ravi and Mr. Suresh that this condition is benign?
2. How do you avoid unnecessary investigations while preserving trust?
3. How do you validate anxiety without reinforcing illness behavior?

**Reflection Questions**

1. What clinical and laboratory findings supported a benign diagnosis?
2. How did understanding bilirubin metabolism shape your explanation?
3. How did you balance reassurance with diagnostic caution?
4. What ethical considerations arise when unnecessary investigations are requested?
5. In what ways does the artwork exaggerate or distort medical interpretation?
6. Where does the artwork fail to represent the biochemical mechanism underlying the condition?

**1. Dr. Parveen – Consultant in Hepatology**

You suspect Gilbert syndrome based on isolated unconjugated hyperbilirubinemia with normal liver enzymes and no hemolysis.

Rather than directly declaring the diagnosis, you prompt Rishi:

- What differentiates unconjugated from conjugated hyperbilirubinemia?
- What enzyme is responsible for bilirubin conjugation?
- Why might fasting worsen jaundice?
- What laboratory pattern would suggest hemolysis instead?

You guide reasoning from pathway to presentation.

You explain to Ravi and Mr. Suresh that this is a mild inherited condition involving reduced bilirubin conjugation. You clarify that it does not progress to liver failure and does not require treatment. You also discuss the genetic nature of the condition and reassure them regarding long-term health.

You discourage unnecessary imaging while validating their concerns.

**2. Mr. Rishi – Third-Year Medical Student**

You take history and examine Ravi. You consider hemolysis and liver disease but note that liver enzymes and reticulocyte count are normal.

You reason to questions asked by Dr. Parveen. Then you also attempt to explain the diagnosis in clear, non-technical language and practice calm reassurance.

**3. Mr. Ravi – Patient**

You are anxious and have read alarming online information. You are worried about serious liver disease. You ask repeatedly, “Is this serious?” and “Can this become cancer?”

You want clear, scientific explanation without dismissal.

**4. Mr. Suresh – Uncle and Caregiver**

You are protective and anxious. In prior medical visits elsewhere, doctors reassured Ravi without detailed explanation, which left you feeling unheard and uncertain. Because of that experience, you worry that something serious might again be overlooked.

You push for imaging and advanced tests, not out of distrust of this team specifically, but due to fear of missing a hidden diagnosis. You calm down when given structured explanations and clear follow-up plans.

**5. Ms. Naina – Laboratory Technician**

You work in the diagnostic laboratory where patients can directly request repeat blood tests. You have seen Ravi several times over the past two weeks for repeat liver function testing.

You mention to Rishi that his enzyme levels have remained stable and that stress and irregular meals are common triggers for mild bilirubin elevation.

You provide contextual insight without influencing the diagnosis.

**ART – 5**

**STRUCTURED PEER ROLEPLAY ACTIVITY**

**Context of the Artwork**

In this theatrical domestic scene, a man has collapsed at a gathering, possibly unconscious. Food and drink are scattered; some guests are alarmed, others stunned. A woman kneels beside him, while others hesitate or rush forward. The painting suggests sudden health deterioration in a social setting, possibly after prolonged alcohol use.

This reflective roleplay explores advanced alcohol-related liver disease presenting with decompensated cirrhosis and gastrointestinal bleeding.

**Learning Objectives**

By the end of this session, learners will be able to

1. Describe patient suffering through visual and narrative cues.
2. Describe the biochemical basis and key clinical features of disease presented in the case scenarios.
3. Use Visual Thinking Strategies (VTS) and structured peer roleplays to reflect on patient and caregiver perspectives on illness and suffering.
4. Demonstrate empathetic communication, ethical reasoning, and collaborative clinical decision-making in the context of a biochemical disorder through a structured peer roleplay and guided reflection.
5. Reflect on emotions, communication, and the meaning of suffering using a structured reflection format.

**Clinical Scenario**

A 47-year-old man named Mr. Raghav collapses at a family gathering. He has consumed alcohol daily for many years but reports no prior major illness. Over the past several weeks, family members noticed increasing confusion, disturbed sleep, abdominal swelling, and yellowing of the eyes. Earlier today, after heavy drinking, he collapsed and passed black stool.

He is brought to the emergency unit by his wife, Mrs. Kavita, and a close family friend, Mr. Sharma. His elderly father arrives shortly afterward and expresses concern that symptoms were minimized at home.

On examination, his pulse is thready. He is confused and disoriented. His abdomen is distended. Laboratory investigations show low serum albumin, elevated international normalized ratio, and elevated serum ammonia.

You are the team on call.

**Roles in This Reflective Roleplay**

1. Dr. Meera – Medical Officer
2. Mr. Harsh – Senior Medical Student
3. Mr. Raghav – Patient
4. Mrs. Kavita – Wife and Primary Caregiver
5. Mr. Sharma – Family Friend

**Key Clinical and Biochemical Points to Elicit**

1. Chronic alcohol use alters the nicotinamide adenine dinucleotide to nicotinamide adenine dinucleotide hydrogen ratio
2. Disruption of hepatic metabolism leading to steatosis, fibrosis, and cirrhosis
3. Impaired ammonia detoxification contributing to hepatic encephalopathy
4. Reduced albumin synthesis and impaired clotting factor production
5. Portal hypertension leading to variceal bleeding
6. Emergency management: airway protection, intravenous fluids, thiamine, lactulose, and blood products when indicated

Learners should also address:

- Reversibility versus chronic damage
- Long-term alcohol cessation
- Family counseling and rehabilitation support
- Ethical communication without stigma

**Dilemma to Resolve**

1. How do you stabilize the patient without a complete prior history?
2. How do you communicate seriousness without blame or moral judgment?
3. How do you involve family members appropriately while respecting patient autonomy?
4. What long-term plan should be discussed if he survives this episode?

**Reflection Questions**

- What did this case teach you about biochemical reasoning in liver disease?
- How did the social setting influence your approach to communication?
- What did you learn about alcohol-related stigma and chronic illness care?
- How will this shape your role as a future clinician in similar settings?
- In what ways does the artwork represent this medical interpretation?
- Where does the artwork fail to represent the biochemical mechanism underlying the condition?

**1. Dr. Meera – Medical Officer**

You assess Mr. Raghav as having likely decompensated cirrhosis with gastrointestinal bleeding and hepatic encephalopathy.

Rather than directly lecturing, you ask Mr. Harsh to think through the case aloud:

- What explains the confusion?
- How does chronic alcohol intake alter hepatic metabolism?
- Why are albumin and clotting parameters abnormal?
- What is the mechanism behind black stool?

You expect structured clinical reasoning appropriate for a senior medical student, including history of present illness, relevant past medical history, alcohol intake pattern, medication history, and focused examination findings.

You initiate stabilization and communicate clearly with Mrs. Kavita and Mr. Raghav’s father, avoiding judgmental language.

**2. Mr. Harsh – Senior Medical Student**

You are in your final year of medical training and have completed prior instruction in history-taking and hepatology.

When prompted by Dr. Meera, you organize information systematically:

- History of present illness
- Past medical history
- Alcohol use history
- Medication and bleeding history
- Physical examination findings

You think aloud and answer the questions asked by Dr. Meera, linking pathophysiology to clinical presentation. You avoid assigning blame and focus on medical reasoning.

**3. Mr. Raghav – Patient**

You are semi-conscious, confused, and intermittently agitated. You mutter phrases such as “It was just one drink.”

Your condition requires others to speak on your behalf and make urgent decisions.

**4. Mrs. Kavita – Wife and Primary Caregiver**

You have lived with your husband’s drinking habits for years. You noticed swelling and confusion but hoped it would improve. You feel guilt and fear.

You ask:

- Will he survive?
- Could this have been prevented?
- What must we do now?

You respond positively to calm, structured explanations.

**5. Mr. Sharma – Family Friend**

You are a long-time friend who often shared meals and drinks with Mr. Raghav. You initially minimize the severity, saying, “He was fine this morning.”

You gradually recognize the seriousness of his condition and ask what could have been done earlier.

**ART 6**

**STRUCTURED PEER ROLEPLAY ACTIVITY**

**Context of the Artwork**

The painting depicts a mother holding a pale, limp infant in her lap. The child appears critically ill. The mother presses a handkerchief to her face, suggesting grief or shock. The stillness of the scene evokes irreversible neurological injury and the possibility of an undetected metabolic condition.

This reflective roleplay explores phenylketonuria in a setting where routine newborn screening for metabolic disorders is not universally implemented.

**Learning Objectives**

By the end of this session, learners will be able to

1. Describe patient suffering through visual and narrative cues.
2. Describe the biochemical basis and key clinical features of disease presented in the case scenarios.
3. Use Visual Thinking Strategies (VTS) and structured peer roleplays to reflect on patient and caregiver perspectives on illness and suffering.
4. Demonstrate empathetic communication, ethical reasoning, and collaborative clinical decision-making in the context of a biochemical disorder through a structured peer roleplay and guided reflection.
5. Reflect on emotions, communication, and the meaning of suffering using a structured reflection format.

**Clinical Scenario**

An 8-month-old infant, Laya, is brought to a rural pediatric outpatient department in a region where universal newborn metabolic screening is not consistently available. She presents with poor growth, reduced activity, and global developmental delay. She has pale skin, a musty body odor, and intermittent episodes of irritability followed by limpness.

She was born full term but did not undergo newborn metabolic screening due to local logistical limitations. According to prior clinic notes, she was described as appearing constitutionally weak but without signs of acute illness.

Her mother, Ms. Geetha, reports that something “felt wrong” since the second month of life. A community health worker, Ms. Jansi, accompanies the family and provides additional observations from the patient’s home.

The infant appears lethargic. The family seeks definitive answers.

**Roles in This Reflective Roleplay**

1. Dr. Rohit – Pediatric Consultant
2. Ms. Divya – Pediatric Intern
3. Laya – Infant Patient
4. Ms. Geetha – Mother
5. Ms. Jansi – Accredited Social Health Activist (ASHA Worker)

**Key Clinical and Biochemical Points to Elicit**

1. PKU is caused by a deficiency of phenylalanine hydroxylase or, more rarely, dihydropteridine reductase.
2. Accumulation of phenylalanine leads to neurotoxicity
3. Clinical features: developmental delay, seizures, musty odor, hypopigmentation
4. Early detection through newborn screening prevents neurological damage
5. Lifelong dietary restriction and metabolic follow-up are required
6. Autosomal recessive inheritance; counseling of parents is essential

**Dilemma to Resolve**

1. How do you explain to Ms. Geetha that this is a genetic metabolic disorder and not the result of parenting?
2. What immediate investigations and dietary interventions should be initiated?
3. How can long-term metabolic follow-up and nutritional support be coordinated in a rural setting?

**Reflection Questions**

1. How did biochemical pathway knowledge guide your diagnosis?
2. What communication strategies reduced parental guilt?
3. How did the health worker contribute to recognition of delayed development?
4. What ethical responsibility exists when screening systems are limited?
5. In what ways does the artwork illuminate parental suffering beyond laboratory findings?
6. Where does the artwork fail to represent the biochemical mechanism of disease?

**1. Dr. Rohit – Pediatric Consultant**

You have experience with inborn errors of metabolism. Based on developmental delay, hypopigmentation, and characteristic odor, you suspect phenylketonuria.

Rather than declaring the diagnosis immediately, you ask Ms. Divya to reason through:

• Which amino acid pathway may be disrupted?

• What enzyme is deficient?

• Why does phenylalanine accumulation cause neurological damage?

• Why is early screening critical?

You explain to Ms. Geetha that this is an inherited metabolic condition and not caused by feeding practices or neglect. You discuss confirmatory blood phenylalanine testing and dietary management.

You briefly address autosomal recessive inheritance and recommend genetic counseling for the parents regarding future pregnancies.

**2. Ms. Divya – Pediatric Intern**

You have completed foundational biochemistry and pediatric training. When prompted by Dr. Rohit, you organize your reasoning:

- History of developmental milestones
- Feeding pattern
- Family history
- Physical examination findings

You think aloud and answer the questions asked by Dr. Rohit, linking the disrupted phenylalanine pathway to neurotoxicity.

You explain the condition to Ms. Geetha in simple language when requested and outline diagnostic testing and dietary referral under supervision.

**3. Laya – Infant Patient**

You are largely unresponsive, with poor muscle tone and minimal eye contact. You cry sharply when stimulated but do not engage visually. Your condition shapes the urgency and emotional tone of the discussion.

**4. Ms. Geetha – Mother**

You are 23 years old and this is your first child. You were reassured previously but sensed ongoing developmental concerns. You feel guilt and fear that you may have caused harm.

You ask:

- Was this my fault?
- Is this curable?
- Will she ever develop normally?

You respond positively to clear explanations and a structured care plan.

**5. Ms. Jansi – ASHA**

You referred Laya after observing delayed milestones and feeding difficulties in the patient’s home. You provide contextual information about diet, community access, and follow-up challenges.

You ask what can be done to prevent similar cases in the village.

**ART – 7**

**STRUCTURED PEER ROLEPLAY ACTIVITY**

**Context of the Artwork**This painting captures an emotionally intimate moment. A weakened young woman collapses into the arms of a grieving partner. His grip is protective and desperate. Her body appears pale and frail. The stark, crumpled sheets suggest prolonged illness and physical decline. The scene evokes progressive, undiagnosed disease reaching a critical point.

This reflective roleplay explores hereditary spherocytosis presenting with aplastic crisis.

**Learning Objectives**By the end of this session, learners will be able to

1. Describe patient suffering through visual and narrative cues.
2. Describe the biochemical basis and key clinical features of disease presented in the case scenarios.
3. Use Visual Thinking Strategies (VTS) and structured peer roleplays to reflect on patient and caregiver perspectives on illness and suffering.
4. Demonstrate empathetic communication, ethical reasoning, and collaborative clinical decision-making in the context of a biochemical disorder through a structured peer roleplay and guided reflection.
5. Reflect on emotions, communication, and the meaning of suffering using a structured reflection format.

**Clinical Scenario**
A 19-year-old woman, Ms. Eva, is brought to the emergency department by her partner, Mr. Markus. She is severely pale, thin, and minimally responsive. Her lips are dry and her sclerae appear icteric.

Mr. Markus reports that she has always seemed fatigued and “a little sickly,” but over the past week she developed fever and sore throat. Since then, she has eaten very little and became progressively weaker before collapsing that morning.

On examination, her pulse is weak and she feels cold to touch. The spleen is enlarged. Laboratory investigations reveal severe anemia, low reticulocyte count, elevated indirect bilirubin, and spherocytes on peripheral smear. The clinical picture is consistent with hereditary spherocytosis complicated by aplastic crisis, likely triggered by viral infection.

Mr. Markus has been her primary support for several years.

**Roles in This Reflective Roleplay**

1. Dr. Latha – Hematology Registrar
2. Mr. Praveen – Second-Year Medical Student
3. Ms. Eva – Patient
4. Mr. Markus – Partner and Caregiver
5. Rev. Miriam – Pastoral Counsellor

**Key Clinical and Biochemical Points**

1. Red blood cell membrane protein defects (spectrin, ankyrin) lead to spherocyte formation
2. Spherocytes are sequestered and destroyed in the spleen, causing chronic hemolysis
3. Clinical signs include pallor, jaundice, splenomegaly, and fatigue
4. Aplastic crisis results in a sudden fall in red blood cell count due to bone marrow suppression, often following viral infection
5. Elevated indirect bilirubin reflects ongoing hemolysis
6. Management includes transfusion, folate supplementation, monitoring, and possible future splenectomy
7. Autosomal dominant inheritance; family screening may be indicated

**Dilemma to Resolve**

1. How do you explain a previously undiagnosed hereditary disorder during an acute crisis?
2. How do you support Mr. Markus without reinforcing self-blame?
3. What immediate stabilization is required, and how should long-term care and family screening be addressed?

**Reflection Questions**

1. What laboratory findings guided your biochemical reasoning?
2. How did understanding membrane protein defects clarify the clinical picture?
3. What communication strategies reduced caregiver guilt?
4. How does this case reshape your understanding of chronic hereditary illness?
5. In what ways does the artwork convey prolonged suffering?
6. Where does the artwork not reflect the specific biochemical mechanism of disease?

**1. Dr. Latha – Hematology Registrar**

You recognize the likely diagnosis of hereditary spherocytosis with aplastic crisis.

You ask Mr. Praveen to think through the case aloud:

- What structural proteins maintain red blood cell shape?
- Why are spherocytes prone to splenic destruction?
- Why does reticulocyte count fall during aplastic crisis?
- How does viral infection contribute to marrow suppression?

You explain the biochemical mechanism clearly to Mr. Markus, emphasizing that this is an inherited structural defect and not caused by negligence.

You discuss immediate stabilization (transfusion if indicated) and future evaluation, including family history and possible screening.

**2. Mr. Praveen – Second-Year Medical Student**

You have completed foundational hematology and membrane physiology modules.

When prompted by Dr. Latha, you organize:

- History of chronic fatigue
- Recent viral illness
- Physical findings
- Laboratory abnormalities

You respond to Dr. Latha’s questions by linking membrane protein defects to hemolysis and marrow compensation failure.

You communicate carefully with Mr. Markus when asked, avoiding technical jargon.

**3. Ms. Eva – Patient**

You are minimally responsive. When briefly alert, you whisper, “Is it happening again?” or “Can I go home?”

Your condition heightens the urgency of discussion.

**4. Mr. Markus – Partner and Caregiver**

You have supported Ms. Eva through years of unexplained fatigue and recurrent illness. You feel guilt for not pursuing more investigations earlier.

You ask:

- Will she survive?
- Was this missed before?
- Could this happen again?

You seek honesty and clarity.

**5. Rev. Miriam – Pastoral Counsellor**

You are present to provide emotional support. Given the family-centered context of care in this setting, you ask whether extended family members should be informed and involved in future decision-making, while respecting that medical consent for an adult patient rests with Ms. Eva once she is capable.

You help translate complex explanations into emotionally meaningful language for Mr. Markus.

**ART – 8**

**STRUCTURED PEER ROLEPLAY ACTIVITY**

**Context of the Artwork**
The painting portrays a woman standing with her torso exposed. Her spine appears fractured and reinforced by a metal rod. Nails pierce her body. The background is barren and stark. The image evokes chronic pain, structural fragility, and the emotional burden of invisible illness.

This reflective roleplay explores secondary osteoporosis in a young adult.

**Learning Objectives**
By the end of this session, learners will be able to

1. Describe patient suffering through visual and narrative cues.
2. Describe the biochemical basis and key clinical features of disease presented in the case scenarios.
3. Use Visual Thinking Strategies (VTS) and structured peer roleplays to reflect on patient and caregiver perspectives on illness and suffering.
4. Demonstrate empathetic communication, ethical reasoning, and collaborative clinical decision-making in the context of a biochemical disorder through a structured peer roleplay and guided reflection.
5. Reflect on emotions, communication, and the meaning of suffering using a structured reflection format.

**Clinical Scenario**

A 22-year-old medical student, Ms. Shruti, presents with progressive back pain for over one year. The pain began during a period of academic stress and was initially attributed to posture. It has gradually worsened, limiting her ability to sit through lectures and participate in physical activity.

She reports irregular menstrual cycles for the past 10 months, significant weight loss, and reduced dietary intake during exam periods. She has a history of childhood asthma and has intermittently used inhaled corticosteroids for several years. There is no history of trauma.

Recent imaging shows compression fractures of the lower thoracic vertebrae. Dual-energy X-ray absorptiometry reveals a significantly reduced T-score for age. Laboratory tests demonstrate low serum vitamin D and borderline low serum estrogen levels.

She is brought to clinic by her roommate, Ms. Aanya, after collapsing while attempting to get out of bed.

**Roles in This Reflective Roleplay**

- Dr. Sameer – Senior Orthopedic Consultant
- Ms. Neha – Third-Year Medical Student
- Ms. Shruti – Patient
- Ms. Aanya – Roommate and Peer
- Ms. Rukmini – College Psychological Counsellor

**Key Clinical and Biochemical Points**

- Osteoporosis involves reduced bone mass and microarchitectural deterioration
- Secondary causes in young adults include:
  - Chronic corticosteroid exposure
  - Hypoestrogenism (irregular menses)
  - Low body weight
  - Vitamin D deficiency
  - Inadequate calcium intake
- Reduced estrogen increases osteoclast-mediated bone resorption
- Corticosteroids decrease osteoblast function and calcium absorption
- Diagnosis includes bone mineral density testing and laboratory evaluation
- Management includes nutritional correction, vitamin D supplementation, weight restoration, possible pharmacotherapy, and psychological support

**Dilemma to Resolve**

1. How do you explain fragility fractures to a young, high-functioning medical student?
2. How do you address lifestyle factors without blame?
3. How should medical, nutritional, and psychological interventions be integrated?

**Reflection Questions**

1. What biochemical mechanisms explain accelerated bone loss in this patient?
2. How did endocrine and nutritional factors contribute to disease progression?
3. How did the artwork shape your understanding of invisible fragility?
4. What communication strategies reduce shame in high-achieving patients?
5. In what ways does this artwork illuminate aspects of the patient’s lived experience not captured by laboratory findings?
6. Where does the artwork fail to represent the biomedical reality of secondary osteoporosis?

**1. Dr. Sameer – Senior Orthopedic Consultant**

You recognize secondary osteoporosis due to combined risk factors: chronic corticosteroid exposure, hypoestrogenism from menstrual irregularity, weight loss, and vitamin D deficiency.

Rather than providing immediate conclusions, you ask Ms. Neha:

- How does estrogen regulate bone turnover?
- What effect do corticosteroids have on osteoblasts?
- Why does low vitamin D impair bone mineralization?
- How do nutritional deficits contribute to fracture risk?

You explain to Ms. Shruti that osteoporosis can occur in young individuals when hormonal and metabolic balance is disrupted. You emphasize that this is a medical condition requiring multidisciplinary care.

You outline treatment including nutritional rehabilitation, vitamin D and calcium supplementation, possible pharmacologic therapy, and coordinated psychological support.

**2. Ms. Neha – Third-Year Medical Student**

You identify with Shruti’s experience. You have completed foundational instruction in bone physiology and endocrine regulation.

When prompted by Dr. Sameer, you organize your reasoning:

- History of menstrual irregularity
- Steroid exposure
- Nutritional history
- Imaging findings

You respond to Dr. Sameer’s questions by linking estrogen deficiency and corticosteroid use to increased osteoclast activity and reduced bone formation.

You explain osteoporosis to Ms. Shruti in accessible language when asked.

**3. Ms. Shruti – Patient**

You are 22 years old and academically high-performing. You minimized your pain and dietary changes during stressful periods. You are surprised and distressed by the diagnosis.

You say, “But I am not old,” and “How can my bones be fragile?”

You are resistant initially but open to structured explanation and reassurance.

**4.. Ms. Aanya – Roommate and Peer**

You have observed Ms. Shruti skipping meals, losing weight, and limiting social interaction during exam periods. You once noticed her fall but she refused help.

You provide collateral history when asked and seek guidance on how to support her recovery.

**5. Ms. Rukmini – College Psychological Counsellor**

You are invited to support emotional adjustment. You discuss stress, perfectionism, body image, and burnout.

You emphasize that chronic pain and metabolic stress are not personal failures.

You collaborate with the medical team to integrate psychological care into recovery.

**ART – 9**

**STRUCTURED PEER ROLEPLAY ACTIVITY**

**Context of Art**
The artwork portrays a young individual standing in guarded stillness. The posture appears tense yet controlled, with elongated hands drawn inward. The face is partially turned away, suggesting internalized suffering. The body appears fragile yet resilient. The image evokes the lived experience of chronic vulnerability, pain that is neither fully visible nor fully understood.

This reflective roleplay explores osteogenesis imperfecta in a child with recurrent fractures and limited continuity of care.

**Learning Objectives**
By the end of this session, learners will be able to

1. Describe patient suffering through visual and narrative cues.
2. Describe the biochemical basis and key clinical features of disease presented in the case scenarios.
3. Use Visual Thinking Strategies (VTS) and structured peer roleplays to reflect on patient and caregiver perspectives on illness and suffering.
4. Demonstrate empathetic communication, ethical reasoning, and collaborative clinical decision-making in the context of a biochemical disorder through a structured peer roleplay and guided reflection.
5. Reflect on emotions, communication, and the meaning of suffering using a structured reflection format.

**Clinical Scenario**
A nine-year-old girl, Latha, is brought to a primary health center by her elder sister, **Ms. Meena**. Latha has sustained at least six fractures since the age of three. She ambulates with crutches. Her left leg is bowed. Her sclerae appear distinctly blue. Her limbs are thin, and prior records indicate multiple healed fractures.

The family relocates frequently for employment and does not have stable housing. She was previously treated for nutritional rickets at another facility; however, supplementation did not reduce fracture frequency.

She struggles to attend school consistently because of mobility limitations. When asked how she feels, she says softly, “I do not want to fall again.”

No formal genetic evaluation has been performed.

**Roles in This Reflective Roleplay**

1. Dr. Anwar – Orthopedic Consultant
2. Ms. Harini – Final-Year Medical Student
3. Latha – Patient
4. Ms. Meena – Elder Sister and Caregiver
5. Ms. Devika – Community Disability Worker

**Key Clinical and Biochemical Points**

1. Osteogenesis imperfecta results from mutations affecting type I collagen synthesis
2. Type I collagen provides tensile strength to bone matrix
3. Defective collagen leads to brittle bones, recurrent fractures, deformities, and blue sclera
4. Fractures occur with minimal trauma due to structural weakness
5. Often autosomal dominant inheritance; family history should be explored
6. Diagnosis is primarily clinical, supported by imaging and genetic testing
7. Management includes fracture stabilization, bisphosphonates where available, calcium and vitamin D optimization, orthopedic correction, and rehabilitation
8. Psychosocial support and disability services are essential for long-term care

**Dilemma to Resolve**

1. How do you explain a lifelong connective tissue disorder to a child in age-appropriate language?
2. How do you clarify that this condition differs from nutritional rickets?
3. How do you support **Ms. Meena**, who feels responsible for her sister’s repeated injuries and delayed diagnosis?
4. How should long-term follow-up and possible family screening be addressed?

**Reflection Questions**

1. How did clinical signs such as blue sclera and recurrent fractures guide your biochemical reasoning?
2. How does defective type I collagen alter bone strength at the molecular level?
3. What communication strategies are appropriate when speaking directly to a child about a chronic genetic condition?
4. How does socioeconomic instability influence diagnosis and continuity of care?
5. In what ways does the artwork illuminate the emotional containment of chronic fragility?
6. Where does the artwork fail to represent the specific biochemical defect underlying osteogenesis imperfecta?

**1. Dr. Anwar – Orthopedic Consultant**

You recognize that the pattern of recurrent fractures, blue sclera, and failed response to supplementation suggests osteogenesis imperfecta rather than nutritional rickets.

Instead of stating the diagnosis immediately, you ask **Ms. Harini** to reason through:

- What is the function of type I collagen in bone matrix?
- Why would collagen defects lead to recurrent fractures?
- Why would vitamin supplementation alone be insufficient?
- What inheritance pattern is commonly associated?

You explain to **Ms. Meena** that this is a genetic connective tissue disorder and not caused by caregiving practices or neglect.

You outline referral to a tertiary center for further evaluation, possible bisphosphonate therapy, rehabilitation, and discussion of family history and genetic counseling.

You speak directly to Latha in simple, reassuring language.

**2. Ms. Harini – Final-Year Medical Student**

You conduct a structured history including fracture frequency, mechanism of injury, developmental milestones, dietary history, and family history.

When prompted by **Dr. Anwar**, you explain how defective collagen disrupts bone matrix integrity and increases fragility.

You speak gently with Latha about school and her goals, demonstrating age-appropriate communication.

You explain the condition to **Ms. Meena** in clear language when asked.

**3. Latha – Patient**

You are nine years old and have experienced repeated fractures. You move cautiously and avoid sudden movements.

You feel that you are a burden to your family because of your frequent injuries, even though your sister supports you.

You ask quietly:

- “Can my bones become stronger?”
- “Will I be able to walk without fear?”

You respond positively to being addressed directly and respectfully.

**4. Ms. Meena – Elder Sister and Caregiver**

You are seventeen and have taken on significant caregiving responsibility.

You worry that you did not recognize the seriousness earlier and ask:

- Is this curable?
- Did we miss something important?
- What can we realistically do now?

You seek reassurance that this condition is not the result of family neglect.

You want a practical plan that considers financial and mobility challenges.

**5. Ms. Devika – Community Disability Worker**

You are familiar with available community-based disability services.

You provide information regarding assistive devices, school accommodations, transport support, and nutritional programs.

You emphasize inclusion and long-term support.

You help translate medical recommendations into feasible community-level action.

**ART – 10**

**STRUCTURED PEER ROLEPLAY ACTIVITY**

**Context of the Artwork**

Three figures stand in close proximity yet appear emotionally distant. The father’s expression is wide-eyed and tense. The mother crouches inward. The child stands between them, staring ahead. The posture of each figure reflects vigilance and inherited fear. The image evokes the burden of something unseen, genetic, emotional, and recurring.

This reflective roleplay explores acute intermittent porphyria presenting as a neurovisceral emergency.

**Learning Objectives**
By the end of this session, learners will be able to

1. Describe patient suffering through visual and narrative cues.
2. Describe the biochemical basis and key clinical features of disease presented in the case scenarios.
3. Use Visual Thinking Strategies (VTS) and structured peer roleplays to reflect on patient and caregiver perspectives on illness and suffering.
4. Demonstrate empathetic communication, ethical reasoning, and collaborative clinical decision-making in the context of a biochemical disorder through a structured peer roleplay and guided reflection.
5. Reflect on emotions, communication, and the meaning of suffering using a structured reflection format.

**Clinical Scenario**

A 21-year-old woman, **Ms. Suhani**, is brought to the emergency department with severe abdominal pain, nausea, restlessness, and confusion. She has no fever, imaging studies were normal, and no surgical etiology was identified. Her vital signs are stable, but she is pacing, clutching her abdomen, and unable to clearly describe her pain. This is her third similar episode in two years.

Her family reports mood changes and anxiety for one week prior to presentation. Her father, **Mr. Raj**, recalls that his sister died in her twenties from an undiagnosed “blood disorder.” A freshly voided urine sample appears dark reddish-brown.

The attending physician considers acute intermittent porphyria and initiates urgent metabolic evaluation.

**Roles in This Reflective Roleplay**

1. Dr. Mahesh – Emergency Medicine Senior Physician
2. Ms. Isha – Third-Year Medical Student
3. Ms. Suhani – Patient
4. Mr. Raj – Father
5. Ms. Minal – Emergency Nurse

**Key Clinical and Biochemical Points**

1. Acute intermittent porphyria (AIP) results from deficiency of porphobilinogen deaminase
2. Attacks are precipitated by stress, fasting, certain drugs, hormones, and bleeding
3. Accumulation of aminolevulinic acid and porphobilinogen causes neurovisceral symptoms
4. Clinical features include severe abdominal pain, psychiatric symptoms, peripheral neuropathy, and dark or reddish urine
5. Porphobilinogen elevation supports the diagnosis of AIP; aminolevulinic acid testing helps distinguish AIP from aminolevulinate dehydratase deficiency and lead poisoning
6. AIP is autosomal dominant; family history and counseling are important
7. Management includes high-carbohydrate intake, intravenous glucose, avoidance of precipitating drugs, and hemin therapy when indicated

**Dilemma to Resolve**

1. How do you explain a rare metabolic disorder to a patient in acute distress?
2. What medications must be avoided to prevent worsening of symptoms?
3. How do you counsel a family with incomplete understanding of a possible inherited disorder?

**Reflection Questions**

1. What clinical features helped distinguish this case from a surgical or psychiatric emergency?
2. How does disruption of the heme biosynthesis pathway lead to neurovisceral symptoms?
3. What communication strategies are necessary during acute metabolic crises?
4. How should family members be counseled about inheritance and future risk?
5. In what ways does the artwork illuminate inherited fear and emotional containment?
6. Where does the artwork fail to represent the biochemical complexity of acute intermittent porphyria?

**1. Dr. Mahesh – Emergency Medicine Senior Physician**

You recognize that the absence of surgical findings and the presence of neuropsychiatric symptoms suggest a metabolic etiology.

You ask **Ms. Isha** to reason through:

- Which pathway produces heme?
- What enzyme is deficient in AIP?
- Why does porphobilinogen accumulate?
- Why is urine dark during attacks?
- What drugs must be avoided?

You order urine porphobilinogen and aminolevulinic acid testing.

You begin intravenous glucose to suppress hepatic heme synthesis and consider hemin therapy.

You explain to **Mr. Raj** that this is a genetic enzyme deficiency that may have affected his sister and discuss the need for family evaluation.

**2. Ms. Isha – Third-Year Medical Student**

You organize the case systematically:

- Character of abdominal pain
- Prior similar episodes
- Precipitating factors
- Family history

When prompted by **Dr. Mahesh**, you explain how porphobilinogen deaminase deficiency leads to toxic precursor accumulation.

You communicate with **Mr. Raj** in accessible language when asked.

**3. Ms. Suhani – Patient**

You are in severe pain and feel frightened and disoriented. You fear that others may dismiss your symptoms as psychological.

You say, “Something is burning inside,” and “Please don’t leave me.”

You respond positively when someone acknowledges your pain as real.

**4. Mr. Raj – Father**

You remember your sister’s unexplained illness and death. You fear recurrence in your daughter.

You ask:

- Is this genetic?
- Could this have been prevented?
- Will it happen again?

You need clear information without alarm.

**5. Ms. Minal – Emergency Nurse**

You recall Ms. Suhani’s prior visits and the uncertainty surrounding them.

You quietly share observations about the timing of episodes and prior psychiatric referral.

You help ensure continuity of care and support diagnostic clarity.

**ART – 11**

**STRUCTURED PEER ROLEPLAY ACTIVITY**

**Context of the Artwork**
A young boy rests in bed, pale and fatigued, holding a doll loosely in his hand. A woman sits nearby, knitting quietly. The light is soft, but the atmosphere is heavy. This image reflects prolonged illness rather than acute distress. The child’s posture is passive, and his grip appears weak, suggesting ongoing frailty. It is a portrait of chronic concern, where uncertainty has become routine.

This reflective roleplay explores homocystinuria presenting with developmental delay and thrombotic complications.

**Learning Objectives**
By the end of this session, learners will be able to

1. Describe patient suffering through visual and narrative cues.
2. Describe the biochemical basis and key clinical features of disease presented in the case scenarios.
3. Use Visual Thinking Strategies (VTS) and structured peer roleplays to reflect on patient and caregiver perspectives on illness and suffering.
4. Demonstrate empathetic communication, ethical reasoning, and collaborative clinical decision-making in the context of a biochemical disorder through a structured peer roleplay and guided reflection.
5. Reflect on emotions, communication, and the meaning of suffering using a structured reflection format.

**Clinical Scenario**
A 7-year-old boy, **Rithvik**, is brought to a pediatric clinic by his grandmother, **Ms. Radha**. She reports that he has difficulty in school, struggles with coordination, and tires easily. Recently, he developed swelling and pain in his left leg and was treated for deep vein thrombosis.

On examination, he is thin with long limbs, mild scoliosis, and poor vision. His eyes appear misaligned. His grandmother says, “He’s always been delicate. Doctors said he’d catch up.”

The physician suspects homocystinuria and orders a plasma amino acid profile. Methionine and homocysteine levels are elevated.

**Roles in This Reflective Roleplay**

1. Dr. Malar – Senior Pediatrician
2. Mr. Ravi – Final-Year Medical Student
3. Rithvik – Patient
4. Ms. Radha – Grandmother and Caregiver
5. Ms. Veena – School Teacher (Telephonic Consultation)

**Key Clinical and Biochemical Points**

1. Homocystinuria is caused by deficiency of cystathionine beta-synthase
2. Leads to accumulation of homocysteine and methionine
3. Clinical features include marfanoid habitus, downward lens dislocation, developmental delay, and thrombotic risk
4. Diagnosis is confirmed through plasma amino acid analysis
5. Some cases respond to vitamin B6; management includes methionine restriction and long-term metabolic follow-up
6. The condition is inherited in an autosomal recessive pattern; family screening and genetic counseling are important

**Dilemma to Resolve**

1. How do you explain an inherited metabolic disorder to a caregiver who has lived with uncertainty and guilt?
2. How do you coordinate dietary management, vascular risk reduction, and educational support?
3. How should family members be counseled regarding recurrence risk and sibling evaluation?

**Reflection Questions**

1. What clinical features distinguished this from isolated developmental delay?
2. How did understanding the methionine metabolism pathway clarify the risk of thrombosis?
3. What communication strategies are important when discussing inherited metabolic conditions?
4. How can coordination between healthcare and school systems improve outcomes?
5. In what ways does the artwork illuminate chronic vulnerability that is not immediately visible in laboratory data?
6. Where does the artwork fail to represent the biochemical and vascular risks associated with homocystinuria?

**Dr. Malar – Senior Pediatrician**

You recognize the constellation of marfanoid features, developmental delay, and thrombotic history as suggestive of homocystinuria.

You guide **Mr. Ravi** to reason through:

- Methionine metabolism
- The role of cystathionine beta-synthase
- How homocysteine damages vascular endothelium
- Why thrombosis can occur in childhood

You explain to **Ms. Radha** that this is an inherited metabolic condition that can be managed with diet and monitoring.

You discuss the importance of vitamin B6 responsiveness testing and referral for genetic counseling.

**Mr. Ravi – Final-Year Medical Student**

You take the history of present illness, perform an examination, and speak with **Ms. Veena** over the phone.

You organize findings:

- Developmental history
- Thrombotic episode
- Visual changes
- Skeletal features

When prompted by **Dr. Malar**, you explain in simple language how the body is unable to properly convert homocysteine, leading to accumulation and complications.

**Rithvik – Patient**

You are quiet and easily fatigued. You struggle with reading and coordination. Your leg still feels heavy at times. When spoken to kindly, you respond softly. You do not fully understand the medical discussion but sense concern.

**Ms. Radha – Grandmother and Caregiver**

You have cared for Rithvik since he was two years old. You were told he was “slow” and “fragile.” You fed him well, took him to clinics, but no one gave you a name for his condition.

You ask:

- “Did I miss something?”
- “Is there medicine that will help?”
- “Will other children in our family have this?”

You need clear, compassionate explanation and a structured plan.

**Ms. Veena – School Teacher**

You describe Rithvik’s difficulties with attention and stamina. You want guidance on classroom accommodations.

You ask:

- “How can we help him learn without exhausting him?”
- “What signs should we watch for?”

You represent collaboration beyond the clinic.

**ART – 12**

**STRUCTURED PEER ROLEPLAY ACTIVITY**

**Context of the Artwork**
A girl sits motionless, illuminated yet inwardly guarded. Her skin appears very pale, her hair light, and her gaze steady but distant. She holds a pale rose that seems slightly wilted. The image evokes visibility and vulnerability.

This reflective roleplay explores oculocutaneous albinism as a lifelong genetic condition with medical and psychosocial implications.

**Learning Objectives**
By the end of this session, learners will be able to

1. Describe patient suffering through visual and narrative cues.
2. Describe the biochemical basis and key clinical features of disease presented in the case scenarios.
3. Use Visual Thinking Strategies (VTS) and structured peer roleplays to reflect on patient and caregiver perspectives on illness and suffering.
4. Demonstrate empathetic communication, ethical reasoning, and collaborative clinical decision-making in the context of a biochemical disorder through a structured peer roleplay and guided reflection.
5. Reflect on emotions, communication, and the meaning of suffering using a structured reflection format.

**Clinical Scenario**
A ten year old girl named **Anita** is brought to a dermatology clinic by her aunt. She has very light skin and hair, and squints often in bright light. Her teachers say she cannot see the board and avoids going outside during recess. She is quiet, withdrawn, and prefers books over friends. Her aunt says, “Other children make fun of her. Some even say she’s cursed.”

On examination, she has pale irises, visible nystagmus, dry skin, and patches of sun damage on her arms. You suspect **oculocutaneous albinism**. Her family has not heard of it before. They want to know if it’s dangerous, if it will spread, or if she can “grow out of it.”

**Roles in This Reflective Roleplay**

1. Dr. Revathi – Dermatologist
2. Mr. Naren – Third-Year Medical Student
3. Anita – Patient
4. Ms. Kalyani – Aunt and Caregiver
5. Ms. Meenal – School Counsellor

**Key Clinical and Biochemical Points**

1. Oculocutaneous albinism is typically inherited in an autosomal recessive pattern
2. Mutations affect melanin biosynthesis, commonly involving tyrosinase
3. Reduced melanin leads to hypopigmentation, photophobia, nystagmus, and decreased visual acuity
4. Increased risk of sun damage and skin malignancy requires lifelong photoprotection
5. Management includes sun protection, tinted lenses, visual aids, and regular ophthalmologic and dermatologic care
6. Genetic counseling is important for family education and future reproductive planning
7. Psychosocial stigma and cultural misunderstanding can significantly impact quality of life

**Dilemma to Resolve**

1. How do you explain a lifelong genetic condition without framing it as a defect?
2. How do you address stigma and misinformation while reinforcing dignity and capability?
3. What steps can schools and families take to create a supportive environment?

**Reflection Questions**

- How did understanding melanin biosynthesis clarify the clinical presentation?
- What distinguishes a genetic difference from a progressive disease process?
- What communication strategies reduce stigma in visible inherited conditions?
- How can interdisciplinary collaboration improve long-term outcomes?
- In what ways does the artwork illuminate the experience of visible difference beyond clinical findings?
- Where does the artwork fail to represent the molecular and genetic basis of oculocutaneous albinism?

**Role-Specific Instructions**

**Dr. Revathi – Dermatologist**

You recognize the clinical pattern of oculocutaneous albinism.

You ask **Mr. Naren** to explain:

- The role of tyrosinase in converting tyrosine to melanin
- Why reduced melanin affects both skin and ocular development
- Why this condition is inherited and not acquired

You explain to **Ms. Kalyani** that this is a genetic condition present from birth, not contagious and not progressive in a degenerative sense.

You outline practical management strategies: sunscreen, protective clothing, tinted glasses, regular eye evaluations, and psychosocial support.

You introduce the concept of genetic counseling.

**Mr. Naren – Third-Year Medical Student**

You perform a focused dermatologic and ophthalmologic examination under supervision.

When prompted, you describe the biochemical pathway of melanin synthesis and how tyrosinase deficiency alters pigmentation.

You assist in explaining school accommodations to **Ms. Meenal**, including seating arrangements, large-print materials, and sun-safe practices.

**Anita – Patient**

You are aware that others notice your appearance. Bright light makes your eyes uncomfortable. You enjoy reading but struggle to see clearly at a distance.

You ask, “Why am I different?” and “Will I always look like this?”

You respond positively when addressed directly and respectfully.

**Ms. Kalyani – Aunt and Caregiver**

You are Anita’s primary guardian. You have heard myths and social stigma surrounding her appearance.

You ask:

- “Is this dangerous?”
- “Will it get worse?”
- “Could future children have this?”

You are open to understanding genetic inheritance and practical care steps.

**Ms. Meenal – School Counsellor**

You seek guidance on how to support Anita academically and socially.

You ask:

- “How can we adjust the classroom?”
- “How do we prevent bullying?”

You collaborate to ensure Anita’s safety, inclusion, and confidence.

**ART – 13**

**STRUCTURED PEER ROLEPLAY ACTIVITY**

**Context of the Artwork**

A woman gently supports a tired child across her lap. The child appears weary, limbs relaxed, head resting against the caregiver’s chest. The atmosphere suggests recurrent illness and caregiving fatigue. The image does not depict specific phenotypic features but reflects the emotional weight of chronic childhood vulnerability and sustained caregiving.

In this reflective roleplay, the artwork is used to explore the lived experience of raising a child with a chromosomal condition such as Down syndrome, even though the visual details of the syndrome are not explicitly represented.

**Learning Objectives**
By the end of this session, learners will be able to

1. Describe patient suffering through visual and narrative cues.
2. Describe the biochemical basis and key clinical features of disease presented in the case scenarios
3. Use Visual Thinking Strategies (VTS) and structured peer roleplays to explore perspectives on illness and suffering from the eyes of patient and caregivers.
4. Participate in a structured peer roleplay and guided reflection to demonstrate empathetic communication, ethical reasoning, and collaborative clinical decision-making in the context of a biochemical disorder.
5. Reflect on emotions, communication, and the meaning of suffering using a structured reflection format.

**Clinical Scenario**
A 3-year-old girl, **Amulya**, is brought to a pediatric clinic by her mother, **Ms. Sandhya**. She has a history of frequent respiratory infections, developmental delays, and generalized hypotonia. She was diagnosed with Down syndrome at birth but has had limited structured follow-up because of travel distance and financial constraints.

Ms. Sandhya reports, “She has not been able to sit without support. I am worried something else is wrong.”

On examination, Amulya has hypotonia, a flat nasal bridge, epicanthal folds, a single palmar crease, and open-mouth posture. She appears listless and has signs of lower respiratory tract infection. She is admitted for management of suspected pneumonia.

This case focuses on both acute illness and the broader context of delayed developmental intervention and systemic barriers to care.

**Roles in This Reflective Roleplay**

1. Dr. Nivedita – Senior Pediatric Consultant
2. Mr. Rahul – Medical Student
3. Amulya – Patient
4. Ms. Sandhya – Mother and Primary Caregiver
5. Sister Latha – Pediatric Rehabilitation Nurse

**Key Clinical and Biochemical Points**

1. Down syndrome is caused by trisomy of chromosome 21
2. Results from meiotic nondisjunction, leading to an extra copy of genetic material
3. Clinical features include hypotonia, characteristic facial features, developmental delay, and intellectual disability
4. Common complications include congenital heart defects, thyroid dysfunction, recurrent infections, and hearing loss
5. Early multidisciplinary intervention (physiotherapy, speech therapy, educational support) significantly improves functional outcomes
6. Genetic counseling is important for recurrence risk discussion and family planning

**Dilemma to Resolve**

1. How do you discuss delayed developmental intervention without assigning blame?
2. How do you construct a realistic, sustainable care plan in a resource-limited setting?
3. How can a long-term multidisciplinary support system be built around Amulya?

**Reflection Questions**

1. How does trisomy 21 affect development at the chromosomal and cellular level?
2. What distinguishes acute medical illness from long-standing developmental needs in this case?
3. How can clinicians balance honesty about limitations with preservation of hope?
4. What systemic barriers influence long-term outcomes in children with chromosomal disorders?
5. In what ways does the artwork illuminate caregiver endurance beyond physical findings?
6. Where does the artwork fail to represent the specific phenotypic and genetic features of Down syndrome?

**Dr. Nivedita – Senior Pediatric Consultant**

You recognize two distinct issues:

1. An acute respiratory infection requiring immediate medical management.
2. Long-standing developmental needs associated with trisomy 21 that have not received structured intervention.

You guide **Mr. Rahul** to explain:

- The chromosomal basis of trisomy 21
- How gene dosage imbalance affects development
- Why hypotonia contributes to feeding difficulty and delayed milestones

You clearly distinguish Amulya’s biological condition from caregiver fatigue or systemic gaps in access to care.

You speak to **Ms. Sandhya** without blame, outlining what interventions can still be initiated and emphasizing that developmental progress remains possible.

You involve **Sister Latha** to establish rehabilitation and community linkage.

**Mr. Rahul – Medical Student**

You are asked to explain the chromosomal basis of Down syndrome in simple terms.

You describe how an extra copy of chromosome 21 affects development.

You organize care needs into:

- Acute medical treatment
- Developmental therapy referral
- Hearing and thyroid screening
- Cardiac evaluation if not previously done

You learn to speak about chromosomal conditions without reducing the child to a diagnosis.

**Amulya – Patient**

You are quiet and easily fatigued. You respond to sound and familiar voices. You smile when your mother sings.

You do not understand medical discussions but sense comfort and distress in others.

**Ms. Sandhya – Mother and Primary Caregiver**

You have cared for Amulya through repeated infections and developmental delays. You left employment to focus on her care. You feel exhausted but deeply devoted.

You say, “She is my whole world. I just want her to get better.”

You ask whether she will walk, speak, or go to school. You need clarity without judgment.

**Sister Latha – Pediatric Rehabilitation Nurse**

You coordinate physiotherapy, speech therapy referrals, and disability support services.

You explain available community programs and help structure follow-up.

You reinforce that early intervention can still improve functional independence.

**ART – 14**

**STRUCTURED PEER ROLEPLAY ACTIVITY**

**Context of the Artwork**
A woman lies alone on a hospital bed, bleeding. Six symbolic images float around her, each connected by red lines: a fetus, a stylized pelvic bone, a medical instrument, a snail, a purple orchid, and a salmon-colored anatomical form representing her torso and internal reproductive organs. The orchid has often been interpreted as symbolizing the uterus and femininity, while the torso fragment reflects bodily vulnerability. Together, these elements represent reproductive loss, physical trauma, and emotional fragmentation.

The painting was created after Frida Kahlo’s miscarriage, portraying not only biological loss but also identity rupture and embodied grief.

**Learning Objectives**
By the end of this session, learners will be able to

1. Describe patient suffering through visual and narrative cues.
2. Describe the biochemical basis and key clinical features of disease presented in the case scenarios
3. Use Visual Thinking Strategies (VTS) and structured peer roleplays to explore perspectives on illness and suffering from the eyes of patient and caregivers.
4. Participate in a structured peer roleplay and guided reflection to demonstrate empathetic communication, ethical reasoning, and collaborative clinical decision-making in the context of a biochemical disorder.
5. Reflect on emotions, communication, and the meaning of suffering using a structured reflection format.

**Clinical Scenario**
A 28-year-old woman, **Lalitha**, is admitted following her first miscarriage at 14 weeks of pregnancy. She presents with heavy vaginal bleeding, spontaneous bruising over her arms, and bleeding gums. Her platelet count is **10,000/µL**. Hemoglobin and white blood cell counts are normal. Coagulation profile (PT, aPTT) is within normal limits. She is afebrile and hemodynamically stable but appears weak and emotionally distressed.

She recalls frequent bruising in childhood and reports prolonged bleeding after a dental extraction several years ago. No prior hematologic evaluation was performed. Peripheral smear shows markedly reduced platelets with normal morphology. A diagnosis of immune thrombocytopenic purpura (ITP) is considered after exclusion of secondary causes.

**Roles in This Reflective Roleplay**

1. Dr. Anitha – Consultant Hematologist
2. Dr. Abir – Junior Doctor
3. Lalitha – Patient
4. Ms. Renu – Sister-in-Law
5. Ms. Shyamala – Hospital Social Worker

**Key Clinical and Biochemical Points**

- Immune thrombocytopenic purpura is caused by autoantibodies targeting platelet membrane glycoproteins (commonly GPIIb/IIIa)
- Antibody-coated platelets are destroyed in the spleen
- Platelet count is markedly reduced while coagulation studies remain normal
- Clinical features include easy bruising, mucosal bleeding, menorrhagia, and prolonged bleeding after minor trauma
- Pregnancy may exacerbate ITP and increase bleeding risk
- Management includes corticosteroids, intravenous immunoglobulin, thrombopoietin receptor agonists, and in refractory cases, splenectomy
- Reproductive counseling and hematologic follow-up are essential

**Dilemma to Resolve**

1. How do you explain that a lifelong bleeding tendency may have gone unrecognized?
2. How do you address grief and potential self-blame when diagnosis follows miscarriage?
3. How do you plan future pregnancies safely while balancing medical risk?

**Reflection Questions**

1. What historical clues pointed toward a chronic platelet disorder?
2. How does platelet physiology explain normal coagulation tests in the presence of severe bleeding?
3. What communication challenges arise when diagnosis coincides with pregnancy loss?
4. How can clinicians support both reproductive autonomy and medical safety?
5. In what ways does the artwork illuminate bodily fragmentation beyond laboratory findings?
6. Where does the artwork fail to represent underlying ITP?

**Dr. Anitha – Consultant Hematologist**

You confirm immune thrombocytopenic purpura after excluding secondary causes such as systemic lupus erythematosus, HIV, and hepatitis.

You guide **Dr. Abir** to explain:

- The role of platelet glycoproteins
- Mechanism of immune-mediated platelet destruction
- Why coagulation parameters remain normal
- Why splenic sequestration occurs

You initiate corticosteroid therapy and discuss intravenous immunoglobulin if bleeding worsens.

You speak to Lalitha clearly and compassionately, separating the miscarriage from direct causation while acknowledging increased bleeding risk.

You introduce long-term hematologic follow-up and reproductive planning.

**Dr. Abir – Junior Doctor**

You initially identified severe thrombocytopenia and ruled out:

- HELLP syndrome
- Disseminated intravascular coagulation
- Sepsis-related thrombocytopenia

When prompted by **Dr. Anitha**, you explain platelet physiology in accessible language.

You learn to pause before discussing prognosis, allowing space for grief.

**Lalitha – Patient**

You are grieving and physically exhausted. You alternate between silence and sharp questioning. You ask:

- “Was this because of my blood?”
- “Why did no one find this earlier?”
- “Can I carry a pregnancy safely again?”

You appear withdrawn at times, avoiding eye contact, clutching the bedsheet, and responding slowly when spoken to.

You need clarity, not reassurance without substance.

**Ms. Renu – Sister-in-Law**

You ask practical questions about:

- Family testing
- Recurrence risk
- Timeline for recovery
- Future pregnancy planning

You advocate firmly but respectfully.

**Ms. Shyamala – Hospital Social Worker**

You observe Lalitha’s quiet withdrawal, minimal eye contact, and delayed responses.

You provide structured grief support and offer counseling resources.

You help coordinate follow-up appointments, financial documentation, and leave from work.

You remind the team that complex medical information may need to be revisited after emotional stabilization.

**ART 15**

**STRUCTURED PEER ROLEPLAY ACTIVITY**

**Context of the Artwork**
A woman reclines in a domestic setting, hand placed over her chest. Her posture reflects fatigue rather than dramatic collapse. Another figure leans toward her holding a bottle, suggesting home-based interpretation of symptoms rather than urgent medical care. The scene reflects the historical minimization of women’s symptoms, delayed recognition, and social framing of illness before clinical intervention.

This reflective roleplay explores non–ST elevation myocardial infarction (NSTEMI) in a postmenopausal woman with atypical presentation.

**Learning Objectives**
By the end of this session, learners will be able to

1. Describe patient suffering through visual and narrative cues.
2. Describe the biochemical basis and key clinical features of disease presented in the case scenarios
3. Use Visual Thinking Strategies (VTS) and structured peer roleplays to explore perspectives on illness and suffering from the eyes of patient and caregivers.
4. Participate in a structured peer roleplay and guided reflection to demonstrate empathetic communication, ethical reasoning, and collaborative clinical decision-making in the context of a biochemical disorder.
5. Reflect on emotions, communication, and the meaning of suffering using a structured reflection format.

**Clinical Scenario**
A 53-year-old woman, **Sheela**, arrives at the emergency department with chest heaviness, breathlessness, and upper abdominal discomfort for the past three hours. She has entered the postmenopausal phase of life. She has no known history of coronary artery disease. She initially attributed the symptoms to indigestion and delayed seeking care.

Vital signs are stable. Electrocardiogram shows mild ST-segment depression in anterior leads. Serum troponin T is elevated above the reference range.

The clinical picture is consistent with non–ST elevation myocardial infarction (NSTEMI). She expresses reluctance to remain hospitalized and says, “This happened last month too and the doctor said it was nothing.” Her daughter, **Ms. Lakshmi**, insists on further evaluation.

**Roles in This Reflective Roleplay**

1. Dr. Meera – Emergency Physician
2. Mr. Nikhil – Intern
3. Sheela – Patient
4. Ms. Lakshmi – Daughter
5. Ms. Florence – Cardiology Nurse

**Key Clinical and Biochemical Points**

1. Myocardial infarction in women may present atypically, including fatigue, dyspnea, epigastric discomfort, or nausea
2. Postmenopausal estrogen decline increases cardiovascular risk
3. Troponin I and T are highly specific biomarkers for myocardial cell injury
4. NSTEMI often presents with ST depression or T-wave inversion rather than ST elevation
5. Pathophysiology involves plaque rupture or erosion leading to partial coronary occlusion
6. Immediate management includes antiplatelet therapy, anticoagulation, statins, nitrates, and risk stratification for angiography
7. Gender bias and diagnostic delay contribute to worse outcomes in women

**Dilemma to Resolve**

1. How do you manage a life-threatening condition when the patient minimizes symptoms?
2. How do you distinguish atypical presentation from non-cardiac causes without excessive testing?
3. How do you explain biomarker elevation in a way that builds informed consent about furthering his treatment?

**Reflection Questions**

1. What features suggested myocardial infarction despite absence of classic crushing chest pain?
2. How does troponin release reflect myocardial necrosis at the cellular level?
3. What physiologic changes after menopause increase cardiovascular risk?
4. How can clinicians reduce gender-based disparities in cardiac care?
5. In what ways does the artwork illuminate delayed recognition of women’s illness?
6. Where does the artwork fail to represent NSTEMI?

**Dr. Meera – Emergency Physician**

You recognize NSTEMI based on troponin elevation and ECG changes.

You ask **Mr. Nikhil** to explain:

- Why troponin is specific for cardiac muscle injury
- The ischemic cascade leading to myocardial necrosis
- Why ST depression differs from ST elevation
- Risk stratification principles

You initiate aspirin, anticoagulation, statin therapy, and continuous monitoring.

You explain to Sheela that elevated troponin indicates heart muscle injury and that observation is necessary to prevent progression.

**Mr. Nikhil – Intern**

You review the electrocardiogram, calculate the TIMI risk score, and arrange repeat troponin testing.

When prompted, you explain myocardial oxygen supply-demand imbalance and plaque rupture.

You practice communicating urgency without inducing panic.

**Sheela – Patient**

You are 53 and have entered the postmenopausal phase of life. You have experienced fatigue and breathlessness over recent months but attributed them to aging.

You say, “I don’t want to overreact,” and “It’s probably gas again.”

You are hesitant but receptive when given clear explanation.

**Ms. Lakshmi – Daughter**

You are concerned that prior symptoms were dismissed.

You ask:

- “Can this be a heart attack without severe pain?”
- “What happens if she leaves now?”

You advocate for continued evaluation.

**Ms. Florence – Cardiology Nurse**

You provide structured monitoring and patient education.

You reinforce medication explanations in accessible language.

You assess Sheela’s understanding of the diagnosis and clarify misconceptions to ensure informed participation in care.

You coordinate timely repeat testing and maintain hemodynamic monitoring.
