# Supplementary material for: The Palette of Science and Emotions: Art-Based Learning With Structured Peer Role-Plays for Early Clinical Exposure in Biochemistry
Source: MedEdPORTAL. 2026 May 19;22:11601. doi: 10.15766/mep_2374-8265.11601 (PMC13183865; doi:10.15766/mep_2374-8265.11601)
Supplement: Supplementary file 1 — Faculty Orientation.pptxCurated Artworks.docxActivity Instructions.docxRole-Play Resources.docxFacilitator Guide.docxPersonal Reflection Questionnaire.docxEvaluation Questionnaire.docxSemistructured Interview Guide.docxPostsession Assessment.docxConfidence Questionnaire.docx [file mep_2374-8265.11601-s001.zip › _Educational Summary Report_11601.pdf]

MedEdPORTAL
THE AAMC JOURNAL OF TEACHING AND LEARNING RESOURCES

Original Publication Open Access

The Palette of Science and Emotions: Art-Based Learning With Structured Peer Role-Plays for Early Clinical Exposure in Biochemistry

Krishna Mohan Surapaneni, MHPE, PhD*

*Corresponding author: krishnamohan.surapaneni@gmail.com

Abstract

Introduction: Biochemistry is often regarded as conceptually dense yet emotionally disconnected, limiting its relevance to patient care. To bridge this gap, an art-based learning intervention was developed to connect biochemistry of diseases with empathy, perspective-taking, and ethical awareness through visual thinking strategies, structured peer role-play, and guided reflection. **Methods:** This small-group activity was conducted for 150 first-year medical students over a total duration of 180 minutes. Students were divided into 30 groups and each group received a curated artwork and a biochemical role-play case scenario. Evaluation followed Kirkpatrick's model with a 20-item perception survey and semistructured interviews for level 1 (reaction), postsession assessment, "I used to think, now I think, now I will" reflection, and a 7-item confidence questionnaire for level 2 (learning). **Results:** Students reported high levels of engagement across all components, particularly appreciating visual thinking strategies and structured peer role-play. The postsession assessment score was 24.1 ($SD = 3.0$) out of 30 [$P < .001$]. Thematic analysis of documented reflections yielded 16 distinct themes representing the shift in learners' perspectives and their growing commitment to empathic patient care. **Discussion:** The integration of art and role-play into early biochemistry teaching effectively supported students in linking molecular mechanism of diseases with human experiences of illness. It also encouraged thoughtful participation and facilitated emotional and ethical awareness. While the approach proved feasible and impactful within this setting, its broader implementation may depend on institutional support and faculty readiness for arts-integrated pedagogy.

Keywords
Visual Thinking Strategies, Medical Humanities, Reflection, Communication Skills, Museum Arts, Simulation, Early Clinical Exposure, Biochemistry Education

Educational Objectives

By the end of this learning activity, students will be able to:

1. Describe patient suffering through visual and narrative cues.
2. Describe the biochemical basis and key clinical features of disease presented in the case scenarios.
3. Use visual thinking strategies and structured peer role-plays to reflect on patient and caregiver perspectives on illness and suffering.
4. Demonstrate empathetic communication, ethical reasoning, and collaborative clinical decision-making in the context of a biochemical disorder through a structured peer role-play and guided reflection.
5. Reflect on emotions, communication, and the meaning of suffering using a structured reflection format.

Citation:
Surapaneni KM. The palette of science and emotions: art-based learning with structured peer role-plays for early clinical exposure in biochemistry. *MedEdPORTAL*. 2026;22:11601.
https://doi.org/10.15766/mep_2374-8265.11601

Introduction

In medical school, biochemistry is often perceived as an abstract subject, rich in molecular complexity yet emotionally distant from the lived experiences of patients.[1] This disconnect is especially pronounced in early years of training, where students begin to acquire the language of science but have not yet developed the lens to see suffering through a clinical or humanistic frame.[2] The problem is not in the rigor of the subject, but in the absence of spaces where foundational sciences intersect meaningfully with empathy, narrative, and the moral imagination. While there are several clinical integration and patient-centered care modules to humanize basic science learning, the challenge remains in how we teach biochemistry in ways that make illness visible, suffering palpable, and the patient present.[3,4]

Art-based learning (ABL) is an emerging and impactful pedagogical approach in medical education that fosters

Copyright © 2026 Surapaneni. This is an open-access publication distributed under the terms of the Creative Commons Attribution license.

transformative learning by bridging clinical understanding with emotional and reflective insight.[5] Rooted in the deliberate use of visual art and interpretive inquiry, ABL offers a multidimensional space where students can encounter multiple emotions and senses through a lens of observation and narrative reflection.[6]

A central methodology within ABL is visual thinking strategies (VTS). It is a structured, inquiry-driven approach originally developed in museum education and now increasingly applied in medical training.[7] VTS invites learners to engage with an image through slow, reflective looking, followed by facilitated discussion using open-ended prompts specifically drafted for this approach, like “What’s going on in this picture?” “What do you see that makes you say that?” and “What more can we find?”[8] This approach emphasizes evidence-based interpretation, multiple perspectives, and active listening skills that can be considered critical to both clinical reasoning and compassionate care.[9] In the context of medical education, VTS can be applied not only to sharpen observational acuity but also to open space for students to explore emotional responses, implicit biases, and the moral dimensions of patient care.[10]

There are 2 impactful *MedEdPORTAL* publications on the use of visual arts for reflecting learning and enhancing perspective-taking among students.[11,12] These initiatives have demonstrated how structured engagement with artwork can deepen students’ awareness, strengthen their observational skills, and foster attitudinal growth. What remains underexplored is the deliberate integration of visual arts into the teaching of biochemical concepts, particularly during early medical training. The current educational activity addresses this critical gap by embedding curated visual artworks and integrating them with structured role-play exercises to portray illness and suffering. Through this, students are invited not only to learn the underlying biochemical processes but also to consider how these processes manifest in the lives of patients and families and to engage in real-life communication, developing empathy, understanding, and compassion.

Methods

The competency-based medical education curriculum by the National Medical Commission of India is implemented at our institution. The curriculum includes a key component called early clinical exposure (ECE), which is intended to expose first-year medical students to patient interactions. Every foundational subject, including anatomy, physiology, and biochemistry, has been allotted 30 hours of ECE. I conducted this activity as part of the biochemistry ECE during the preclinical phase, after students had completed core instruction in metabolic pathways and molecular mechanisms. Although students had prior exposure to case-based learning, they had not previously participated in structured ABL or formal VTS. Before the actual session, I conducted an orientation session for the faculty facilitators (Appendix A). Students were not expected to have any prior preparation.

For this activity, I curated 15 powerful artworks depicting themes of illness, vulnerability, and human suffering from openly accessible sources (Appendix B), such as *The Sick Child* by Edvard Munch and *Death and the Maiden* by Egon Schiele. Each artwork was then printed as a museum-style display board, mounted with description text, and placed prominently to simulate a gallery-viewing experience within the learning environment. This educational approach was informed by the prism model for arts and humanities in medical education, which identifies 4 core pedagogical functions: skills development, perspective-taking, personal insight, and social advocacy.[13] Of these, the present intervention primarily focused on perspective-taking and personal insight.

I conducted this multimethod educational activity with 150 first-year medical students after obtaining written informed consent form from all. Instructions were distributed to students prior to the session (Appendix C). The 180-minute activity was structured as (i) a VTS session, (ii) structured peer role-play, (ii) guided reflection, and (iv) plenary discussion. Students were organized into 30 small groups, each consisting of 5 students, and assigned a biochemistry/foundational medical science faculty facilitator. Each group received a single curated artwork at the beginning. After the VTS session, groups received a clinical case scenario for a biochemical disorder, role descriptions for role-play (eg, physician, patient, caregiver, nurse, or counselor), dilemmas to resolve, and reflection prompts (Appendix D). As there were 30 groups, each artwork—case set was allocated to 2 groups to maintain equal distribution. Faculty were given a checklist guide to mark the completion of all activities (Appendix E). Faculty were required to be trained in conducting the VTS session.

The session began with the VTS component (30 minutes), during which students engaged in slow, intentional observation of their group’s assigned artwork. No diagnostic labeling or biochemical interpretation was introduced at this stage. Faculty provided a disclaimer at the outset, emphasizing that the artwork was not intended to depict or diagnose a specific disease. Using VTS prompts, students engaged in open-ended observation of visible physical, emotional, and social context cues within the artwork and documented key observations. Interpretations were

Copyright © 2026 Surapaneni. This is an open-access publication distributed under the terms of the Creative Commons Attribution license.

grounded in visual evidence, and multiple perspectives were encouraged. This phase aimed to cultivate empathy, perspective-taking, and observational acuity.

Following the VTS session, students transitioned into the structured peer role-play exercise (45 minutes). The clinical scenario was introduced at this stage. Groups were instructed to revisit their documented visual observations from the VTS discussion to identify elements from the artwork which aligned with the patient presentation and which did not. Each group was required to articulate the likely diagnosis, explain the underlying biochemical mechanism, correlate it with clinical findings, and enact the scenario to address the ethical dimensions of decision-making, particularly in situations involving uncertainty, limited resources, or caregiver distress. Students were allowed to have 1 biochemistry textbook per group to support preparation.

The next phase involved faculty-guided reflection (25 minutes), during which students reflected on the questions given in their role-play materials for deeper correlation of the art, role-play, and biochemistry. They also reflected their learning using the "I used to think, now I think, now I will" framework via an online SurveyMonkey form (Appendix F) and prepared a summary of their case.[14] In the final plenary session (60-80 minutes), groups with the same clinical cases were paired and presented to the entire class, outlining the likely diagnosis, underlying biochemical mechanism, key clinical correlations, and ethical considerations. This peer-sharing format ensured that all students were exposed to all 15 case scenarios and their associated biochemical concepts.

Our evaluation plan was guided by the Kirkpatrick model.[15] Immediate learner reaction (level 1) was measured through a 20-item online evaluation (Appendix G) questionnaire administered via SurveyMonkey at the end of the session, and learners' experiences were gauged qualitatively through semistructured small-group interviews. Purposively sampled students were invited via email and recruited ensuring diversity in participants. Semistructured interviews were conducted within 2 weeks of the session by a faculty member not involved in this activity to minimize social desirability bias. Interviews took place in a private academic setting, lasted approximately 20-30 minutes, were audio recorded with consent, and followed an interview guide (Appendix H).

Further, to assess learning (level 2), at the end of the plenary session, each student completed a paper-based postsession assessment comprising 15 clinical reasoning questions (short answer scored using a 0-2 analytic rubric) (Appendix I). Additionally, a 7-item confidence questionnaire on a 5-point Likert scale (Appendix J) was administered electronically via SurveyMonkey to evaluate perceived confidence in applying biochemical knowledge and understanding patient suffering.

The mean and standard deviations of all continuous variables were reported using univariate statistics. For normally distributed data, group differences were evaluated using ANOVA and *t* tests; for nonnormally distributed variables, the Mann-Whitney U and Kruskal-Wallis tests were employed. Stata/BE version 18.5, with statistical significance set at $P < .05$, was used to analyze the data. For qualitative data, the audio recordings were transcribed verbatim and analyzed using reflexive thematic analysis as described by Clarke and Braun.[16] Two data analyzers independently reviewed the transcripts and reflections, performed inductive line-by-line coding, and developed themes through iterative comparison and refinement. Discrepancies were resolved through discussion, and an audit trail was maintained to ensure analytic rigor and transparency. Ethical approval was obtained from Panimalar Medical College Hospital and Research Institute's Institutional Human Ethics Committee (Approval No. PMCHRI-IHEC-264; April 2, 2025).

Results

A total of 150 students participated (61% females and 39% males). For the end of activity evaluations, the response rate was 97.5% ($n = 146$). Participants' mean age was 20 years ($SD = 2.1$). When inquiring about previous experience in ABL, this was the first exposure for all students. For the 15-item postsession assessment (maximum score: 30), the mean score was 24.1 ($SD = 3.0$). Overall, 82% of students demonstrated satisfactory reasoning, whereas 14% required further conceptual strengthening and 4% demonstrated inadequate clinicobiochemical reasoning.

The 20-item evaluation questionnaire was spilt into 5 items for VTS evaluation; 8 items for structured peer role-play; 4 items for guided reflection; and 3 items for comparing with traditional lecture, case-based learning, and overall evaluation. Students responded positively across all components of the session, indicating a high level of engagement and perceived relevance. They particularly reported that the VTS session with the discussion was highly effective in supporting meaning-making and the importance of peer perspectives in expanding their views. Additionally, the structured peer role-play activity was perceived as immersive and realistic, noting that the opportunity to assume patient, family, and provider perspectives deepened their insight into the complexities of care. The structured

Copyright © 2026 Surapaneni. This is an open-access publication distributed under the terms of the Creative Commons Attribution license.

reflective exercise was perceived to be highly effective in prompting the learners to move beyond descriptive wording and truly articulate shifts in perspective. Students reported high perceived gains in confidence when the activity was compared with traditional lecture-based sessions ($M = 4.63$, $SD = 0.21$). When compared with existing small-group case-based learning in ECE at our institution ($M = 4.10$, $SD = 0.35$), ratings were also highly positive, suggesting that students experienced this approach as engaging, meaningful, and effective within the continuum of active learning formats. The mean student responses to each item are detailed in the Table.

Regarding the confidence level, there was a notable statistically significant improvement across all 7 areas ($P < .001$). The lowest presession mean scores (out of 5) were observed in students' observation skills (2.0; Figure 1) and thinking from the patient and family perspectives (1.9). The highest postsession mean score was recorded for thinking from the patient and family perspective (4.5) and applying my basic science knowledge like biochemistry to patient care (4.5).

Students' reflections were analyzed ($n = 146$) until thematic saturation was achieved, and 16 distinct themes were identified: 5 under the category "I used to think," 5 under "Now I think," and 6 under "Now I will," as shown in Figure 2.

Within the "I used to think" domain, many students described (i) diagnosis as the final goal ($n = 108$), viewing clinical work as complete once a diagnosis was reached. Closely linked was (ii) laboratory data as the primary authority ($n = 96$), where students acknowledged privileging numbers over narratives, often relying on investigations as the main source of decision-making. Under (iii) communication as technical explanation ($n = 84$), learners equated competence with accuracy, focusing on delivering correct information rather than understanding patient concerns. Another theme, (iv) art as unrelated to medical learning ($n = 72$), reflected a perception that art had little relevance to clinical training. Finally, (v) medicine as rapid problem-solving ($n = 117$) revealed a performance-oriented mindset, with emphasis on efficiency over reflection. These themes suggested a predominantly biomedical, efficiency-driven orientation shaped by early academic exposure.

In contrast, the "Now I think" reflections demonstrated perceptual and conceptual shifts. Under (vi) illness as lived and layered ($n = 101$), students articulated a broader understanding, recognizing the emotional and social dimensions of illness. The theme of (vii) biochemistry as an explanatory bridge ($n = 94$) reflected on importance of integration, linking molecular mechanisms with patient experience. In the next theme, (viii) listening as an intervention ($n = 88$), students reflected on the importance of listening and silence, acknowledging communication itself as therapeutic. Students also recognized (ix) multiplicity of perspectives in care ($n = 79$), appreciating the roles of patients, families, and health care teams. Finally, (x) emotional awareness

Table. Evaluation of the ABL Session: Mean Student Response Analysis

Serial Number	Survey Item	*M* (*SD*)
1	The Visual Thinking Strategies session helped me notice emotions, suffering, and important details in the artwork.	4.18 (0.22)
2	The discussion helped me understand the emotions shown in the artwork more deeply.	4.65 (0.36)
3	The session helped me think beyond what is visible and imagine the patient's experience.	4.10 (0.18)
4	Listening to others' observations gave me new ways of seeing and thinking.	4.25 (0.57)
5	The VTS session helped me appreciate how observation skills are important in clinical practice.	4.33 (0.49)
6	The structured peer role-play helped me understand the emotions and suffering experienced by patients and families.	4.28 (0.29)
7	Taking on a specific role helped me think more clearly about real clinical situations.	4.71 (0.17)
8	The interaction with other roles made the structured peer role-play feel realistic and engaging.	4.54 (0.43)
9	I was able to communicate and collaborate effectively with my group during the structured peer role-play.	4.52 (0.46)
10	The structured peer role-play helped me practice empathy and active listening.	4.72 (0.31)
11	The clinical problem linked to the artwork made me think deeply about real life patient care.	4.61 (0.31)
12	The activity helped me understand how biochemistry knowledge applies in real patient experiences.	4.45 (0.43)
13	The activity helped me understand the underlying biochemical mechanisms responsible for the patient's clinical features.	4.65 (0.43)
14	The reflection exercise helped me become more aware of my original thoughts and assumptions.	4.17 (0.54)
15	The reflection helped me notice how my understanding of illness and communication changed after the session.	4.77 (0.22)
16	The "I used to think, now I think, now I will" format made me think about how I will improve in future.	4.37 (0.28)
17	The reflection made me realize the importance of understanding patient emotions, suffering, and communication skills in real practice.	4.53 (0.41)
18	Compared to traditional lecture-based sessions in Biochemistry, this activity improved my confidence in applying biochemical concepts to clinical scenarios.	4.63 (0.21)
19	Compared to small group case-based learning in ECE, this activity enhanced my engagement and depth of understanding.	4.10 (0.35)
20	Overall, the session helped me connect observation, empathy, communication, and clinical thinking in a meaningful way.	4.62 (0.33)

Abbreviations: ABL, art-based learning; ECE, early clinical exposure; VTS, visual thinking strategies.

Copyright © 2026 Surapaneni. This is an open-access publication distributed under the terms of the Creative Commons Attribution license.

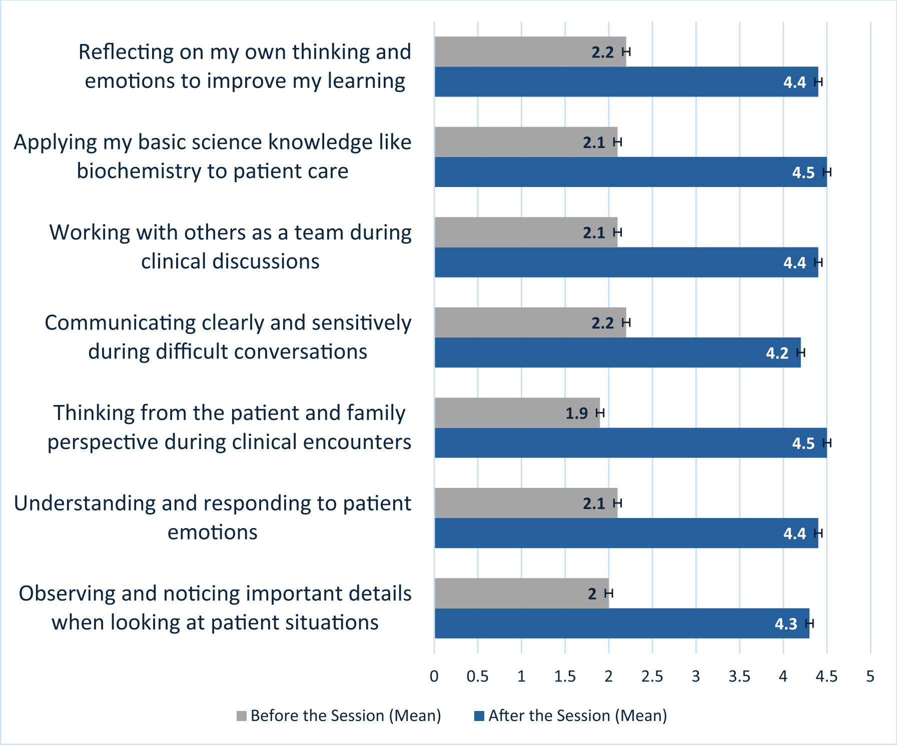

Figure 1. Comparison of student confidence before and after the activity.

in reasoning ($n = 74$) highlighted reflexivity, with students becoming more aware of their own responses in clinical situations. These themes indicated movement from efficiency-driven orientation toward reflective, relational engagement.

The “Now I will” statements translated these insights into intended practice. Under (xi) observe beyond symptoms ($n = 92$), students committed to attentiveness, including nonverbal and contextual cues. The theme (xii) integrate mechanism with meaning ($n = 89$) captured deliberate linkage of science and care, connecting biochemical knowledge with patient narratives. In (xiii) communicate with sensitivity ($n = 83$), learners emphasized tone and pacing, ensuring empathy alongside clarity. Recognition of shared care responsibilities emerged under (xiv) include family and allied voices ($n = 78$), valuing collaborative approaches to care. Reflection itself became intentional practice under (xv) pause for a moment ($n = 70$), allowing time for thoughtful consideration after patient encounters. Finally, (xvi) sustain humility in uncertainty ($n = 65$) reflected professional growth, accepting uncertainty as part of clinical practice.

Further, in the semistructured small-group interviews ($n = 110/150$), students described the session as immersive and thought-provoking, comparing it with usual lecture formats. Many spoke about how playing a role shifted their engagement with the case. One student reflected, “When I had to speak as the patient’s relative, I realized how much uncertainty families sit with. It wasn’t just about getting the diagnosis right.” Another commented, “I usually focus on lab values first. This time, I paid attention to expressions and silence.”

The integration of biochemical reasoning within emotionally layered scenarios was repeatedly noted as meaningful. A participant shared, “We usually learn only the pathway, but when I could understand how really it manifests as a disease, it deepened by knowledge.” Several students described becoming more attentive to communication nuances. “We discussed tone and body language in a way we never do in regular case discussions,” one said. Overall, students described the experience as reflective, clinically anchored, and memorable.

Discussion

This educational activity successfully demonstrated the potential of ABL to transform biochemistry education into a space where empathy, ethical awareness, understanding perspectives, and clinical reasoning can begin to take root. The deliberate integration of VTS, structured peer role-plays, and reflection

Copyright © 2026 Surapaneni. This is an open-access publication distributed under the terms of the Creative Commons Attribution license.

MedEdPORTAL
THE AAMC JOURNAL OF TEACHING AND LEARNING RESOURCES

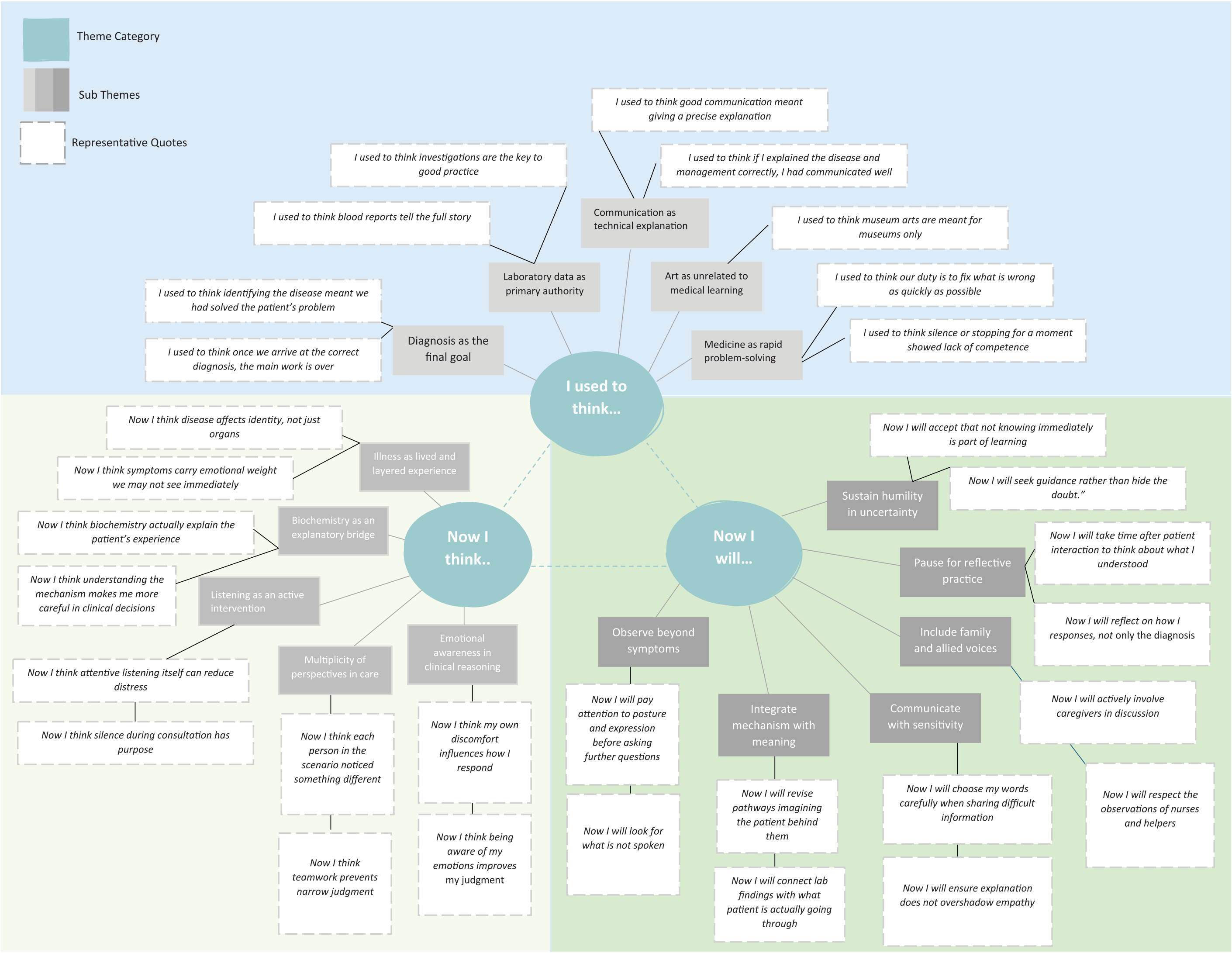

Figure 2. Thematic analysis of the "I used to think, now I think, now I will" reflection.

into the early biochemistry curriculum enabled first-year medical students to transcend the confines of rote molecular understanding and engage with illness as a lived, multifaceted experience. The results, both quantitative and qualitative, suggest that the activity meaningfully addressed the pedagogical gap.

Through the development and implementation of this activity, many key lessons were learned. First, our findings support that the use of artwork as a narrative entry point, especially when presented in a museum-style format, is an effective approach for suspending assumptions and encouraging interpretive inquiry.[17] Second, creating a safe psychological space is important for students to open up.[6] Initially, students were hesitant and provided 1-word answers. Many students saw illness mainly through concepts and lab values. But as they spent time with the artwork and listened to each other, they started to see the human side and what it might feel like to live with pain, fear, or uncertainty. This shift happened as faculty gently introduced the diseases, asking students to link observations from VTS discussion and explaining each expression and emotion. Instead of questioning each student individually, facilitators established a group norm of open observation, making it clear that there were no right answers and no obligation to speak. This approach reduced pressure, respected silence, and gradually encouraged students to engage more authentically.

The structured reflection format ("I used to think, now I think, now I will") provided an accessible yet introspectively rich way

Copyright © 2026 Surapaneni. This is an open-access publication distributed under the terms of the Creative Commons Attribution license.

of meaning-making, allowing learners to track their own evolution in perspective. However, even though the design fostered high engagement and thoughtful reflection, its generalizability may be affected in some contexts due to requirements of VTS-trained faculty. Yet, this may be addressed through brief, targeted faculty development workshops or even by invited trained museum educators, especially in high-resource settings. A limitation of the evaluation of our activity is that increased student confidence cannot be attributed exclusively to the artwork. The activity incorporated multiple active learning elements, each of which may have contributed to the observed outcomes. In the absence of a comparison group without the visual component, causal attribution to artwork alone remains limited.

Looking ahead, this educational approach shows strong promise for wider use in medical education. Even though it was implemented in the context of biochemistry, the same model can be adapted for other foundational science and clinical subjects. Such an approach will provide benefit by helping students connect scientific content with patient emotions, social contexts, and ethical challenges. Additionally, this activity was conducted within a single institutional context, and certain implementation elements may be context specific. For example, students were permitted access to a shared physical biochemistry textbook during role-play preparation, whereas other institutions may rely primarily on electronic resources. Although this logistical difference does not alter the core pedagogical design, adaptation may be required across settings.

In our future iterations, we intend to explore and evaluate how repeated exposure to such sessions shapes long-term skills like empathy, observation, and reflective thinking (level 3 of Kirkpatrick's evaluation). With appropriate support, this approach offers a meaningful way to bring humanism into scientific learning and to prepare students to not only know the science of illness but also understand what it means to care for those who live with it.

Appendices

A. Faculty Orientation.pptx
B. Curated Artworks.docx
C. Activity Instructions.docx
D. Role-Play Resources.docx
E. Facilitator Guide.docx
F. Personal Reflection Questionnaire.docx
G. Evaluation Questionnaire.docx
H. Semistructured Interview Guide.docx
I. Postsession Assessment.docx
J. Confidence Questionnaire.docx

All appendices are peer reviewed as integral parts of the Original Publication.

Krishna Mohan Surapaneni, MHPE, PhD: Head and Professor, Department of Medical Education; Professor, Department of Biochemistry, Panimalar Medical College Hospital and Research Institute, ORCID: https://orcid.org/0000-0002-5204-5708

Acknowledgments
I sincerely thank the faculty facilitators—Dr. Mahima Sophia, Dr. Anusha R, Dr. Mahila S, Dr. Sriram RM, Dr. Lakshmi G, Dr. Balabaskaran S, Dr. Vasanthi C, Dr. Balamurali B, Dr. Vishnu Varthini S, Dr. Elavarasi P, Dr. Ilham Jaleel, Dr. Veerapandian S, Dr. Manasa Raj, Dr. Bhuvaneswari C, Dr. Raja Rajeswari Devi R, Dr. Priya G, Dr. Lavanya S, Dr. Sujatha Narayanan, Dr. Surya B, Dr. Siva Priya J, Dr. Kalaiselvi R, Dr. Gayathri Devi D, Dr. Pearlin Bala V, Dr. Emi Goldy E, Ms. Priyadarshini G, Ms. Lavanya R, Dr. Janani Maheswari V. Vyas, Ms. Nithya S, Ms. Meghna S. Manohar, Ms. Nandhini S, and Mr. Jimjebastin S—whose thoughtful engagement and guidance were central to the success of this session. I am especially grateful to the first-year MBBS students for their open-minded participation, reflective contributions, and willingness to explore new ways of learning through art and group role-plays.

Disclosures
None to report.

Funding/Support
None to report.

Ethical Approval
Panimalar Medical College Hospital and Research Institute Institutional Review Board reviewed this project.

References

1. Elhousni Z, Zerhane R, Janati-Idrissi R. A look at biochemistry learning difficulties in the undergraduate level. *Multidisciplinary Sci J*. 2024;7(2):e2025049. https://doi.org/10.31893/multiscience.2025049

2. D'Souza JMP, Raghavendra U, D'Souza DH, D'Souza NDR. Teaching learning of biochemistry in undergraduate medical curriculum: perceptions and opinions of medical students. *Educ Med J*. 2013;5(2):e45-e53. https://doi.org/10.5959/eimj.v5i2.109

3. Kaur A, Kukreja S, Singh T, Kaur M. Early clinical exposure to enhance the learning of biochemistry in first year MBBS students. *Int J Clin Biochem Res*. 2018;5(3):401-405. https://doi.org/10.18231/2394-6377.2018.0084

Copyright © 2026 Surapaneni. This is an open-access publication distributed under the terms of the Creative Commons Attribution license.

4. Puri D. An integrated problem-based curriculum for biochemistry teaching in medical sciences. *Indian J Clin Biochem*. 2002;17(2):52-59. https://doi.org/10.1007/BF02867971

5. Chisolm MS, Kelly-Hedrick M, Stephens MB, Zahra FS. Transformative learning in the art museum: a methods review. *Fam Med*. 2020;52(10):736-740. https://doi.org/10.22454/FamMed.2020.622085

6. Gaufberg E, DiGiovanni Evans B, Rutberg P, Chisolm MS. What's art got to do with it?—transfer of learning in museum-based health professions education. *Int Rev Psychiatry*. 2023;35(7-8):672-681. https://doi.org/10.1080/09540261.2023.2288299

7. Kelly-Hedrick M, Chugh N, Zahra FS, Stephens M, Chisolm MS. Art museum-based teaching: visual thinking strategies. *Acad Med*. 2022;97(8):1249. https://doi.org/10.1097/ACM.0000000000004600

8. Chisolm MS, Duke L, Stephens MB. Visual thinking strategies in medical education: staying open to possibilities. *Acad Med*. 2023;98(3):295. https://doi.org/10.1097/ACM.0000000000005112

9. Poirier TI, Newman K, Ronald K. An exploratory study using visual thinking strategies to improve undergraduate students' observational skills. *Am J Pharm Educ*. 2020;84(4):7600. https://doi.org/10.5688/ajpe7600

10. Ghorbani N, Alizadeh M, Sayarifard A, Khajavi A, Yenawine P. The eagle eyes: an intervention utilizing visual thinking strategies to enhance the observation skills of medical students. *BMC Med Educ*. 2025;25(1):33. https://doi.org/10.1186/s12909-025-06642-9

11. Rezaei S, Childress A, Kaul B, Rosales KM, Newell A, Rose S. Using visual arts education and reflective practice to increase empathy and perspective taking in medical students. *MedEdPORTAL*. 2023;19:11346. https://doi.org/10.15766/mep_2374-8265.11346

12. Neeley M, Acosta LMY, Davidson M, Dewey C. An empathy and arts curriculum during a pediatrics clerkship: impact on student empathy and behavior. *MedEdPORTAL*. 2024;20:11414. https://doi.org/10.15766/mep_2374-8265.11414

13. Moniz T, Golafshani M, Gaspar CM, et al. The Prism Model: advancing a theory of practice for arts and humanities in medical education. *Perspect Med Educ*. 2021;10(4):207-214. https://doi.org/10.1007/S40037-021-00661-0

14. Project Zero. I Used to Think … Now I Think …. Harvard Graduate School of Education; 2019. Accessed March 4, 2026. https://pz.harvard.edu/sites/default/files/I%20Used%20to%20Think%20-%20Now%20I%20Think_1.pdf

15. Kirkpatrick DL. Evaluation of training. In: Craig RL, Bittel LR, eds. *Training and Development Handbook*. McGraw-Hill; 1967:87-112.

16. Clarke V, Braun V. Thematic analysis. *J Positive Psychol*. 2017;12(3):297-298. https://doi.org/10.1080/17439760.2016.1262613

17. Hubard OM. Illustrating interpretive inquiry: a reflection for art museum education. *Curator: Museum J*. 2011;54(2):165-179. https://doi.org/10.1111/j.2151-6952.2011.00079.x

Received: September 13, 2025
Accepted: April 7, 2026
Published: May 19, 2026

Copyright © 2026 Surapaneni. This is an open-access publication distributed under the terms of the Creative Commons Attribution license.
